# Transcriptome differences between enrofloxacin-resistant and enrofloxacin-susceptible strains of *Aeromonas hydrophila*

**DOI:** 10.1371/journal.pone.0179549

**Published:** 2017-07-14

**Authors:** Fengjiao Zhu, Zongying Yang, Yiliu Zhang, Kun Hu, Wenhong Fang

**Affiliations:** 1 National Pathogen Collection Center for Aquatic Animals, Shanghai Ocean University, Shanghai, China; 2 Nanchang Academy of Agricultural Sciences, Nanchang, China; 3 East China Sea Fisheries Research Institute, Shanghai, China; Youngstown State University, UNITED STATES

## Abstract

Enrofloxacin is the most commonly used antibiotic to control diseases in aquatic animals caused by *A*. *hydrophila*. This study conducted *de novo* transcriptome sequencing and compared the global transcriptomes of enrofloxacin-resistant and enrofloxacin-susceptible strains. We got a total of 4,714 unigenes were assembled. Of these, 4,122 were annotated. A total of 3,280 unigenes were assigned to GO, 3,388 unigenes were classified into Cluster of Orthologous Groups of proteins (COG) using BLAST and BLAST2GO software, and 2,568 were mapped onto pathways using the Kyoto Encyclopedia of Gene and Genomes Pathway database. Furthermore, 218 unigenes were deemed to be DEGs. After enrofloxacin treatment, 135 genes were upregulated and 83 genes were downregulated. The GO terms biological process (126 genes) and metabolic process (136 genes) were the most enriched, and the terms for protein folding, response to stress, and SOS response were also significantly enriched.

This study identified enrofloxacin treatment affects multiple biological functions of *A*. *hydrophila*. Enrofloxacin resistance in *A*. *hydrophila* is closely related to the reduction of intracellular drug accumulation caused by ABC transporters and increased expression of topoisomerase IV.

## Introduction

Following the decline in the capture fishing industry and diminishing wild fish stocks, the aquaculture industry has become an important source of food fish [1]. However, bacterial diseases hinder desirable production outputs. The gram-negative bacterium *Aeromonas hydrophila* is one of the major causative agents of disease and can cause serious damage in many animals [2], especially fish [3–4] as well as humans [5]. *A*. *hydrophila*, which is a representative of the Aeromonadaceae family, is an emerging aquatic pathogen that is distributed in a wide variety of aquatic systems [6–7]. It primarily inhabits freshwater and the intestines of freshwater animals. Farmers use a wide range of antibiotics or chemicals to control *A*. *hydrophila* infection [8]. Enrofloxacin is a third-generation fluoroquinolone with a broad antibacterial spectrum and high potency that is commonly used to treat bacterial infections afflicting aquaculture [9]. Enrofloxacin has been used primarily to control *A*. *hydrophila* infections in aquaculture, and recently, *A*. *hydrophila* has developed strong resistance to enrofloxacin among other drugs [10]. This resistance has rendered it increasingly difficult to treat diseases caused by *A*. *hydrophila* in aquaculture animals. Moreover, heavy antibiotic use is associated with negative effects such as antibiotic resistance in the environment and fish [11]. Thus, eventually, antibiotic use may be detrimental to the environment and human health.

Based on previous reports, quinolone-resistant bacteria adopt the following three main strategies of antibiotic resistance: First, chromosome-mediated changes in the topoisomerase target sites (changes in the amino acids in the quinolone resistance-determining region) [12]; second, reduction in intracellular drug accumulation caused by efflux pump [13]; and third, bacterial protection conferred by plasmid-encoded qnr protein [14]. However, the mechanisms by which *A*. *hydrophila* is resistant to enrofloxacin remains unclear, and little is known about its molecular mechanisms of resistance. Here, we conducted *de novo* transcriptome sequencing for the comprehensive analysis of the global transcriptomes of enrofloxacin-susceptible and enrofloxacin-resistant strains. Transcriptomic profiling is used to analyze gene expression and signaling pathways in specific tissues or cells. The recent rapid development of next-generation sequencing technologies such as the Solexa/Illumina technology offers great advantages in analyzing the functional complexity of the entire transcriptome [15]. Next-generation sequencing techniques have been used for transcriptome analyses to simultaneously provide data on sequence polymorphisms and the levels of gene expression involved in cellular development, cancer, and immune responses [16–17].

In the present study, we examined the genetic diversity of *A*. *hydrophila* using *de novo* transcriptome sequencing and investigated the molecular mechanisms of enrofloxacin resistance in *A*. *hydrophila*.

## Materials and methods

### Culture of susceptible strain and induction of an enrofloxacin-resistant strain

*A*. *hydrophila* ATCC 7966, which was used in the present study, had been maintained at the National Pathogen Collection Center for Aquatic Animals, China. Because the genome background of the ATCC 7966 standard strain is known, it is known to be sensitive to quinolones; therefore, it was selected as the sensitive strain. This strain was inoculated on Luria Broth (LB) agar and incubated at 28°C for 24 h.

The type strain ATCC 7966 was used to develop an enrofloxacin-resistant strain by culturing it in the presence of gradually increasing concentrations of enrofloxacin in vitro, and enrofloxacin-resistant strain 7966QR was judged by the critical concentration suggested by the Clinical and Laboratory Standards Institute (CLSI, 2011). The results of the drug sensitivity test were determined using the disc diffusion method. Resistance and sensitivity to enrofloxacin were determined on the basis of the drug sensitivity evaluation criteria issued by the American Association of Clinical Laboratory Standards in 2011 [18]. Briefly, ATCC 7966 cells were inoculated on LB agar containing 1/2 the minimum inhibitory concentration (MIC) of enrofloxacin (Shanghai Guoyao Chemical Reagent Co. Shanghai, China). Every 2 days, the cultured strains were inoculated into fresh LB agar containing 2× the previous concentration of enrofloxacin. The strains were inoculated to LB agar that containing no enrofloxacin until the MIC was conformed to the drug resistance determination. The resultant resistant strains were screened on LB for 12 generations until the resultant strain (7966QR) could be deemed resistant.

Suspensions containing the susceptible (ATCC 7966) and resistant (7966QR) strains were collected and centrifuged, and the final products were store at 4°C until analysis.

### RNA isolation, RNA-Seq library construction, and sequencing

Frozen samples were ground in a mortar with liquid nitrogen, and total RNA was extracted from approximately 1 mL of bacterial suspension with TRIzol reagent (Invitrogen, USA), according to the manufacturer’s instructions. DNA contaminants were removed by treatment with RNase-free DNase I (Takara Biotechnology, Dalian, China). The resultant total RNA was dissolved in 200 μL RNase-free water. The concentration of total RNA was determined using a Nano-Drop2000 spectrophotometer (Thermo Scientific, USA), and its integrity was checked using an RNA 6000 Pico LabChip with the Agilent 2100 bioanalyzer (Agilent, USA) at 37°C for 1 h, and the sample volume was diluted to 250 μL using nuclease-free water. Messenger RNA (mRNA) was further purified with a Micropoly (A) Purist kit (Ambion, USA) according to the manufacturer’s protocol. mRNA was dissolved in 100 μL RNA Storage Solution (Ambion), purified using oligo (dT) magnetic beads, fragmented by treatment with divalent cations and heat, and reverse transcribed into cDNA using reverse transcriptase and random hexamer primers. This was followed by second-strand cDNA synthesis using DNA polymerase I and RNaseH. The resultant double-stranded cDNA was end-repaired using T4 DNA polymerase, Klenow fragments, and T4 polynucleotide kinase followed by a single (A) base addition using Klenow 3′ to 5′ exopolymerase. This was then ligated with an adapter or index adapter using T4 Quick DNA ligase. The size range of the adapter-modified fragments was selected by gel purification, and these sizes were used as templates in PCR amplification. The cDNA library was validated with an Agilent 2100 Bioanalyzer and ABI StepOnePlus Real-time PCR system and sequenced on a flow cell using an Illumina HiSeq 2500 (Illumina, San Diego, CA, USA).

### Sequencing, data processing, and quality control

We filtered low-quality reads and removed 3′-adapter sequences using Trim Galore. The obtained reads were cleaned using FastQC software (http://www.bioinformatics.babraham.ac.uk/projects/fastqc/), and the content and quality of the nucleotide bases in the sequencing data were evaluated. Next, we conducted a comparative analysis with the reference genome (Aeromonas hydrophila subsp. Hydrophila ATCC 7966). For each sample, sequence alignment with the reference genome sequences was carried out using Tophat [19].

### Assembly and functional annotation

High-quality reads were obtained after removing the adapter sequence, low-quality reads (reads with ambiguous bases N), and duplicate sequences using Trim Galore and FastQC, and then FastQC software was used to clean reads and evaluate the performance of different k-mers. Next, the clean reads were combined using de Bruijn graphs and SOAPdenovo software based on sequence overlap to form longer fragments (without ambiguous ‘N’ reads), to create contigs [20]. Furthermore, the contigs were connected into transcript sequences and joined into scaffolds using paired-end reads. The paired-end reads were also used to fill the gaps in scaffolds, where the unigenes have the least Ns and cannot be extended on both ends. Based on the results of the assembly evaluation, the best results were selected and used for clustering analysis using TGI Clustering tools to achieve a unigene database [21]. The obtained unigenes were compared with the National Center for Biotechnology Information (NCBI), non-redundant protein (Nr), and UniProt databases using BLASTx (Basic Local Alignment Search Tool) search with an E value < 0.00001. Based on the results of the Nr annotation, we used Blast2GO software (https://www.blast2go.com/) to analyze functional annotations by gene ontology terms (GO; http://www.geneontology.org) [22]. The unigenes were also aligned to the Kyoto Encyclopedia of Genes and Genomes (KEGG), Clusters of Orthologous Group (COG), and Swiss-Prot databases to predict and classify gene functions to perform pathway annotation searching for unigenes with similarity >30% and an E value <0.00001, and all the information was merged.

### Analysis of differentially expressed unigenes

To estimate the expression level (relative abundance) of a specific transcript expressed as fragments per kilobase per million fragments mapped (FPKM), we used RSEM software with the default parameter settings [23]. The expression level of each transcript was transformed using base 2 log_2_(FPKM+1). The fold changes in the expression of a transcript and differentially expressed gene (DEG) were estimated using DESeq software [24]. Two-fold changes in expression level and differences with a p value of <0.05 were considered significant.

### GO functional and pathway enrichment analysis of DEGs

We annotated DEGs to analyze the transcriptome differences between enrofloxacin-resistant and enrofloxacin-susceptible strains of *A*. *hydrophila*. To this end, we used GO terms in accordance with previously published procedures [25]. This p = analysis first mapped all DEGs to GO terms in the database by calculating gene numbers for every term followed by an ultra-geometric test to identify significantly enriched GO terms in DEGs compared to the transcriptome background. The following formula was used:

$$p=1-\sum_{i=0}^{m-1}\frac{\left(\begin{array}{c} M\\ i \end{array})(\begin{array}{c} N-M\\ n-i \end{array}\right)}{\left(\begin{array}{c} N\\ n \end{array}\right)}$$

where N represents the number of all genes with GO annotation, n represents the number of DEGs in N, M represents the number of all genes annotated to specific GO terms, and m represents the number of DEGs in M. The calculated p value was subjected to Bonferroni correction. A corrected p value <0.05 was defined as the “threshold.” GO terms were considered significantly enriched in the DEGs.

### Pathway analysis of DEGs

Pathways of DEGs were annotated against the KEGG database using the BLASTall program (http://nebc.nox.ac.uk/bioinformatics/docs/blastall.html). Enriched DEG pathways were identified according to the same formula as that used in the GO analysis. In this case, N represented the number of all genes with KEGG annotations, n represented the number of DEGs in N, M was the number of all genes annotated to specific pathways, and m was the number of DEGs in M [25].

### Verification of DEGs using qRT-PCR

Quantitative RT-PCR (qRT-PCR) was used to verify the expression levels of DEGs that were identified by RNA-Seq analysis. Primers were designed using Primer 5 software and SpTub-b was used as the reference gene [25,26]. Reactions were performed in a 25-μL reaction volume composed of 2 μL cDNA, 0.5 μL each of forward and reverse primers (10 μM), 12.5 μL SYBR Premix Ex Taq (2×), and 9.5 μL RNase-free H_2_O. The thermal cycle protocol was as follows: 95°C for 30 s followed by 40 cycles of 95°C for 5 s, 60°C for 30 s, and 72°C for 30 s. Melting curve analysis was performed at the end of qRT-PCR to confirm PCR specificity.

## Results

### Illumina sequencing and quality assessment

Differences in gene expression between the enrofloxacin-susceptible and enrofloxacin-resistant strains of *A*. *hydrophila* were determined by sequencing the RNA-Seq data using the Illumina sequencing platform. After filtering and quality checks of the raw reads (26,316,850 and 26,910,746 reads for the 7966QR and ATCC 7966 strains, respectively), approximately 26 million (26,123,674) and 26 million (26,730,263) trimmed reads with trim rates of 99.27% and 99.33% were obtained for 7966QR and ATCC 7966, respectively. Meanwhile, the average lengths of reads for these two strains were 119.66 and 120.61 bp, and their GC percentages were 55% and 54%, respectively (Table 1), indicating successful sequencing of the *A*. *hydrophila* transcriptome. Trimmed reads were used for the subsequent analysis.

**Table 1:** Summary of reads in *A*. *hydrophila* transcriptome sequencing.

Sample	Raw reads	Trimmed reads	Average length	Trim rate	GC rate
ATCC 7966	26,316,850	26,123,676	119.66bp	99.27%	55%
7966QR	26,910,746	26,730,263	120.61bp	99.33%	54%

### Comparative analysis with reference genome

The trimmed reads of the *A*. *hydrophila* transcriptome were compared with the reference genome sequence. The total mapping rates of the reads with the reference genome were 94.19% and 93.29% in the ATCC 7966 and 7966QR groups, respectively. There were approximately 22 million (22,717,810) and 21 million (21,997,467) uniquely mapped reads for the ATCC 7966 and 7966QR groups, accounting for 85.51% and 84.78% of the total reads, respectively. There were approximately 2,306,148 and 2,208,047 multiple mapped reads in the ATCC 7966 and 7966QR groups, respectively, accounting for 8.68% and 8.51% of the total reads, respectively. The number of reads mapped in proper pairs accounted for 84.04% and 83.31% in the ATCC 7966 and 7966QR groups, respectively (Table 2).

**Table 2:** Statistical results of trimmed reads mapping with reference genome.

Map to genome	7966QR		ATCC 7966	
	Read numbers	Percentage	Read numbers	Percentage
Total reads	25,946,162	100.00%	26,566,428	100.00%
Total mapped	24,205,514	93.29%	25,023,958	94.19%
Uniquely mapped	21,997,467	84.78%	22,717,810	85.51%
Multiple mapped	2,208,047	8.51%	2,306,148	8.68%
Reads1 mapped	11,003,361	42.41%	11,370,421	42.80%
Reads2 mapped	10,994,106	42,37%	11,347,389	42.71%
Mapped to ‘+’	11,004,476	42.41%	11,368,564	42,79%
Mapped to ‘-’	10,992,991	42.37%	11,349,246	42,72%
Reads mapped in proper pairs	21,614,858	83.31%	22,327,558	84,04%

**Fig 1:**
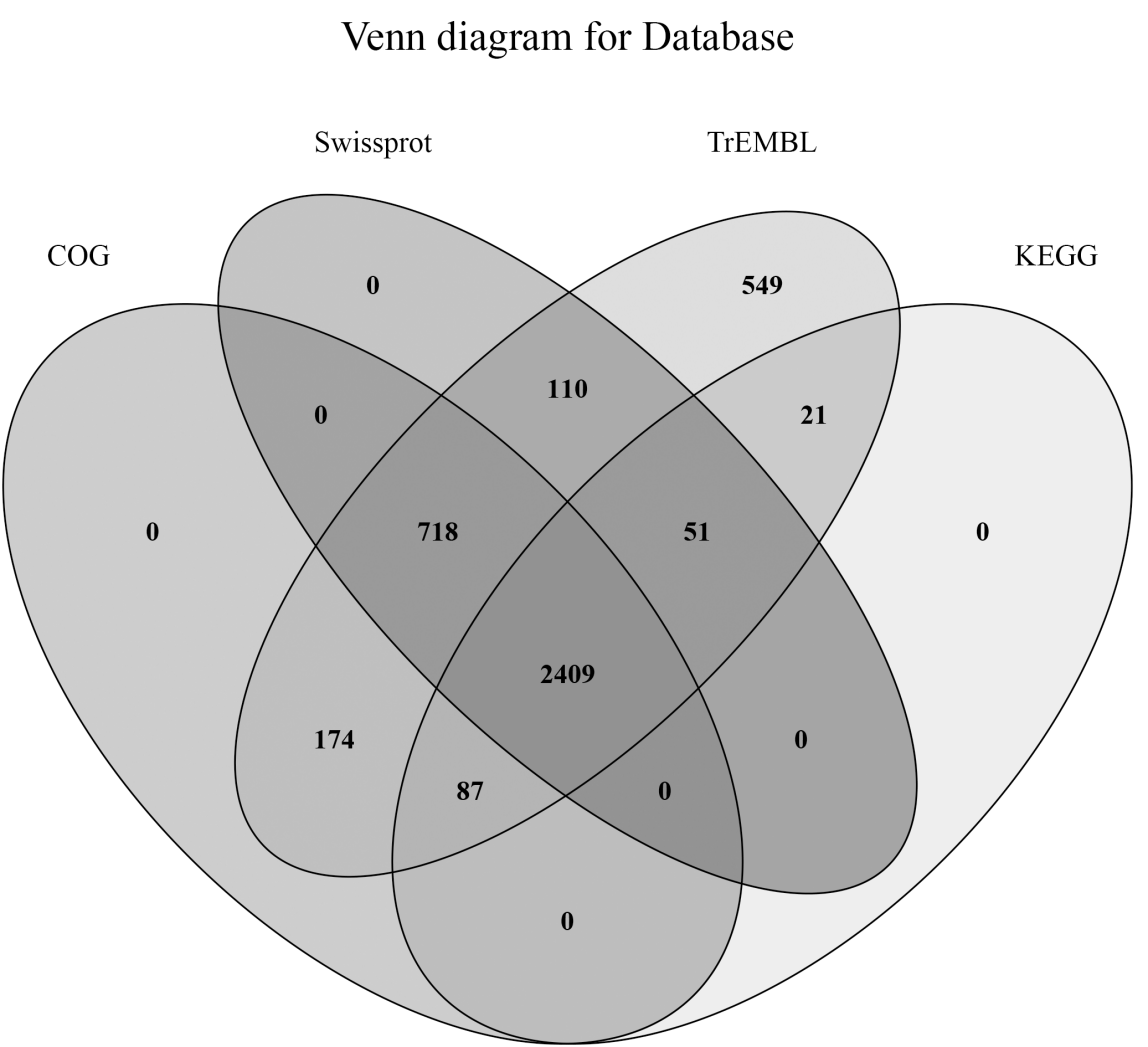
Venn diagram representation of database annotations.

We then compared the unigenes of the sample species with the common data genes, and functional annotation was performed based on the similarity of the genes. The protein sequences were compared with the KOG, GO, and KEGG databases. The annotation of unigenes in the Swiss-Prot and TrEMBL databases accounted for 79.77% and 99.93% of the total unigenes, respectively (Table 3 and Fig 1).

**Table 3:** Statistical results of the gene functional annotation.

Database	Number of unigenes	Percentage (%)
Annotation in COG	3388	82.19
Annotation in Swiss-Prot	3288	79.77
Annotation in TrEMBL	4119	99.93
Annotation in GO	3280	79.57
Annotation in KEGG	2568	62.3
Annotation in at least one database	4119	99.93
Annotation in all databases	2313	56.11
Total Unigenes	4122	100

The unigene annotations in the COG, GO, and KEGG databases were about 82.19%, 79.57%, and 62.3%, respectively (Table 3). Transcripts were analyzed by COG classification. There were 3,388 unigenes clustered into 25 functional categories (S1 Fig). The “amino acid transport and metabolism” and “signal transduction mechanisms” clusters represented the majority of transcripts (276 transcripts, 8.15%; S1 Fig). GO and KEGG database analysis of unigenes revealed that most unigenes were enriched in cellular processes, environmental information processing, genetic information processing, metabolism, and organismal systems (S2 and S3 Figs).

### Analysis of DEGs

**Fig 2:**
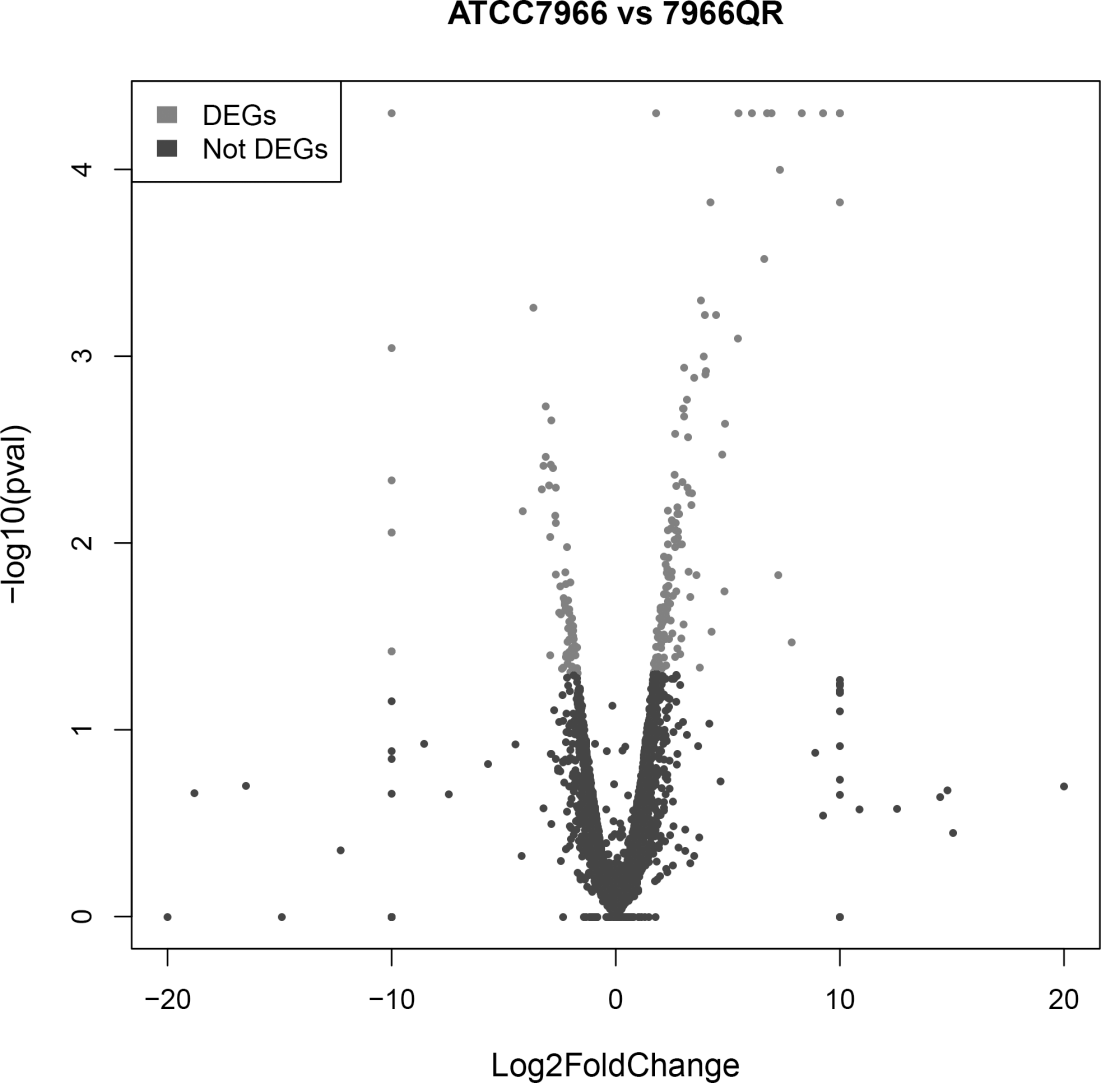
Effect of enrofloxacin treatment on the gene expression profile in resistant and susceptible strains of *A*. *hydrophila*. Volcanic plot of the degree of differences between the expression profiles of resistant and susceptible *A*. *hydrophila* strains. X-axis, log_2_(fold change); Y-axis, -log_2_(P value). Gray, differential expression genes; black, not differential expression genes. Each dot represents one gene.

**Fig 3:**
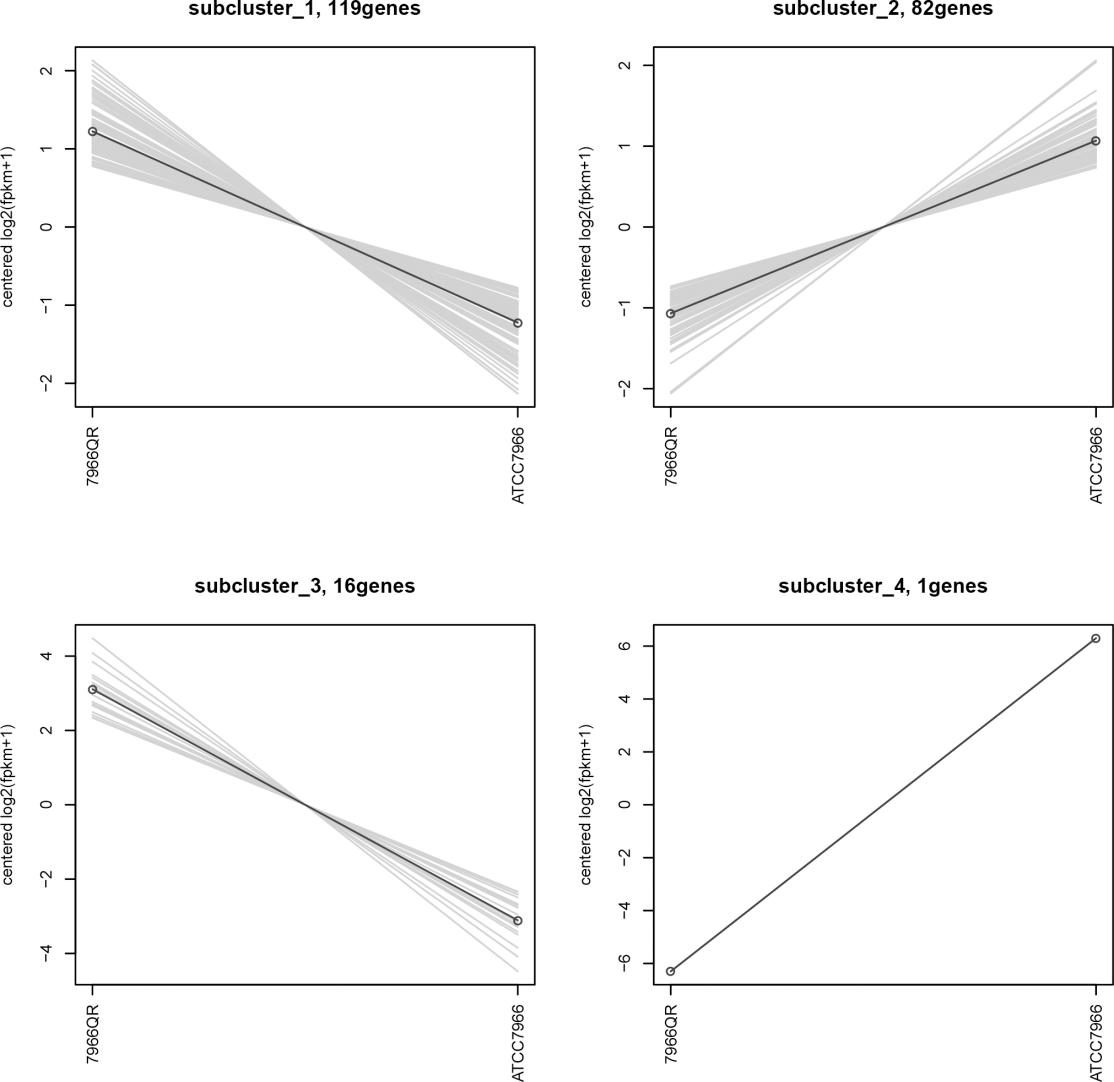
Heatmap representation of DEGs between resistant and susceptible strains of *A*. *Hydrophila*. A broken line in the figure represents a gene's expression in different samples. The graph shows that all the genes under each cluster are similar in all samples.

The Cuffdiff program was used to generate *A*. *hydrophila* gene expression profiles to identify genes that are differentially expressed between the resistant and susceptible strains of *A*. *hydrophila* (Figs 2 and 3). The program identified that among the DEGs, 135 genes were markedly upregulated and 83 were markedly downregulated, indicating that the gene expression had changed in the drug-resistant strains.

### GO annotation of DEGs

We used GO to determine the biological functions in which the DEGs are involved. GO functional enrichment analysis also involved cluster analysis of expression patterns. Thus, the expression patterns of DEGs annotated with a given GO term were easily obtained. All the annotated genes were classified into three GO domains: biological process, cellular component, and molecular function. Dissimilar expression profiles of the DEGs in the treated and control groups were used to determine the molecular mechanisms of enrofloxacin resistance in *A*. *hydrophila*.

**Fig 4:**
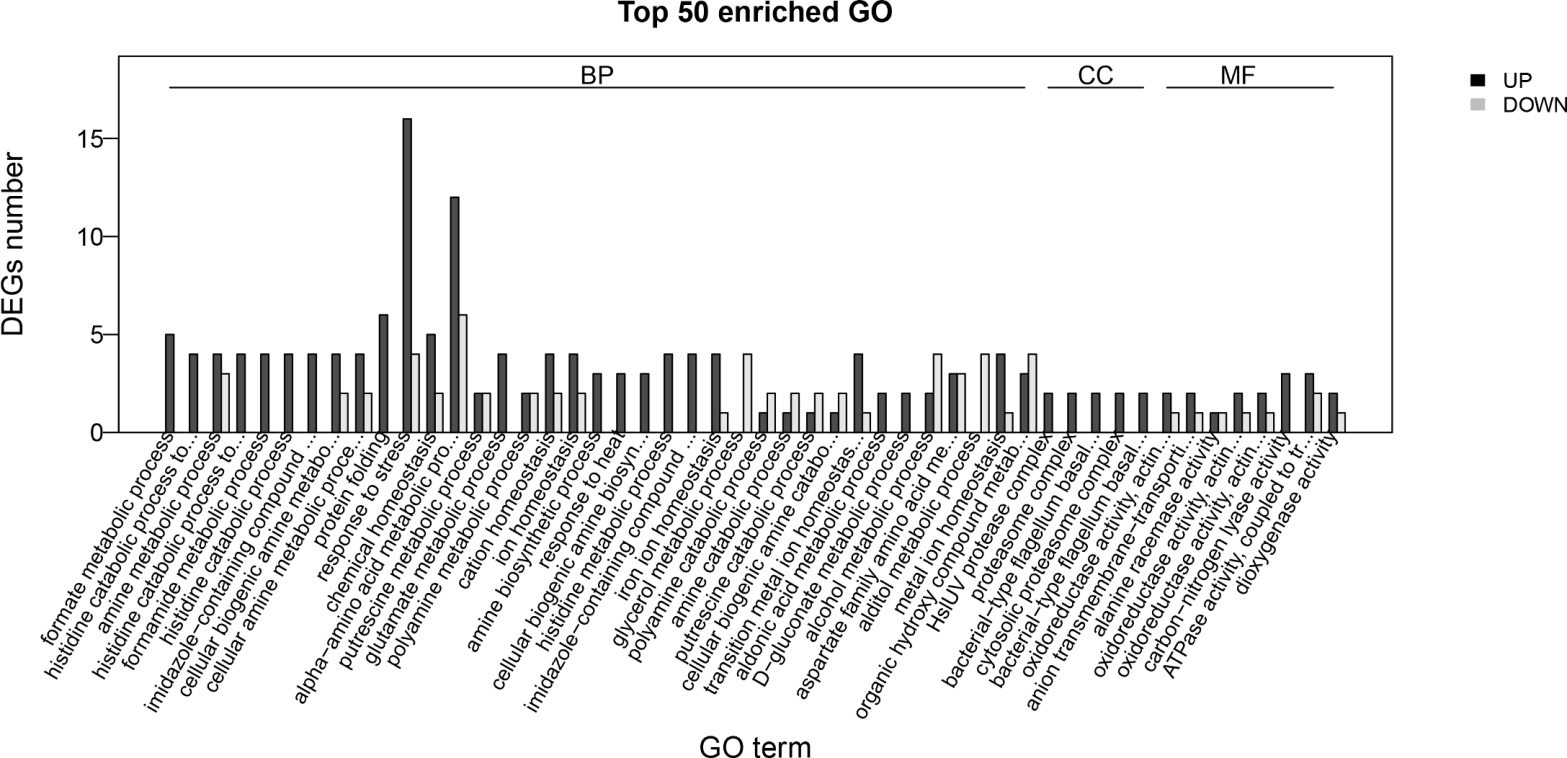
Histogram representation of enriched terms from GO annotation of DEGs in resistant versus susceptible strains of *A*. *hydrophila*. GO terms (X-axis) were grouped into three main ontologies: biological process, cellular component, and molecular function. The Y-axis indicates the number of DEGs. All annotated DEGs were classified into three GO domains: biological process, cellular component, and molecular function.

The expression profiles of the three GO domains were as follows (Fig 4 and S1 Table).

Biological process: formate metabolic process (5 genes), histidine catabolic process to glutamate and formate (4 genes), amine metabolic process (7 genes), histidine catabolic process to glutamate and formamide (4 genes), formamide metabolic process (4 genes), histidine catabolic process (4 genes). Cellular component: HslUV protease complex (2 genes), proteasome complex (2 genes), bacterial-type flagellum basal body, distal rod (2 genes), cytosolic proteasome complex (2 genes). Molecular function: oxidoreductase activity, acting on paired donors, with incorporation or reduction of molecular oxygen (3 genes); anion transmembrane-transporting ATPase activity (3 genes); oxidoreductase activity, acting on single donors with incorporation of molecular oxygen (3 genes); oxidoreductase activity, acting on single donors with incorporation of molecular oxygen, incorporation (3 genes); carbon-nitrogen lyase activity (3 genes); ATPase activity, coupled to transmembrane movement of ions (5 genes); and dioxygenase activity (3 genes).

**Fig 5:**
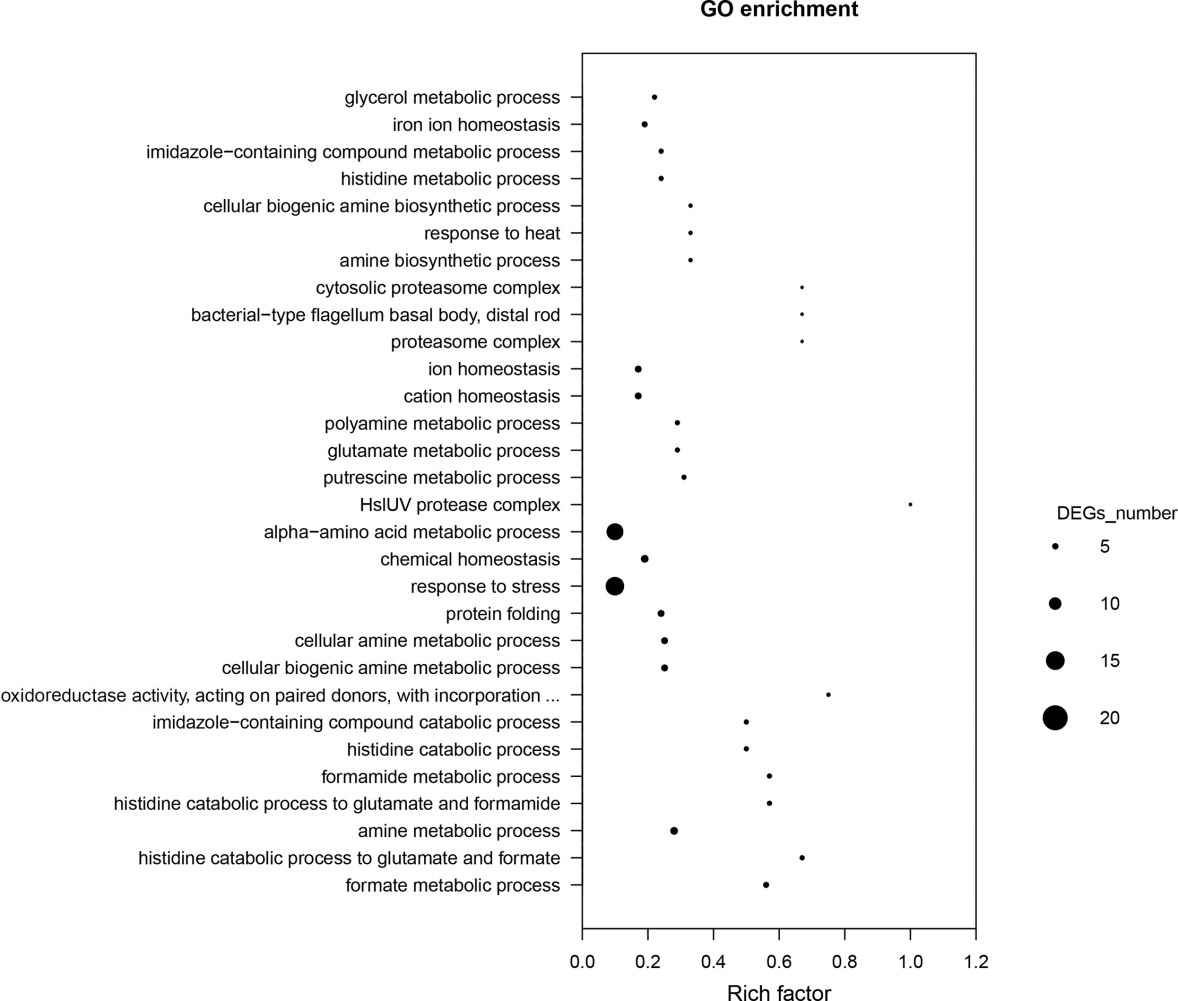
Scatter plot of the enriched GO annotation of differential expression genes (DEGs) in resistant and susceptible strains of *A*. *hydrophila*. Scatter plot of the degree of differences in the expression profile of *E*. *sinensis*. X-axis, Rich factor; Y-axis, pathway name. A corrected p-value < 0.05 was defined as ‘threshold’. GO terms were considered significantly enriched in the DEGs. The size of the dots indicates the number of DEGs contained in each term.

The following DEGs related to the biological process were upregulated: AHA_0377 (formate metabolic process); AHA_0377; AHA_0378 (histidine catabolic process to glutamate and formate); AHA_0377; AHA_0378; AHA_0379; AHA_0380 (glutamate metabolic process); and cellular component relate genes such as AHA_4114, AHA_4115 (HslUV protease complex), AHA_4114, AHA_4115 (cytosolic proteasome complex), AHA_1948, AHA_3601 (oxidoreductase activity), AHA_0380, AHA_1413, and AHA_4201 (carbon-nitrogen lyase activity). The biological process-related genes such as AHA_1213, AHA_1652 (glycerol metabolic process), AHA_4006 (alditol metabolic process), AHA_1921, and AHA_2046 (endoplasmic reticulum) were downregulated. The functions of the hypothetical proteins were not clear, but were closely related to the biological functions of organisms. (Fig 5)

Overall, the above analyses shed insight into the molecular mechanisms of enrofloxacin resistance in *A*. *hydrophila*.

### KEGG pathway analysis of DEGs

**Fig 6:**
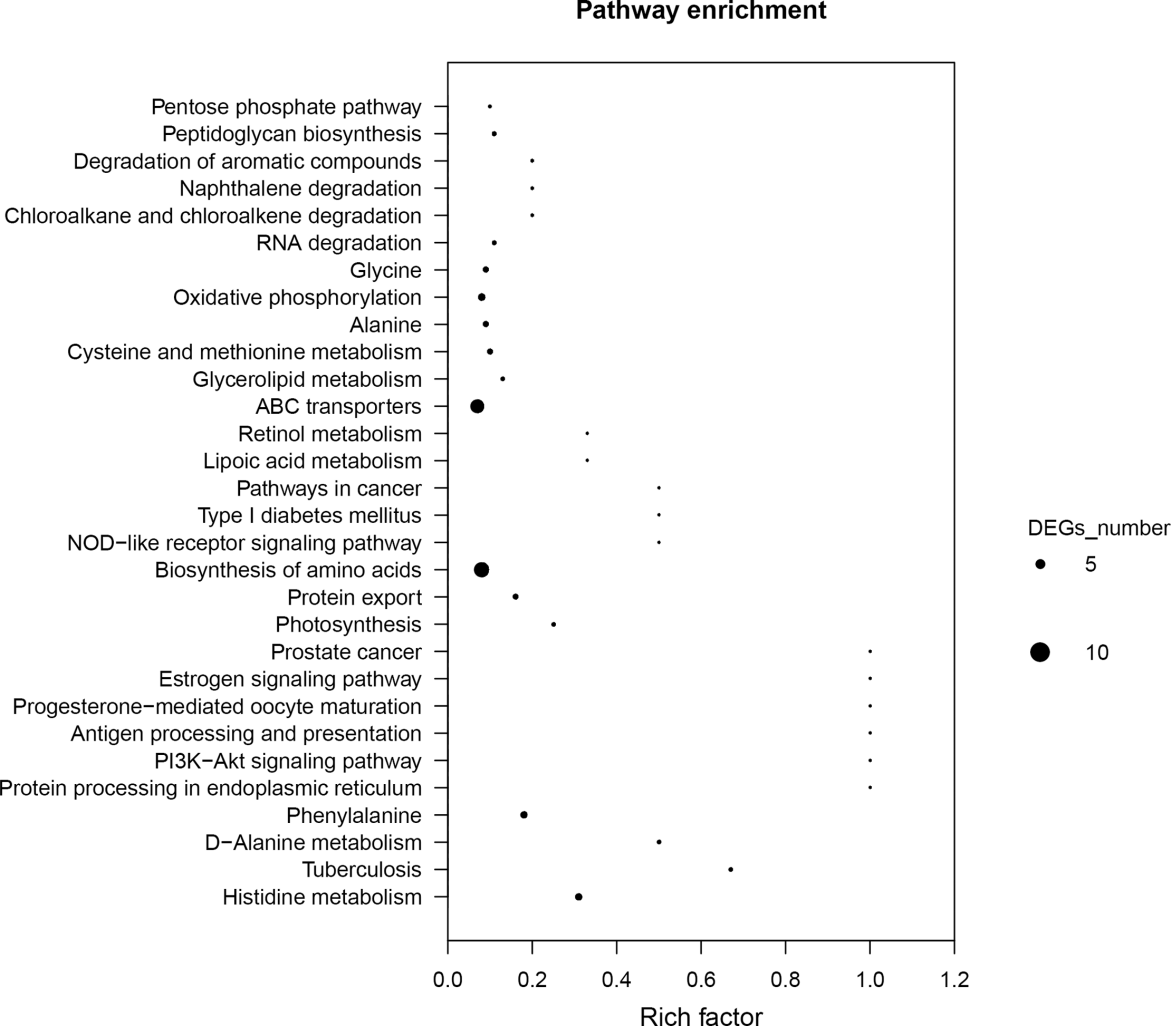
Scatter plot of enriched pathways from KEGG annotation of differential expression genes (DEGs) in resistant versus susceptible strains of *A*. *hydrophila*. Scatter plot of the degree of differences in the expression profile of *E*. *sinensis*. X-axis, Rich factor; Y-axis, pathway name. A corrected p-value < 0.05 was defined as ‘threshold’. KEGG pathway were considered significantly enriched in the DEGs. The size of the dots to indicate the number of DEGs contained in each pathway.

To further explore the biological functions of the DEGs, DEGs were mapped to the KEGG database and enriched to important pathways based on the whole transcriptome. A total of 218 genes were mapped to 67 pathways. Many of the genes were found in multiple pathways, whereas many others were restricted to a single pathway. These pathways included metabolism, genetic information processing, cellular processes, organismal systems, and environmental information processing. The metabolism-related pathways were the most significantly (Fig 6 and S2 Table).

The metabolism-related biological pathways included metabolism of amino acids and their important derivatives, drug metabolism pathway, and carbohydrate metabolism. A total of 86 genes were mapped to metabolism-related biological pathways. In addition, lipid metabolism-related biological pathways, including fatty acid metabolism (3 genes), glycerolipid metabolism (2 genes), and glycerophospholipid metabolism (2 genes), were also significantly enriched. Enriched amino acid metabolism-related biological pathways included histidine metabolism (4 genes), d-alanine metabolism (2 genes), phenylalanine (4 genes), cysteine and methionine metabolism (2 genes), alanine metabolism (3 genes), glycine metabolism (3 genes), tyrosine metabolism (1 gene), lysine biosynthesis (1 gene), and arginine and proline metabolism (2 genes). Metabolism of xenobiotics by cytochrome P450 (1 gene) and drug metabolism-cytochrome P450 (1 gene) were the drug metabolism pathways that had been enriched. The carbohydrate metabolism pathways that were enriched included pentose phosphate pathway (2 genes), fructose and mannose metabolism (2 genes), glycolysis/gluconeogenesis (2 genes) (S2 Table).

The other significantly enriched pathways included environmental information processing (19 genes), cellular processes (3 genes), genetic information processing (9 genes), and organismal systems (5 genes). The environmental information processing pathways that were enriched included PI3K-Akt signaling pathway (1 gene), ABC transporters (9 genes), bacterial secretion system (3 genes), phosphotransferase system (PTS) (1 gene). Enriched cellular processes included flagellar assembly (2 genes) and bacterial chemotaxis (1 gene). Enriched genetic information processing included protein processing in endoplasmic reticulum (1 gene), protein export (3 genes), RNA degradation (2 genes). Additionally, the following pathways were enriched: antigen processing and presentation (1 gene), progesterone-mediated oocyte maturation (1 gene), estrogen signaling pathway (1 gene), NOD-like receptor signaling pathway (1 gene), and plant-pathogen interaction (1 gene) (S2 Table).

The expression of certain genes such as AHA_2490 (PI3K-Akt signaling pathway); AHA_2490 (NOD-like receptor signaling pathway); AHA_0608, AHA_2812, AHA_0913, AHA_3728, AHA_1964, AHA_1687, AHA_4285, AHA_1595, and AHA_2813 (ABC transporters) were upregulated, while the AHA_1419 (environmental information processing), AHA_1331 (drug metabolism-cytochrome P450), AHA_2360, AHA_1331 (glycolysis/gluconeogenesis), and AHA_1331 (metabolism of xenobiotics by cytochrome P450) genes were downregulated. The ABC transporter genes were expressed at higher levels than drug metabolism-cytochrome P450, indicating that *A*. *hydrophila* resistance to enrofloxacin may be mediated by a mechanism involving ABC transporters. These finding are consistent with the results of the GO enrichment analysis.

Overall, the results of the DEG pathway analysis support the viewpoint that enrofloxacin inhibits *A*. *hydrophila* growth by affecting multiple biological functions, such as energy biogenesis, protein synthesis, and metabolism.

### Verification of the differential expression of DEGs

Based on the results of the GO and KEGG analyses, the primers of eight genes significantly differed between the resistant and susceptible *A*. *hydrophila* strains and were therefore considered to be related to drug metabolism. With clear functional implication, these primers were designed to verify the expression of these DEGs identified in the RNA-Seq analysis. All primer sequences are listed in Table 4.

**Table 4:** Oligonucleotide primers of qRT-PCR for validation of DEGs.

Gene name	Putative function	GO category	Pathway name	Primer name	Nucleotide sequence (5′-3′)	Expected product
ACTIN	-	-	-	ACTIN-F	TGTGTAGCGGTGAAATGCG	140bp
				ACTIN-R	CATCGTTTACGGCGTGGAC	
METL	Aspartokinase II	Aspartate family amino acid biosynthetic process (Biological process)	Metabolic pathways	METL-F	AAGGTGTAGTTGCTGGAGAGGT	130bp
				METL-R	GCGTGTGAAGAGACATCAAGGA	
METE	5-methyltetrahydropteroyltriglutamate	Methylation (Biological process)	Metabolic pathways	METE-F	CTTACGAGGCGGGCATTCAG	151bp
				METE-R	AAGCGGGTGATGGCAAAGC	
air-2	alanine racemase	regulation of cell shape, peptidoglycan biosynthetic process (Biological process)	Metabolic pathways	air-2-F	AACGCTTTCTCTGGCTCCCTA	125bp
				air-2-R	CGACATCAGCACGGCATTCA	
air	alanine racemase	peptidoglycan biosynthetic process, alanine metabolic process (Biological process)	Metabolic pathways	air-F	ACCGCACCTTCACCCTCAA	209bp
				air-R	GAACAGCACCACCTCGTCAC	
AHA2142	Acetyl-CoA acetyltransferase	signal transduction, metabolic process, cholesterol metabolic process (Biological process)	Metabolic pathways	AHA2142-F	GGAGACATTGCCGAAGTGACC	118bp
				AHA2142-R	CTACCTCATAGTGCCGCTCAAC	
gyrB1	DNA gyrase subunit B	DNA topoisomerase type II (ATP-hydrolyzing) activity, ATP binding, metal ion binding, DNA replication origin binding, GTPase activity (Molecular process)	Metabolic pathways	gyrB1-F	GCGGAATGTTGTTGGTGAAGC	173bp
				gyrB1-R	CTACGAAGGCGGCATCAAGG	
gyrA	DNA gyrase subunit A	DNA topoisomerase type II (ATP-hydrolyzing) activity, magnesium ion binding, protein heterodimerization activity (Molecular process)	Metabolic pathways	gyrA-F	GTCTTCTCGTCCACCTCCACT	222bp
				gyrA-R	CAACATTCCGCCTCACAACCT	

**Fig 7:**
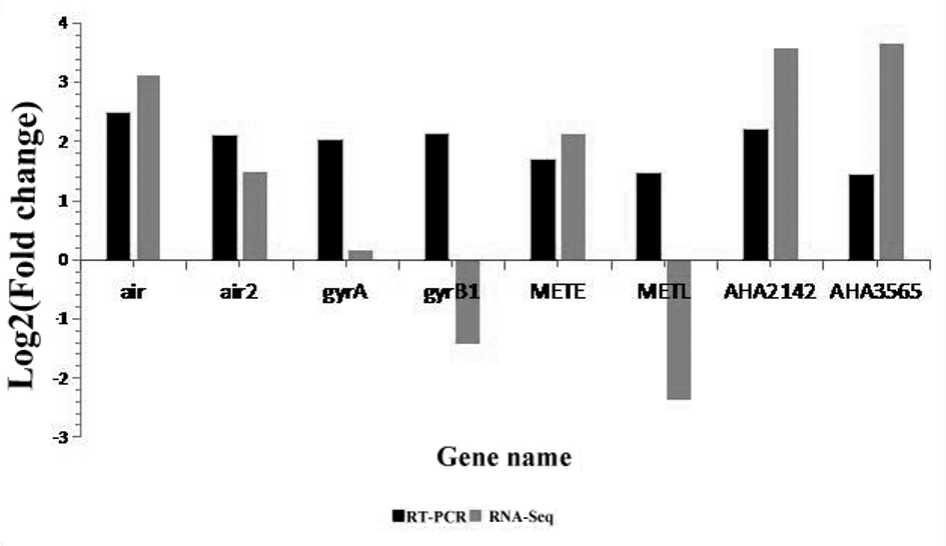
Comparison of the expression levels of eight genes determined by RNA-Seq and RT-PCR. Negative values indicate that gene expression in *A*. *hydrophila* was downregulated following enrofloxacin treatment; positive values indicate that the gene expression was upregulated.

The data revealed that the upregulation or downregulation of these six genes was consistent with the RNA-Seq results. Together, these results indicate that the qRT-PCR and RNA-Seq results were reliable overall; however, further studies to determine the molecular mechanisms of resistance to enrofloxacin are required (Fig 7).

## Discussion

Transcriptome sequencing is a powerful technique for studying the mechanisms of changes in biological characteristics of an organism and has been used successfully in some species [27–29]. In our study, we examined the transcriptome of *A*. *hydrophila* using the Illumina sequencing platform and explored the molecular mechanism of enrofloxacin resistance in *A*. *hydrophilia*. Compared with the reference genome, the total mapped rates of reads were 94.19% and 93.29% in the *A*. *hydrophilia* transcriptome of the enrofloxacin-susceptible (ATCC 7966) and enrofloxacin-resistant (ATCC 7966QR) strains, respectively, indicating that the quality of sequencing data met the demand for follow-up studies.

We obtained 218 DEGs and classified them into 1,052 GO terms consisting of three domains: biological process, cellular component, and molecular function. Of these, 176 GO terms were found to have dramatic changes in expression. We mapped the DEGs to 68 pathways, of which 10 were significantly enriched. We divided the genes into five branches based on the KEGG metabolic pathway involved: cellular processes, environmental information processing, genetic information processing, metabolism, and organismal systems. The metabolism-related biological pathways and biosynthesis of amino acids were the most significantly enriched pathways in this analysis; these pathways are responsible for the main biological functions of *A*. *hydrophila*. The results of the KEGG pathway analysis revealed that a considerable percentage of genes was enriched in ABC transporters, metabolism of xenobiotics by cytochrome P450, and drug metabolism-cytochrome P450. All genes enriched in ABC transporters were upregulated, whereas all the genes enriched in metabolism of xenobiotics by cytochrome P450 and drug metabolism-cytochrome P450 were downregulated. We speculate that enrofloxacin promotes the protein expression of ABC transporters and inhibits the protein expression of cytochrome P450. This may be related to enrofloxacin resistance of *A*. *hydrophila*. Our study supplements the previous studies by Seshadri et al. who investigated the virulence genes in *A*. *hydrophila* by sequencing its genome [30]. The results of the GO annotation enrichment revealed that most DEGs were related to transmembrane transport and biosynthesis and degradation of amino acids. Furthermore, some of the genes were mapped to cellular response to DNA damage stimulus, and among the DEGs, *gyrA* was upregulated, which was verified by qRT-PCR. The qRT-PCR results were generally consistent with the results of the transcriptome analysis. Our results are in agreement with the results of Shakir et al., who reported that changes in the topoisomerase target sites of chromosomes (amino acid changes in quinolone resistance-determining regions, QRDRs) could produce drug resistance and, *A*. *hydrophila* strains exhibiting high levels of resistance to antibiotics are common in the strains with *gyrA* and *parD* double mutations in QRDRs [5]. Together, these results indicate that *A*. *hydrophilia* resistance to enrofloxacin occurs primarily due to alterations in multiple biological functions, energy biogenesis, protein synthesis, and metabolism. Our findings support that the mechanism of enrofloxacin resistance in *A*. *hydrophila* is closely related to reduction of intracellular drug accumulation caused by ABC transporters and topoisomerase IV.

Most of the genes in *A*. *hydrophilia* encode putative proteins whose functions are not clear. In order to facilitate further research, we selected 8 of the DGEs whose functions were clearly known and subjected them to qPCR analysis to verify the RNA-Seq results of. The qPCR results were consistent with the results of the transcriptome analysis, except for two genes. Therefore, we believe that the qualities of the *A*. *hydrophila* transcriptomes are adequate for further studies on its functional genes. These findings greatly extend the existing sequence resources relating to *A*. *hydrophilia* and provide abundant genetic information that can be applied to further understand the molecular mechanisms of enrofloxacin resistance in *A*. *hydrophilia* in aquaculture.

## Supporting information

S1 Table GO analysis of differentially expressed genes of *Aeromonas hydrophil*a. (DOC)

S2 Table KEGG pathway analysis of differentially expressed genes of *Aeromonas hydrophila*. (DOCX)

S1 Fig Cluster of orthologous group (COG) classification. (TIF)

S2 Fig Histogram representation of the enriched category arising from the GO annotation of unigenes in *A*. *Hydrophila*. Categories (X axis) were grouped into three main ontologies: biological process, cellular component, and molecular function. The Y axis indicates the percentage of genes (%).
(TIF)

S3 Fig Histogram representation of the enriched KEGG pathways of unigenes in *A*. *hydrophila*. X axis, KEGG pathway categories; Y axis, statistical significance of enrichment.
(TIF)

## References

[1] Plant K.P., Lapatra S.E. Advances in fish vaccine delivery. Dev. Comp. Immunol. 2011; 35(12): 1256–62. doi: 10.1016/j.dci.2011.03.007 21414351 10.1016/j.dci.2011.03.007

[2] Li H., Qin Y., Yan Q., Lin G., Huang L., Huang B., et al MinD plays an important role in Aeromonas hydrophila adherence to Anguilla japonica mucus. Gene. 2015; 565(2): 275–81. doi: 10.1016/j.gene.2015.04.031 25881868 10.1016/j.gene.2015.04.031

[3] Rodriguez I.I., Novoa B., Figureas A. Immune response of zebrafish (Danio rerio) against a newly isolated bacterial pathogen Aeromonas hydrophila. Fish. Shellfish Immunol. 2008; 25(3): 239–49. doi: 10.1016/j.fsi.2008.05.002 18640853 10.1016/j.fsi.2008.05.002

[4] Mu X.J., Pridgeon J.W., Klesius P.H. Comparative transcriptional analysis reveals distinct expression patterns of channel catfish genes after the first infection and re-infection with Aeromonas hydrophila. Fish. Shellfish Immunol. 2013; 35(5): 1566–76. doi: 10.1016/j.fsi.2013.08.027 24036330 10.1016/j.fsi.2013.08.027 PMC7111657

[5] Kregiel D., Niedzielska K. Effect of plasma processing and organosilane modifications of polyethylene on Aeromonas hydrophila biofilm formation. Biomed Res. Int. 2014: 104. 10.1155/2014/232514 PMC3925535 24605323

[6] Khushiramani R., Girisha S.K., Bhowmick P.P., Karunasagar I., Karunasagar I. Prevalence of different outer membrane proteins in isolates of Aeromonas species. World J. Microbiol. Biotechnol. 2008; 24(10): 2263–8. doi: 10.1007/s11274-008-9740-4

[7] Sahoo P. K., Mahapatra K.D., Saha J. N., Barat A., Sahoo M., et al Family association between immune parameters and resistance to Aeromonas hydrophila infection in the Indian major carp, Labeo rohita. Fish. Shellfish Immunol. 2008; 25(1–2): 163–9. doi: 10.1016/j.fsi.2008.04.003 18486488 10.1016/j.fsi.2008.04.003

[8] Liu L., Gong Y.X., Zhu B., Liu G.L., Wang G.X., Ling F., et al Effect of a new recombinant Aeromonas hydrophila vaccine on the grass carp intestinal microbiota and correlations with immunological responses. Fish. Shellfish Immunol. 2015; 45(1): 175–83. doi: 10.1016/j.fsi.2015.03.043 25862971 10.1016/j.fsi.2015.03.043

[9] Martinez M., Mcdermott P., and Walker R. Pharmacology of the fluoroquinolones: A perspective for the use in domestic animals. Vet J. 2005; 172(1): 10–28. doi: 10.1016/j.tvjl.2005.07.010 16154368 10.1016/j.tvjl.2005.07.010

[10] Del Castillo CS, Hikima J., Jang H.B., Nho S.W., Jung T.S., Wongtavatchai J., et al Comparative sequence analysis of a multidrug-resistant plasmid from Aeromonas hydrophila. 2013; 57(1): 120–9. doi: 10.1128/AAC.01239-12 23070174 10.1128/AAC.01239-12 PMC3535917

[11] Cabello F.C. Heavy use of prophylactic antibiotics in aquaculture: a growing problem for human and animal health and for the environment. Environ. Microbiol. 2006; 8(7): 1137–44. doi: 10.1111/j.1462-2920.2006.01054.x 16817922 10.1111/j.1462-2920.2006.01054.x

[12] Shakir Z., Khan S., Sung K., Khan A., Steele R., Nawaz M. Molecular Characterization of Fluoroquinolone-Resistant Aeromonas spp. Isolated from Imported Shrimp. Applied and Environmental Microbiology. 2012; 78(22): 8137–41. doi: 10.1128/AEM.02081-12 22923408 10.1128/AEM.02081-12 PMC3485934

[13] Li J., Wang T., Shao B., Shen J., Wang S., Wu Y. Plasmid-mediated quinolone resistance genes and antibiotic residues in wastewater and soil adjacent to swine feedlots: potential transfer to agricultural lands. Environ Health Perspect. 2012; 120(8):1144–9. doi: 10.1289/ehp.1104776 22569244 10.1289/ehp.1104776 PMC3440090

[14] Cattoir V., Poirel L., Aubert C., Soussy C.J., Nordmann P. Unexpected Occurrence of Plasmid-Mediated Quinolone Resistance Determinants in Environmental Aeromonas spp. Energing Infectious Diseases. 2008; 14(2): 231–7. doi: 10.3201/eid1402.070677 18258115 10.3201/eid1402.070677 PMC2600179

[15] Wang Z., Gerstein M., Snyder M. RNA-Seq: a revolutionary tool for transcriptomics. Nat Rev Gent. 2009; 10(1): 57–63. doi: 10.1038/nrg2484 19015660 10.1038/nrg2484 PMC2949280

[16] Xiang L.X., He D., Dong W.R., Zhang Y.W., Shao J.Z. Deep sequencing-based transcriptome profiling analysis of bacteria-challenged Lateolabrax japonicas reveals insight into the immune-relevant genes in marine fish. BMC Genomics. 2010; 11(1):472 doi: 10.1186/1471-2164-11-472 20707909 10.1186/1471-2164-11-472 PMC3091668

[17] Czesny S., Epifanio J., Michalak P. Genetic divergence between freshwater and marine morphs of Alewife (*Alosa pseudoharengus*): a ‘next-generation’ sequencing analysis. Plos One. 2012; 7(3): e31803 doi: 10.1371/journal.pone.0031803 22438868 10.1371/journal.pone.0031803 PMC3305293

[18] Cockerill, F.R. Clinical and Laboratory Standards Institute. Performance Standards for Antimicrobial Susceptibility Testing; Twenty-First Informational Supplement Wayne, PA: Clinical and Laboratory Standards Institute, 2011: 165.

[19] Trapnell C., Pachter L., Salzberg S.L. TopHat:discovering splice junctions with RNA-Seq. Bioinformatics. 2009; 25(9): 1105–11. doi: 10.1093/bioinformatics/btp120 19289445 10.1093/bioinformatics/btp120 PMC2672628

[20] Luo R., Liu B., Xie Y., Li Z., Huang W., Yuan J., et al SOAPdenovo2: an empirically improved memory-efficient short-read de novo assembler. Gigascience. 2012; 1(1): 1–6. doi: 10.1186/2047-217X-1-18 23587118 10.1186/2047-217X-1-18 PMC3626529

[21] Pertea G., Huang X., Liang F., Antonescu V., Sultana R., Karamycheva S., et al TIGR Gene Indices clustering tools (TGICL): a software system for fast clustering of large EST datasets. Bioinformatics. 2003; 19(5): 651–2. doi: 10.1093/bioinformatics/btg034 12651724 10.1093/bioinformatics/btg034

[22] Conesa A., Gotz S. Blast2GO: A comprehensive suite for functional analysis in plant genomics. Int J Plant Genomics. 2008; 619832. doi.org/10.1155/2008/619832. 10.1155/2008/619832 PMC2375974 18483572

[23] Li B., Dewey C.N. RSEM: accurate transcript quantification from RNA-Seq data with or without a reference genome. BMC Bioinformatics. 2011; 12(1): 93–99. doi: 10.1186/1471-2105-12-323 21816040 10.1186/1471-2105-12-323 PMC3163565

[24] Anders S., Huber W. Differential expression analysis for sequence count data. Genome Biol. 2010; 11(10): 1–12. doi: 10.1186/gb-2010-11-10-r106 20979621 10.1186/gb-2010-11-10-r106 PMC3218662

[25] Liu B., Jiang G.F., Zhang Y.F., Li J.L., Li X.J., Yue J.S. Analysis of transcriptome differences between resistant and susceptible strains of the citrus red mite Panonychus citri (Acari: Tetranychidae). Plos One. 2011; 6(12): 1159–60. doi: 10.1371/journal.pone.0028516 22162774 10.1371/journal.pone.0028516 PMC3230605

[26] West P.V., Bruijn I.D., Minor K.l., Phillips A.I., Robertson E.J., Wawra S. The putative RxLR effector protein SpHtp1 from the fish pathogenic oomycete Saprolegnia parasitica is translocated into fish cells. FEMS Microbiol. Lett. 2010; 310(2): 127–37. doi: 10.1111/j.1574-6968.2010.02055.x 20659163 10.1111/j.1574-6968.2010.02055.x

[27] Price D.P., Nagarajan V, Churbanov A, Houke LL, Drade P, Milligan B. The fat body transcriptomes of the yellow fever mosquito Aedes aegypti, pre-and post-blood meal. Plos One. 2011; 6(7): e22573 doi: 10.1371/journal.pone.0022573 21818341 10.1371/journal.pone.0022573 PMC3144915

[28] Peng Y., Gao X., Li R., Cao G. Transcriptome sequencing and de novo analysis of Youngia japonica using the Illumina platform. Plos One. 2014; 9(3): e90636 doi: 10.1371/journal.pone.0090636 24595283 10.1371/journal.pone.0090636 PMC3942458

[29] Li C., Weng S., Chen Y., Yu X., Lu L., Zhang H. Analysis of Litopenaeus vannamei transcriptome using the next-generation DNA sequencing technique. Plos One. 2012; 7(10): e47442 doi: 10.1371/journal.pone.0047442 23071809 10.1371/journal.pone.0047442 PMC3469548

[30] Seshadri Rekha, Joseph Sam W., Chopra Ashok K., Sha Jian, Shaw Jonathan, et al Genome Sequence of *Aeromonas hydrophila* ATCC 7966. Jack of All Trades. 2006; 188(23): 8272–82. doi: 10.1128/JB.00621-06 16980456 10.1128/JB.00621-06 PMC1698176

