# Supplementary material for: Transcriptome differences between enrofloxacin-resistant and enrofloxacin-susceptible strains of Aeromonas hydrophila
Source: PLoS One. 2017 Jul 14;12(7):e0179549. doi: 10.1371/journal.pone.0179549 (PMC5510800; doi:10.1371/journal.pone.0179549)
Supplement: S1 Table — (DOC) [file pone.0179549.s001.doc]

***S1 Table. GO analysis for the differential express genes of the Aeromonas hydrophilia.***

| **GO_ID** | **Term** | **Type** | **DEGs_this_term** | **UP** | **Down** | **Expected** | **Pvalue** | **FDR** | **DEGs_list** |
| --- | --- | --- | --- | --- | --- | --- | --- | --- | --- |
| GO:0015942 | Formate metabolic process | biological_process | 5 | 5 | 0 | 0.43 | 2.60E-05 | 0.027404 | AHA_0377,AHA_0378,AHA_0379,AHA_0380,AHA_4145 |
| GO:0019557 | Histidine catabolic process to glutamate and formate | biological_process | 4 | 4 | 0 | 0.29 | 7.10E-05 | 0.033728 | AHA_0377,AHA_0378,AHA_0379,AHA_0380 |
| GO:0009308 | Amine metabolic process | biological_process | 7 | 4 | 3 | 1.2 | 0.00012 | 0.033728 | AHA_0481,AHA_1270,AHA_1834,AHA_2923,AHA_2926,AHA_3000,AHA_3004 |
| GO:0019556 | Histidine catabolic process to glutamate and formamide | biological_process | 4 | 4 | 0 | 0.34 | 0.00016 | 0.033728 | AHA_0377,AHA_0378,AHA_0379,AHA_0380 |
| GO:0043606 | Formamide metabolic process | biological_process | 4 | 4 | 0 | 0.34 | 0.00016 | 0.033728 | AHA_0377,AHA_0378,AHA_0379,AHA_0380 |
| GO:0006548 | Histidine catabolic process | biological_process | 4 | 4 | 0 | 0.39 | 0.00031 | 0.046677143 | AHA_0377,AHA_0378,AHA_0379,AHA_0380 |
| GO:0052805 | Imidazole-containing compound catabolic process | biological_process | 4 | 4 | 0 | 0.39 | 0.00031 | 0.046677143 | AHA_0377,AHA_0378,AHA_0379,AHA_0380 |
| GO:0016706 | Oxidoreductase activity, acting on paired donors, with incorporation or reduction of molecular oxyge... | molecular_function | 3 | 2 | 1 | 0.19 | 0.00041 | 0.0540175 | AHA_1948,AHA_2405,AHA_3601 |
| GO:0006576 | Cellular biogenic amine metabolic process | biological_process | 6 | 4 | 2 | 1.16 | 0.00073 | 0.076942 | AHA_0481,AHA_1270,AHA_2923,AHA_2926,AHA_3000,AHA_3004 |
| GO:0044106 | Cellular amine metabolic process | biological_process | 6 | 4 | 2 | 1.16 | 0.00073 | 0.076942 | AHA_0481,AHA_1270,AHA_2923,AHA_2926,AHA_3000,AHA_3004 |
| GO:0006457 | Protein folding | biological_process | 6 | 6 | 0 | 1.2 | 0.00092 | 0.081685 | AHA_0859,AHA_0860,AHA_2490,AHA_2982,AHA_2983,AHA_2984 |
| GO:0006950 | Response to stress | biological_process | 20 | 16 | 4 | 9.53 | 0.00093 | 0.081685 | AHA_0007,AHA_0008,AHA_0290,AHA_0382,AHA_1110,AHA_1153,AHA_1413,AHA_2405,AHA_2490,AHA_2744,AHA_2982,AHA_2983,AHA_2984,AHA_3004,AHA_3875,AHA_4006,AHA_4008,AHA_4071,AHA_4115,AHA_4145 |
| GO:0048878 | Chemical homeostasis | biological_process | 7 | 5 | 2 | 1.78 | 0.00156 | 0.12648 | AHA_0676,AHA_1110,AHA_1964,AHA_2743,AHA_2744,AHA_3053,AHA_3987 |
| GO:1901605 | Alpha-amino acid metabolic process | biological_process | 18 | 12 | 6 | 8.81 | 0.00233 | 0.16864 | AHA_0377,AHA_0378,AHA_0379,AHA_0380,AHA_0481,AHA_0596,AHA_1015,AHA_1170,AHA_1270,AHA_1956,AHA_2405,AHA_2536,AHA_2807,AHA_2923,AHA_2926,AHA_3949,AHA_4068,AHA_4201 |
| GO:0009376 | HslUV protease complex | cellular_component | 2 | 2 | 0 | 0.1 | 0.0024 | 0.16864 | AHA_4114,AHA_4115 |
| GO:0009445 | Putrescine metabolic process | biological_process | 4 | 2 | 2 | 0.63 | 0.00261 | 0.17193375 | AHA_0481,AHA_1270,AHA_3000,AHA_3004 |
| GO:0006536 | Glutamate metabolic process | biological_process | 4 | 4 | 0 | 0.67 | 0.00351 | 0.20553 | AHA_0377,AHA_0378,AHA_0379,AHA_0380 |
| GO:0006595 | Polyamine metabolic process | biological_process | 4 | 2 | 2 | 0.67 | 0.00351 | 0.20553 | AHA_0481,AHA_1270,AHA_3000,AHA_3004 |
| GO:0055080 | Cation homeostasis | biological_process | 6 | 4 | 2 | 1.68 | 0.00566 | 0.281192143 | AHA_0676,AHA_1110,AHA_1964,AHA_2743,AHA_2744,AHA_3987 |
| GO:0050801 | Ion homeostasis | biological_process | 6 | 4 | 2 | 1.73 | 0.00653 | 0.281192143 | AHA_0676,AHA_1110,AHA_1964,AHA_2743,AHA_2744,AHA_3987 |
| GO:0000502 | proteasome complex | cellular_component | 2 | 2 | 0 | 0.15 | 0.007 | 0.281192143 | AHA_4114,AHA_4115 |
| GO:0009426 | Bacterial-type flagellum basal body, distal rod | cellular_component | 2 | 2 | 0 | 0.15 | 0.007 | 0.281192143 | AHA_2835,AHA_2837 |
| GO:0031597 | Cytosolic proteasome complex | cellular_component | 2 | 2 | 0 | 0.15 | 0.007 | 0.281192143 | AHA_4114,AHA_4115 |
| GO:0009309 | Amine biosynthetic process | biological_process | 3 | 3 | 0 | 0.43 | 0.00739 | 0.281192143 | AHA_1270,AHA_2923,AHA_2926 |
| GO:0009408 | Response to heat | biological_process | 3 | 3 | 0 | 0.43 | 0.00739 | 0.281192143 | AHA_2982,AHA_4071,AHA_4145 |
| GO:0042401 | Cellular biogenic amine biosynthetic process | biological_process | 3 | 3 | 0 | 0.43 | 0.00739 | 0.281192143 | AHA_1270,AHA_2923,AHA_2926 |
| GO:0006547 | Histidine metabolic process | biological_process | 4 | 4 | 0 | 0.82 | 0.00747 | 0.281192143 | AHA_0377,AHA_0378,AHA_0379,AHA_0380 |
| GO:0052803 | Imidazole-containing compound metabolic process | biological_process | 4 | 4 | 0 | 0.82 | 0.00747 | 0.281192143 | AHA_0377,AHA_0378,AHA_0379,AHA_0380 |
| GO:0055072 | Iron ion homeostasis | biological_process | 5 | 4 | 1 | 1.3 | 0.00819 | 0.293231329 | AHA_1110,AHA_1964,AHA_2743,AHA_2744,AHA_3987 |
| GO:0006071 | Glycerol metabolic process | biological_process | 4 | 0 | 4 | 0.87 | 0.00925 | 0.293231329 | AHA_1213,AHA_1652,AHA_4006,AHA_4008 |
| GO:0043225 | Anion transmembrane-transporting ATPase activity | molecular_function | 3 | 2 | 1 | 0.48 | 0.00997 | 0.293231329 | AHA_0608,AHA_1595,AHA_3987 |
| GO:0006598 | Polyamine catabolic process | biological_process | 3 | 1 | 2 | 0.48 | 0.01019 | 0.293231329 | AHA_0481,AHA_3000,AHA_3004 |
| GO:0009310 | Amine catabolic process | biological_process | 3 | 1 | 2 | 0.48 | 0.01019 | 0.293231329 | AHA_0481,AHA_3000,AHA_3004 |
| GO:0009447 | Putrescine catabolic process | biological_process | 3 | 1 | 2 | 0.48 | 0.01019 | 0.293231329 | AHA_0481,AHA_3000,AHA_3004 |
| GO:0042402 | Cellular biogenic amine catabolic process | biological_process | 3 | 1 | 2 | 0.48 | 0.01019 | 0.293231329 | AHA_0481,AHA_3000,AHA_3004 |
| GO:0008784 | Alanine racemase activity | molecular_function | 2 | 1 | 1 | 0.19 | 0.01274 | 0.293231329 | AHA_1015,AHA_2536 |
| GO:0055076 | Transition metal ion homeostasis | biological_process | 5 | 4 | 1 | 1.44 | 0.01289 | 0.293231329 | AHA_1110,AHA_1964,AHA_2743,AHA_2744,AHA_3987 |
| GO:0019520 | Aldonic acid metabolic process | biological_process | 2 | 2 | 0 | 0.19 | 0.01294 | 0.293231329 | AHA_0286,AHA_0290 |
| GO:0019521 | D-Gluconate metabolic process | biological_process | 2 | 2 | 0 | 0.19 | 0.01294 | 0.293231329 | AHA_0286,AHA_0290 |
| GO:0016701 | oxidoreductase activity, acting on single donors with incorporation of molecular oxygen | molecular_function | 3 | 2 | 1 | 0.53 | 0.01324 | 0.293231329 | AHA_1948,AHA_2405,AHA_3601 |
| GO:0016702 | Oxidoreductase activity, acting on single donors with incorporation of molecular oxygen, incorporati... | molecular_function | 3 | 2 | 1 | 0.53 | 0.01324 | 0.293231329 | AHA_1948,AHA_2405,AHA_3601 |
| GO:0016840 | Carbon-nitrogen lyase activity | molecular_function | 3 | 3 | 0 | 0.53 | 0.01324 | 0.293231329 | AHA_0380,AHA_1413,AHA_4201 |
| GO:0030694 | Bacterial-type flagellum basal body, rod | cellular_component | 2 | 2 | 0 | 0.2 | 0.0136 | 0.293231329 | AHA_2835,AHA_2837 |
| GO:0006066 | Alcohol metabolic process | biological_process | 6 | 2 | 4 | 2.02 | 0.0139 | 0.293231329 | AHA_1213,AHA_1248,AHA_1652,AHA_4006,AHA_4008,AHA_4145 |
| GO:0009066 | Aspartate family amino acid metabolic process | biological_process | 6 | 3 | 3 | 2.02 | 0.0139 | 0.293231329 | AHA_1170,AHA_1956,AHA_2807,AHA_3949,AHA_4068,AHA_4201 |
| GO:0019400 | Alditol metabolic process | biological_process | 4 | 0 | 4 | 1.01 | 0.01618 | 0.293231329 | AHA_1213,AHA_1652,AHA_4006,AHA_4008 |
| GO:0042625 | ATPase activity, coupled to transmembrane movement of ions | molecular_function | 5 | 3 | 2 | 1.53 | 0.01638 | 0.293231329 | AHA_0238,AHA_0608,AHA_1595,AHA_3987,AHA_4261 |
| GO:0055065 | Metal ion homeostasis | biological_process | 5 | 4 | 1 | 1.54 | 0.01686 | 0.293231329 | AHA_1110,AHA_1964,AHA_2743,AHA_2744,AHA_3987 |
| GO:0051213 | Dioxygenase activity | molecular_function | 3 | 2 | 1 | 0.57 | 0.01704 | 0.293231329 | AHA_1948,AHA_2405,AHA_3601 |
| GO:1901615 | Organic hydroxy compound metabolic process | biological_process | 7 | 3 | 4 | 2.74 | 0.01797 | 0.293231329 | AHA_0803,AHA_1213,AHA_1248,AHA_1652,AHA_4006,AHA_4008,AHA_4145 |
| GO:0022804 | Active transmembrane transporter activity | molecular_function | 15 | 9 | 6 | 8.5 | 0.02043 | 0.293231329 | AHA_0238,AHA_0286,AHA_0608,AHA_0676,AHA_1419,AHA_1595,AHA_1738,AHA_2806,AHA_2812,AHA_2813,AHA_3004,AHA_3604,AHA_3987,AHA_4006,AHA_4261 |
| GO:0005315 | Inorganic phosphate transmembrane transporter activity | molecular_function | 2 | 2 | 0 | 0.24 | 0.02057 | 0.293231329 | AHA_2812,AHA_2813 |
| GO:0015419 | sulfate transmembrane-transporting ATPase activity | molecular_function | 2 | 1 | 1 | 0.24 | 0.02057 | 0.293231329 | AHA_0608,AHA_3987 |
| GO:0000162 | Tryptophan biosynthetic process | biological_process | 2 | 2 | 0 | 0.24 | 0.02088 | 0.293231329 | AHA_2923,AHA_2926 |
| GO:0006067 | Ethanol metabolic process | biological_process | 2 | 2 | 0 | 0.24 | 0.02088 | 0.293231329 | AHA_1248,AHA_4145 |
| GO:0006523 | Alanine biosynthetic process | biological_process | 2 | 1 | 1 | 0.24 | 0.02088 | 0.293231329 | AHA_1015,AHA_2536 |
| GO:0009079 | Pyruvate family amino acid biosynthetic process | biological_process | 2 | 1 | 1 | 0.24 | 0.02088 | 0.293231329 | AHA_1015,AHA_2536 |
| GO:0030632 | D-alanine biosynthetic process | biological_process | 2 | 1 | 1 | 0.24 | 0.02088 | 0.293231329 | AHA_1015,AHA_2536 |
| GO:0042435 | Indole-containing Compound biosynthetic process | biological_process | 2 | 2 | 0 | 0.24 | 0.02088 | 0.293231329 | AHA_2923,AHA_2926 |
| GO:0046145 | D-Alanine family amino acid biosynthetic process | biological_process | 2 | 1 | 1 | 0.24 | 0.02088 | 0.293231329 | AHA_1015,AHA_2536 |
| GO:0046219 | Indolalkylamine biosynthetic process | biological_process | 2 | 2 | 0 | 0.24 | 0.02088 | 0.293231329 | AHA_2923,AHA_2926 |
| GO:0046437 | D-amino acid biosynthetic process | biological_process | 2 | 1 | 1 | 0.24 | 0.02088 | 0.293231329 | AHA_1015,AHA_2536 |
| GO:0006520 | cellular amino acid metabolic process | biological_process | 19 | 12 | 7 | 11.7 | 0.02101 | 0.293231329 | AHA_0377,AHA_0378,AHA_0379,AHA_0380,AHA_0481,AHA_0596,AHA_1015,AHA_1170,AHA_1270,AHA_1654,AHA_1956,AHA_2405,AHA_2536,AHA_2807,AHA_2923,AHA_2926,AHA_3949,AHA_4068,AHA_4201 |
| GO:0009067 | aspartate family amino acid biosynthetic process | biological_process | 5 | 2 | 3 | 1.64 | 0.02158 | 0.293231329 | AHA_1170,AHA_1956,AHA_2807,AHA_3949,AHA_4068 |
| GO:0046164 | alcohol catabolic process | biological_process | 3 | 1 | 2 | 0.63 | 0.02184 | 0.293231329 | AHA_1248,AHA_4006,AHA_4008 |
| GO:1901616 | organic hydroxy compound catabolic process | biological_process | 3 | 1 | 2 | 0.63 | 0.02184 | 0.293231329 | AHA_1248,AHA_4006,AHA_4008 |
| GO:1901607 | alpha-amino acid biosynthetic process | biological_process | 11 | 7 | 4 | 5.63 | 0.02299 | 0.293231329 | AHA_0596,AHA_1015,AHA_1170,AHA_1956,AHA_2536,AHA_2807,AHA_2923,AHA_2926,AHA_3949,AHA_4068,AHA_4201 |
| GO:0015075 | ion transmembrane transporter activity | molecular_function | 15 | 9 | 6 | 8.74 | 0.02562 | 0.293231329 | AHA_0238,AHA_0286,AHA_0608,AHA_1419,AHA_1595,AHA_2296,AHA_2350,AHA_2743,AHA_2806,AHA_3004,AHA_3053,AHA_3987,AHA_4006,AHA_4261,AHA_4267 |
| GO:0006754 | ATP biosynthetic process | biological_process | 3 | 2 | 1 | 0.67 | 0.02684 | 0.293231329 | AHA_0238,AHA_4261,AHA_4267 |
| GO:0009266 | response to temperature stimulus | biological_process | 3 | 3 | 0 | 0.67 | 0.02684 | 0.293231329 | AHA_2982,AHA_4071,AHA_4145 |
| GO:0022857 | transmembrane transporter activity | molecular_function | 21 | 14 | 7 | 13.7 | 0.02855 | 0.293231329 | AHA_0238,AHA_0286,AHA_0608,AHA_0676,AHA_1419,AHA_1595,AHA_1713,AHA_1738,AHA_2296,AHA_2350,AHA_2743,AHA_2806,AHA_2812,AHA_2813,AHA_3004,AHA_3053,AHA_3604,AHA_3987,AHA_4006,AHA_4261,AHA_4267 |
| GO:0047661 | amino-acid racemase activity | molecular_function | 2 | 1 | 1 | 0.29 | 0.0299 | 0.293231329 | AHA_1015,AHA_2536 |
| GO:0006568 | tryptophan metabolic process | biological_process | 2 | 2 | 0 | 0.29 | 0.03035 | 0.293231329 | AHA_2923,AHA_2926 |
| GO:0006586 | indolalkylamine metabolic process | biological_process | 2 | 2 | 0 | 0.29 | 0.03035 | 0.293231329 | AHA_2923,AHA_2926 |
| GO:0042430 | indole-containing compound metabolic process | biological_process | 2 | 2 | 0 | 0.29 | 0.03035 | 0.293231329 | AHA_2923,AHA_2926 |
| GO:0046144 | D-alanine family amino acid metabolic process | biological_process | 2 | 1 | 1 | 0.29 | 0.03035 | 0.293231329 | AHA_1015,AHA_2536 |
| GO:0046436 | D-alanine metabolic process | biological_process | 2 | 1 | 1 | 0.29 | 0.03035 | 0.293231329 | AHA_1015,AHA_2536 |
| GO:0008652 | cellular amino acid biosynthetic process | biological_process | 12 | 7 | 5 | 6.64 | 0.03099 | 0.293231329 | AHA_0596,AHA_1015,AHA_1170,AHA_1654,AHA_1956,AHA_2536,AHA_2807,AHA_2923,AHA_2926,AHA_3949,AHA_4068,AHA_4201 |
| GO:0009145 | purine nucleoside triphosphate biosynthetic process | biological_process | 3 | 2 | 1 | 0.72 | 0.03239 | 0.293231329 | AHA_0238,AHA_4261,AHA_4267 |
| GO:0009206 | purine ribonucleoside triphosphate biosynthetic process | biological_process | 3 | 2 | 1 | 0.72 | 0.03239 | 0.293231329 | AHA_0238,AHA_4261,AHA_4267 |
| GO:0032787 | monocarboxylic acid metabolic process | biological_process | 10 | 8 | 2 | 5.2 | 0.03254 | 0.293231329 | AHA_0286,AHA_0290,AHA_0377,AHA_0378,AHA_0379,AHA_0380,AHA_1040,AHA_2060,AHA_2405,AHA_4145 |
| GO:0008509 | anion transmembrane transporter activity | molecular_function | 7 | 4 | 3 | 3.1 | 0.03331 | 0.293231329 | AHA_0286,AHA_0608,AHA_1595,AHA_2350,AHA_2806,AHA_3004,AHA_3987 |
| GO:0019751 | polyol metabolic process | biological_process | 4 | 0 | 4 | 1.3 | 0.03801 | 0.293231329 | AHA_1213,AHA_1652,AHA_4006,AHA_4008 |
| GO:0042592 | homeostatic process | biological_process | 7 | 5 | 2 | 3.22 | 0.03979 | 0.293231329 | AHA_0676,AHA_1110,AHA_1964,AHA_2743,AHA_2744,AHA_3053,AHA_3987 |
| GO:0043648 | dicarboxylic acid metabolic process | biological_process | 7 | 6 | 1 | 3.22 | 0.03979 | 0.293231329 | AHA_0377,AHA_0378,AHA_0379,AHA_0380,AHA_1170,AHA_1654,AHA_4145 |
| GO:0008172 | S-methyltransferase activity | molecular_function | 2 | 0 | 2 | 0.33 | 0.04057 | 0.293231329 | AHA_1956,AHA_2807 |
| GO:0015116 | sulfate transmembrane transporter activity | molecular_function | 2 | 1 | 1 | 0.33 | 0.04057 | 0.293231329 | AHA_0608,AHA_3987 |
| GO:0016855 | racemase and epimerase activity, acting on amino acids and derivatives | molecular_function | 2 | 1 | 1 | 0.33 | 0.04057 | 0.293231329 | AHA_1015,AHA_2536 |
| GO:0036361 | racemase activity, acting on amino acids and derivatives | molecular_function | 2 | 1 | 1 | 0.33 | 0.04057 | 0.293231329 | AHA_1015,AHA_2536 |
| GO:1901677 | phosphate transmembrane transporter activity | molecular_function | 2 | 2 | 0 | 0.33 | 0.04057 | 0.293231329 | AHA_2812,AHA_2813 |
| GO:0019752 | carboxylic acid metabolic process | biological_process | 25 | 17 | 8 | 17.71 | 0.04162 | 0.293231329 | AHA_0286,AHA_0290,AHA_0377,AHA_0378,AHA_0379,AHA_0380,AHA_0481,AHA_0596,AHA_1015,AHA_1040,AHA_1170,AHA_1270,AHA_1654,AHA_1956,AHA_2060,AHA_2405,AHA_2536,AHA_2807,AHA_2923,AHA_2926,AHA_3601,AHA_3949,AHA_4068,AHA_4145,AHA_4201 |
| GO:0006974 | cellular response to DNA damage stimulus | biological_process | 9 | 6 | 3 | 4.72 | 0.04354 | 0.293231329 | AHA_0290,AHA_0382,AHA_1153,AHA_1413,AHA_2744,AHA_3004,AHA_3875,AHA_4006,AHA_4008 |
| GO:0043603 | cellular amide metabolic process | biological_process | 5 | 5 | 0 | 1.97 | 0.04453 | 0.293231329 | AHA_0377,AHA_0378,AHA_0379,AHA_0380,AHA_4068 |
| GO:0006555 | methionine metabolic process | biological_process | 3 | 0 | 3 | 0.82 | 0.04515 | 0.293231329 | AHA_1956,AHA_2807,AHA_3949 |
| GO:0009086 | methionine biosynthetic process | biological_process | 3 | 0 | 3 | 0.82 | 0.04515 | 0.293231329 | AHA_1956,AHA_2807,AHA_3949 |
| GO:0015698 | inorganic anion transport | biological_process | 3 | 3 | 0 | 0.82 | 0.04515 | 0.293231329 | AHA_0608,AHA_2812,AHA_2813 |
| GO:0022891 | substrate-specific transmembrane transporter activity | molecular_function | 16 | 10 | 6 | 10.22 | 0.04537 | 0.293231329 | AHA_0238,AHA_0286,AHA_0608,AHA_1419,AHA_1595,AHA_1738,AHA_2296,AHA_2350,AHA_2743,AHA_2806,AHA_3004,AHA_3053,AHA_3987,AHA_4006,AHA_4261,AHA_4267 |
| GO:0000774 | adenyl-nucleotide exchange factor activity | molecular_function | 1 | 1 | 0 | 0.05 | 0.04774 | 0.293231329 | AHA_2984 |
| GO:0003871 | 5-methyltetrahydropteroyltriglutamate-homocysteine S-methyltransferase activity | molecular_function | 1 | 0 | 1 | 0.05 | 0.04774 | 0.293231329 | AHA_1956 |
| GO:0004018 | N6-(1,2-dicarboxyethyl)AMP AMP-lyase (fumarate-forming) activity | molecular_function | 1 | 1 | 0 | 0.05 | 0.04774 | 0.293231329 | AHA_1413 |
| GO:0004298 | threonine-type endopeptidase activity | molecular_function | 1 | 1 | 0 | 0.05 | 0.04774 | 0.293231329 | AHA_4114 |
| GO:0004425 | indole-3-glycerol-phosphate synthase activity | molecular_function | 1 | 1 | 0 | 0.05 | 0.04774 | 0.293231329 | AHA_2926 |
| GO:0004456 | phosphogluconate dehydratase activity | molecular_function | 1 | 1 | 0 | 0.05 | 0.04774 | 0.293231329 | AHA_0288 |
| GO:0004457 | lactate dehydrogenase activity | molecular_function | 1 | 1 | 0 | 0.05 | 0.04774 | 0.293231329 | AHA_4145 |
| GO:0004489 | methylenetetrahydrofolate reductase (NAD(P)H) activity | molecular_function | 1 | 0 | 1 | 0.05 | 0.04774 | 0.293231329 | AHA_3949 |
| GO:0004586 | ornithine decarboxylase activity | molecular_function | 1 | 1 | 0 | 0.05 | 0.04774 | 0.293231329 | AHA_1270 |
| GO:0004640 | phosphoribosylanthranilate isomerase activity | molecular_function | 1 | 1 | 0 | 0.05 | 0.04774 | 0.293231329 | AHA_2926 |
| GO:0004794 | L-threonine ammonia-lyase activity | molecular_function | 1 | 1 | 0 | 0.05 | 0.04774 | 0.293231329 | AHA_4201 |
| GO:0008381 | mechanically-gated ion channel activity | molecular_function | 1 | 1 | 0 | 0.05 | 0.04774 | 0.293231329 | AHA_3053 |
| GO:0008666 | 2,3,4,5-tetrahydropyridine-2,6-dicarboxylate N-succinyltransferase activity | molecular_function | 1 | 1 | 0 | 0.05 | 0.04774 | 0.293231329 | AHA_1170 |
| GO:0008675 | 2-dehydro-3-deoxy-phosphogluconate aldolase activity | molecular_function | 1 | 1 | 0 | 0.05 | 0.04774 | 0.293231329 | AHA_0289 |
| GO:0008700 | 4-hydroxy-2-oxoglutarate aldolase activity | molecular_function | 1 | 1 | 0 | 0.05 | 0.04774 | 0.293231329 | AHA_0289 |
| GO:0008720 | D-lactate dehydrogenase activity | molecular_function | 1 | 1 | 0 | 0.05 | 0.04774 | 0.293231329 | AHA_4145 |
| GO:0008760 | UDP-N-acetylglucosamine 1-carboxyvinyltransferase activity | molecular_function | 1 | 1 | 0 | 0.05 | 0.04774 | 0.293231329 | AHA_3935 |
| GO:0015093 | ferrous iron transmembrane transporter activity | molecular_function | 1 | 1 | 0 | 0.05 | 0.04774 | 0.293231329 | AHA_2743 |
| GO:0015444 | magnesium-importing ATPase activity | molecular_function | 1 | 0 | 1 | 0.05 | 0.04774 | 0.293231329 | AHA_0238 |
| GO:0016153 | urocanate hydratase activity | molecular_function | 1 | 1 | 0 | 0.05 | 0.04774 | 0.293231329 | AHA_0379 |
| GO:0016992 | lipoate synthase activity | molecular_function | 1 | 1 | 0 | 0.05 | 0.04774 | 0.293231329 | AHA_3263 |
| GO:0022833 | mechanically gated channel activity | molecular_function | 1 | 1 | 0 | 0.05 | 0.04774 | 0.293231329 | AHA_3053 |
| GO:0031543 | peptidyl-proline dioxygenase activity | molecular_function | 1 | 0 | 1 | 0.05 | 0.04774 | 0.293231329 | AHA_2405 |
| GO:0031545 | peptidyl-proline 4-dioxygenase activity | molecular_function | 1 | 0 | 1 | 0.05 | 0.04774 | 0.293231329 | AHA_2405 |
| GO:0033856 | pyridoxine 5'-phosphate synthase activity | molecular_function | 1 | 1 | 0 | 0.05 | 0.04774 | 0.293231329 | AHA_0803 |
| GO:0033969 | gamma-glutamyl-gamma-aminobutyrate hydrolase activity | molecular_function | 1 | 0 | 1 | 0.05 | 0.04774 | 0.293231329 | AHA_0481 |
| GO:0042085 | 5-methyltetrahydropteroyltri-L-glutamate-dependent methyltransferase activity | molecular_function | 1 | 0 | 1 | 0.05 | 0.04774 | 0.293231329 | AHA_1956 |
| GO:0043956 | 3-hydroxypropionyl-CoA dehydratase activity | molecular_function | 1 | 0 | 1 | 0.05 | 0.04774 | 0.293231329 | AHA_2060 |
| GO:0045543 | gibberellin 2-beta-dioxygenase activity | molecular_function | 1 | 1 | 0 | 0.05 | 0.04774 | 0.293231329 | AHA_3601 |
| GO:0047324 | phosphoenolpyruvate-glycerone phosphotransferase activity | molecular_function | 1 | 0 | 1 | 0.05 | 0.04774 | 0.293231329 | AHA_4008 |
| GO:0050415 | formimidoylglutamase activity | molecular_function | 1 | 1 | 0 | 0.05 | 0.04774 | 0.293231329 | AHA_0378 |
| GO:0050480 | imidazolonepropionase activity | molecular_function | 1 | 1 | 0 | 0.05 | 0.04774 | 0.293231329 | AHA_0377 |
| GO:0070003 | threonine-type peptidase activity | molecular_function | 1 | 1 | 0 | 0.05 | 0.04774 | 0.293231329 | AHA_4114 |
| GO:0070626 | (S)-2-(5-amino-1-(5-phospho-D-ribosyl)imidazole-4-carboxamido)succinate AMP-lyase (fumarate-forming)... | molecular_function | 1 | 1 | 0 | 0.05 | 0.04774 | 0.293231329 | AHA_1413 |
| GO:0034220 | ion transmembrane transport | biological_process | 4 | 4 | 0 | 1.4 | 0.0478 | 0.293231329 | AHA_2744,AHA_2812,AHA_4261,AHA_4267 |
| GO:0000920 | cytokinetic cell separation | biological_process | 1 | 1 | 0 | 0.05 | 0.04813 | 0.293231329 | AHA_0292 |
| GO:0001702 | gastrulation with mouth forming second | biological_process | 1 | 0 | 1 | 0.05 | 0.04813 | 0.293231329 | AHA_1921 |
| GO:0001896 | autolysis | biological_process | 1 | 1 | 0 | 0.05 | 0.04813 | 0.293231329 | AHA_0292 |
| GO:0006013 | mannose metabolic process | biological_process | 1 | 0 | 1 | 0.05 | 0.04813 | 0.293231329 | AHA_2338 |
| GO:0006068 | ethanol catabolic process | biological_process | 1 | 1 | 0 | 0.05 | 0.04813 | 0.293231329 | AHA_1248 |
| GO:0006103 | 2-oxoglutarate metabolic process | biological_process | 1 | 1 | 0 | 0.05 | 0.04813 | 0.293231329 | AHA_4145 |
| GO:0006884 | cell volume homeostasis | biological_process | 1 | 1 | 0 | 0.05 | 0.04813 | 0.293231329 | AHA_3053 |
| GO:0007162 | negative regulation of cell adhesion | biological_process | 1 | 0 | 1 | 0.05 | 0.04813 | 0.293231329 | AHA_1921 |
| GO:0007369 | gastrulation | biological_process | 1 | 0 | 1 | 0.05 | 0.04813 | 0.293231329 | AHA_1921 |
| GO:0009255 | Entner-Doudoroff pathway | biological_process | 1 | 1 | 0 | 0.05 | 0.04813 | 0.293231329 | AHA_0288 |
| GO:0009685 | gibberellin metabolic process | biological_process | 1 | 1 | 0 | 0.05 | 0.04813 | 0.293231329 | AHA_3601 |
| GO:0009686 | gibberellin biosynthetic process | biological_process | 1 | 1 | 0 | 0.05 | 0.04813 | 0.293231329 | AHA_3601 |
| GO:0009992 | cellular water homeostasis | biological_process | 1 | 1 | 0 | 0.05 | 0.04813 | 0.293231329 | AHA_3053 |
| GO:0016101 | diterpenoid metabolic process | biological_process | 1 | 1 | 0 | 0.05 | 0.04813 | 0.293231329 | AHA_3601 |
| GO:0016102 | diterpenoid biosynthetic process | biological_process | 1 | 1 | 0 | 0.05 | 0.04813 | 0.293231329 | AHA_3601 |
| GO:0018401 | peptidyl-proline hydroxylation to 4-hydroxy-L-proline | biological_process | 1 | 0 | 1 | 0.05 | 0.04813 | 0.293231329 | AHA_2405 |
| GO:0019471 | 4-hydroxyproline metabolic process | biological_process | 1 | 0 | 1 | 0.05 | 0.04813 | 0.293231329 | AHA_2405 |
| GO:0019511 | peptidyl-proline hydroxylation | biological_process | 1 | 0 | 1 | 0.05 | 0.04813 | 0.293231329 | AHA_2405 |
| GO:0019654 | acetate fermentation | biological_process | 1 | 1 | 0 | 0.05 | 0.04813 | 0.293231329 | AHA_4145 |
| GO:0019655 | glucose catabolic process to ethanol | biological_process | 1 | 1 | 0 | 0.05 | 0.04813 | 0.293231329 | AHA_4145 |
| GO:0019658 | glucose catabolic process to lactate and acetate | biological_process | 1 | 1 | 0 | 0.05 | 0.04813 | 0.293231329 | AHA_4145 |
| GO:0019659 | glucose catabolic process to lactate | biological_process | 1 | 1 | 0 | 0.05 | 0.04813 | 0.293231329 | AHA_4145 |
| GO:0019660 | glycolytic fermentation | biological_process | 1 | 1 | 0 | 0.05 | 0.04813 | 0.293231329 | AHA_4145 |
| GO:0019664 | glucose catabolic process to mixed acids | biological_process | 1 | 1 | 0 | 0.05 | 0.04813 | 0.293231329 | AHA_4145 |
| GO:0030104 | water homeostasis | biological_process | 1 | 1 | 0 | 0.05 | 0.04813 | 0.293231329 | AHA_3053 |
| GO:0030155 | regulation of cell adhesion | biological_process | 1 | 0 | 1 | 0.05 | 0.04813 | 0.293231329 | AHA_1921 |
| GO:0030582 | fruiting body development | biological_process | 1 | 0 | 1 | 0.05 | 0.04813 | 0.293231329 | AHA_2405 |
| GO:0030587 | sorocarp development | biological_process | 1 | 0 | 1 | 0.05 | 0.04813 | 0.293231329 | AHA_2405 |
| GO:0031154 | culmination involved in sorocarp development | biological_process | 1 | 0 | 1 | 0.05 | 0.04813 | 0.293231329 | AHA_2405 |
| GO:0033387 | putrescine biosynthetic process from ornithine | biological_process | 1 | 1 | 0 | 0.05 | 0.04813 | 0.293231329 | AHA_1270 |
| GO:0034310 | primary alcohol catabolic process | biological_process | 1 | 1 | 0 | 0.05 | 0.04813 | 0.293231329 | AHA_1248 |
| GO:0034755 | iron ion transmembrane transport | biological_process | 1 | 1 | 0 | 0.05 | 0.04813 | 0.293231329 | AHA_2744 |
| GO:0048608 | reproductive structure development | biological_process | 1 | 0 | 1 | 0.05 | 0.04813 | 0.293231329 | AHA_2405 |
| GO:0055084 | fruiting body development in response to starvation | biological_process | 1 | 0 | 1 | 0.05 | 0.04813 | 0.293231329 | AHA_2405 |
| GO:0061458 | reproductive system development | biological_process | 1 | 0 | 1 | 0.05 | 0.04813 | 0.293231329 | AHA_2405 |
| GO:0070627 | ferrous iron import | biological_process | 1 | 1 | 0 | 0.05 | 0.04813 | 0.293231329 | AHA_2744 |
| GO:0072592 | oxygen metabolic process | biological_process | 1 | 0 | 1 | 0.05 | 0.04813 | 0.293231329 | AHA_2405 |
| GO:0075259 | spore-bearing organ development | biological_process | 1 | 0 | 1 | 0.05 | 0.04813 | 0.293231329 | AHA_2405 |
| GO:0097286 | iron ion import | biological_process | 1 | 1 | 0 | 0.05 | 0.04813 | 0.293231329 | AHA_2744 |
| GO:1902706 | hexose catabolic process to acetate | biological_process | 1 | 1 | 0 | 0.05 | 0.04813 | 0.293231329 | AHA_4145 |
| GO:1902707 | hexose catabolic process to ethanol | biological_process | 1 | 1 | 0 | 0.05 | 0.04813 | 0.293231329 | AHA_4145 |
| GO:0009273 | peptidoglycan-based cell wall biogenesis | biological_process | 5 | 3 | 2 | 2.02 | 0.04867 | 0.294817126 | AHA_1015,AHA_2130,AHA_2536,AHA_3935,AHA_4258 |
| GO:0005839 | proteasome core complex | cellular_component | 1 | 1 | 0 | 0.05 | 0.0494 | 0.295838636 | AHA_4114 |
| GO:0009428 | bacterial-type flagellum basal body, distal rod, P ring | cellular_component | 1 | 1 | 0 | 0.05 | 0.0494 | 0.295838636 | AHA_2835 |
| GO:0022892 | substrate-specific transporter activity | molecular_function | 19 | 12 | 7 | 12.89 | 0.05162 | 0.305252376 | AHA_0238,AHA_0286,AHA_0608,AHA_1419,AHA_1595,AHA_1738,AHA_1866,AHA_2296,AHA_2350,AHA_2743,AHA_2806,AHA_3004,AHA_3053,AHA_3324,AHA_3769,AHA_3987,AHA_4006,AHA_4261,AHA_4267 |
| GO:0015291 | secondary active transmembrane transporter activity | molecular_function | 9 | 5 | 4 | 4.87 | 0.05198 | 0.305252376 | AHA_0286,AHA_0676,AHA_1419,AHA_2806,AHA_2812,AHA_2813,AHA_3004,AHA_3604,AHA_4006 |
| GO:0009073 | aromatic amino acid family biosynthetic process | biological_process | 3 | 2 | 1 | 0.87 | 0.05233 | 0.305252376 | AHA_1654,AHA_2923,AHA_2926 |
| GO:0009201 | ribonucleoside triphosphate biosynthetic process | biological_process | 3 | 2 | 1 | 0.87 | 0.05233 | 0.305252376 | AHA_0238,AHA_4261,AHA_4267 |
| GO:0016841 | ammonia-lyase activity | molecular_function | 2 | 2 | 0 | 0.38 | 0.05242 | 0.305252376 | AHA_0380,AHA_4201 |
| GO:0034308 | primary alcohol metabolic process | biological_process | 2 | 2 | 0 | 0.39 | 0.05319 | 0.306351148 | AHA_1248,AHA_4145 |
| GO:0046416 | D-amino acid metabolic process | biological_process | 2 | 1 | 1 | 0.39 | 0.05319 | 0.306351148 | AHA_1015,AHA_2536 |
| GO:0005783 | endoplasmic reticulum | cellular_component | 2 | 0 | 2 | 0.4 | 0.0557 | 0.318251027 | AHA_1921,AHA_2046 |
| GO:0043565 | sequence-specific DNA binding | molecular_function | 6 | 2 | 4 | 2.77 | 0.05586 | 0.318251027 | AHA_1213,AHA_2538,AHA_3000,AHA_3606,AHA_3821,AHA_4067 |
| GO:0042546 | cell wall biogenesis | biological_process | 5 | 3 | 2 | 2.12 | 0.05761 | 0.326456667 | AHA_1015,AHA_2130,AHA_2536,AHA_3935,AHA_4258 |
| GO:0009064 | glutamine family amino acid metabolic process | biological_process | 6 | 5 | 1 | 2.84 | 0.06164 | 0.347425455 | AHA_0377,AHA_0378,AHA_0379,AHA_0380,AHA_0481,AHA_0596 |
| GO:0043436 | oxoacid metabolic process | biological_process | 25 | 17 | 8 | 18.43 | 0.06251 | 0.350455 | AHA_0286,AHA_0290,AHA_0377,AHA_0378,AHA_0379,AHA_0380,AHA_0481,AHA_0596,AHA_1015,AHA_1040,AHA_1170,AHA_1270,AHA_1654,AHA_1956,AHA_2060,AHA_2405,AHA_2536,AHA_2807,AHA_2923,AHA_2926,AHA_3601,AHA_3949,AHA_4068,AHA_4145,AHA_4201 |
| GO:0016812 | hydrolase activity, acting on carbon-nitrogen (but not peptide) bonds, in cyclic amides | molecular_function | 2 | 2 | 0 | 0.43 | 0.06534 | 0.356370306 | AHA_0377,AHA_4258 |
| GO:0072509 | divalent inorganic cation transmembrane transporter activity | molecular_function | 2 | 1 | 1 | 0.43 | 0.06534 | 0.356370306 | AHA_0238,AHA_2743 |
| GO:1901682 | sulfur compound transmembrane transporter activity | molecular_function | 2 | 1 | 1 | 0.43 | 0.06534 | 0.356370306 | AHA_0608,AHA_3987 |
| GO:0006522 | alanine metabolic process | biological_process | 2 | 1 | 1 | 0.43 | 0.06627 | 0.356370306 | AHA_1015,AHA_2536 |
| GO:0009078 | pyruvate family amino acid metabolic process | biological_process | 2 | 1 | 1 | 0.43 | 0.06627 | 0.356370306 | AHA_1015,AHA_2536 |
| GO:0015985 | energy coupled proton transport, down electrochemical gradient | biological_process | 2 | 2 | 0 | 0.43 | 0.06627 | 0.356370306 | AHA_4261,AHA_4267 |
| GO:0015986 | ATP synthesis coupled proton transport | biological_process | 2 | 2 | 0 | 0.43 | 0.06627 | 0.356370306 | AHA_4261,AHA_4267 |
| GO:0019563 | glycerol catabolic process | biological_process | 2 | 0 | 2 | 0.43 | 0.06627 | 0.356370306 | AHA_4006,AHA_4008 |
| GO:0015103 | inorganic anion transmembrane transporter activity | molecular_function | 3 | 2 | 1 | 0.95 | 0.06694 | 0.35814599 | AHA_0608,AHA_1595,AHA_3987 |
| GO:0009142 | nucleoside triphosphate biosynthetic process | biological_process | 3 | 2 | 1 | 0.96 | 0.06822 | 0.363150909 | AHA_0238,AHA_4261,AHA_4267 |
| GO:0044445 | cytosolic part | cellular_component | 2 | 2 | 0 | 0.44 | 0.0693 | 0.365211 | AHA_4114,AHA_4115 |
| GO:0045259 | proton-transporting ATP synthase complex | cellular_component | 2 | 2 | 0 | 0.44 | 0.0693 | 0.365211 | AHA_4261,AHA_4267 |
| GO:0006082 | organic acid metabolic process | biological_process | 25 | 17 | 8 | 18.72 | 0.07268 | 0.375128182 | AHA_0286,AHA_0290,AHA_0377,AHA_0378,AHA_0379,AHA_0380,AHA_0481,AHA_0596,AHA_1015,AHA_1040,AHA_1170,AHA_1270,AHA_1654,AHA_1956,AHA_2060,AHA_2405,AHA_2536,AHA_2807,AHA_2923,AHA_2926,AHA_3601,AHA_3949,AHA_4068,AHA_4145,AHA_4201 |
| GO:0009628 | response to abiotic stimulus | biological_process | 3 | 3 | 0 | 1.01 | 0.07689 | 0.375128182 | AHA_2982,AHA_4071,AHA_4145 |
| GO:0044275 | cellular carbohydrate catabolic process | biological_process | 3 | 1 | 2 | 1.01 | 0.07689 | 0.375128182 | AHA_0290,AHA_4006,AHA_4008 |
| GO:0005381 | iron ion transmembrane transporter activity | molecular_function | 2 | 1 | 1 | 0.48 | 0.07918 | 0.375128182 | AHA_2743,AHA_3987 |
| GO:0016833 | oxo-acid-lyase activity | molecular_function | 2 | 2 | 0 | 0.48 | 0.07918 | 0.375128182 | AHA_0289,AHA_2923 |
| GO:0044282 | small molecule catabolic process | biological_process | 8 | 6 | 2 | 4.52 | 0.07971 | 0.375128182 | AHA_0290,AHA_0377,AHA_0378,AHA_0379,AHA_0380,AHA_1248,AHA_4006,AHA_4008 |
| GO:0006817 | phosphate ion transport | biological_process | 2 | 2 | 0 | 0.48 | 0.08029 | 0.375128182 | AHA_2812,AHA_2813 |
| GO:0007275 | multicellular organismal development | biological_process | 2 | 0 | 2 | 0.48 | 0.08029 | 0.375128182 | AHA_1921,AHA_2405 |
| GO:0019405 | alditol catabolic process | biological_process | 2 | 0 | 2 | 0.48 | 0.08029 | 0.375128182 | AHA_4006,AHA_4008 |
| GO:0046700 | heterocycle catabolic process | biological_process | 11 | 10 | 1 | 6.93 | 0.08251 | 0.375128182 | AHA_0278,AHA_0377,AHA_0378,AHA_0379,AHA_0380,AHA_0608,AHA_1008,AHA_3545,AHA_3987,AHA_4115,AHA_4285 |
| GO:0065008 | regulation of biological quality | biological_process | 11 | 7 | 4 | 6.93 | 0.08251 | 0.375128182 | AHA_0676,AHA_1015,AHA_1110,AHA_1964,AHA_2130,AHA_2536,AHA_2743,AHA_2744,AHA_3053,AHA_3935,AHA_3987 |
| GO:0016469 | proton-transporting two-sector ATPase complex | cellular_component | 2 | 2 | 0 | 0.49 | 0.084 | 0.375128182 | AHA_4261,AHA_4267 |
| GO:0046394 | carboxylic acid biosynthetic process | biological_process | 14 | 9 | 5 | 9.43 | 0.08422 | 0.375128182 | AHA_0596,AHA_1015,AHA_1040,AHA_1170,AHA_1654,AHA_1956,AHA_2536,AHA_2807,AHA_2923,AHA_2926,AHA_3601,AHA_3949,AHA_4068,AHA_4201 |
| GO:0016705 | oxidoreductase activity, acting on paired donors, with incorporation or reduction of molecular oxyge... | molecular_function | 3 | 2 | 1 | 1.05 | 0.08445 | 0.375128182 | AHA_1948,AHA_2405,AHA_3601 |
| GO:0055085 | transmembrane transport | biological_process | 10 | 10 | 0 | 6.16 | 0.08492 | 0.375128182 | AHA_0022,AHA_0847,AHA_1713,AHA_1738,AHA_2744,AHA_2812,AHA_3053,AHA_3604,AHA_4261,AHA_4267 |
| GO:0044270 | cellular nitrogen compound catabolic process | biological_process | 11 | 10 | 1 | 6.98 | 0.08573 | 0.375128182 | AHA_0278,AHA_0377,AHA_0378,AHA_0379,AHA_0380,AHA_0608,AHA_1008,AHA_3545,AHA_3987,AHA_4115,AHA_4285 |
| GO:0048856 | anatomical structure development | biological_process | 6 | 2 | 4 | 3.13 | 0.08973 | 0.375128182 | AHA_1015,AHA_1921,AHA_2130,AHA_2405,AHA_2536,AHA_3935 |
| GO:0016053 | organic acid biosynthetic process | biological_process | 14 | 9 | 5 | 9.53 | 0.08987 | 0.375128182 | AHA_0596,AHA_1015,AHA_1040,AHA_1170,AHA_1654,AHA_1956,AHA_2536,AHA_2807,AHA_2923,AHA_2926,AHA_3601,AHA_3949,AHA_4068,AHA_4201 |
| GO:0044424 | intracellular part | cellular_component | 50 | 35 | 15 | 42.87 | 0.0899 | 0.375128182 | AHA_0007,AHA_0008,AHA_0262,AHA_0289,AHA_0295,AHA_0315,AHA_0377,AHA_0379,AHA_0380,AHA_0596,AHA_0617,AHA_0625,AHA_0677,AHA_0724,AHA_0803,AHA_0859,AHA_0860,AHA_1040,AHA_1170,AHA_1391,AHA_1419,AHA_1522,AHA_1652,AHA_1805,AHA_1921,AHA_1964,AHA_2046,AHA_2305,AHA_2333,AHA_2405,AHA_2490,AHA_2715,AHA_2835,AHA_2837,AHA_2982,AHA_2984,AHA_3125,AHA_3263,AHA_3877,AHA_3935,AHA_3949,AHA_4006,AHA_4008,AHA_4031,AHA_4068,AHA_4071,AHA_4114,AHA_4115,AHA_4261,AHA_4267 |
| GO:0000030 | mannosyltransferase activity | molecular_function | 1 | 1 | 0 | 0.1 | 0.09321 | 0.375128182 | AHA_0988 |
| GO:0003841 | 1-acylglycerol-3-phosphate O-acyltransferase activity | molecular_function | 1 | 1 | 0 | 0.1 | 0.09321 | 0.375128182 | AHA_3740 |
| GO:0004049 | anthranilate synthase activity | molecular_function | 1 | 1 | 0 | 0.1 | 0.09321 | 0.375128182 | AHA_2923 |
| GO:0004055 | argininosuccinate synthase activity | molecular_function | 1 | 1 | 0 | 0.1 | 0.09321 | 0.375128182 | AHA_0596 |
| GO:0004071 | aspartate-ammonia ligase activity | molecular_function | 1 | 1 | 0 | 0.1 | 0.09321 | 0.375128182 | AHA_4068 |
| GO:0004371 | glycerone kinase activity | molecular_function | 1 | 0 | 1 | 0.1 | 0.09321 | 0.375128182 | AHA_4008 |
| GO:0004397 | histidine ammonia-lyase activity | molecular_function | 1 | 1 | 0 | 0.1 | 0.09321 | 0.375128182 | AHA_0380 |
| GO:0004596 | peptide alpha-N-acetyltransferase activity | molecular_function | 1 | 0 | 1 | 0.1 | 0.09321 | 0.375128182 | AHA_0724 |
| GO:0004834 | tryptophan synthase activity | molecular_function | 1 | 1 | 0 | 0.1 | 0.09321 | 0.375128182 | AHA_2928 |
| GO:0008413 | 8-oxo-7,8-dihydroguanosine triphosphate pyrophosphatase activity | molecular_function | 1 | 1 | 0 | 0.1 | 0.09321 | 0.375128182 | AHA_3875 |
| GO:0008748 | N-ethylmaleimide reductase activity | molecular_function | 1 | 1 | 0 | 0.1 | 0.09321 | 0.375128182 | AHA_0735 |
| GO:0008898 | homocysteine S-methyltransferase activity | molecular_function | 1 | 0 | 1 | 0.1 | 0.09321 | 0.375128182 | AHA_2807 |
| GO:0008923 | lysine decarboxylase activity | molecular_function | 1 | 1 | 0 | 0.1 | 0.09321 | 0.375128182 | AHA_1270 |
| GO:0008999 | ribosomal-protein-alanine N-acetyltransferase activity | molecular_function | 1 | 0 | 1 | 0.1 | 0.09321 | 0.375128182 | AHA_0724 |
| GO:0015091 | ferric iron transmembrane transporter activity | molecular_function | 1 | 0 | 1 | 0.1 | 0.09321 | 0.375128182 | AHA_3987 |
| GO:0015173 | aromatic amino acid transmembrane transporter activity | molecular_function | 1 | 0 | 1 | 0.1 | 0.09321 | 0.375128182 | AHA_2350 |
| GO:0015198 | oligopeptide transporter activity | molecular_function | 1 | 1 | 0 | 0.1 | 0.09321 | 0.375128182 | AHA_3324 |
| GO:0015408 | ferric-transporting ATPase activity | molecular_function | 1 | 0 | 1 | 0.1 | 0.09321 | 0.375128182 | AHA_3987 |
| GO:0015412 | molybdate transmembrane-transporting ATPase activity | molecular_function | 1 | 1 | 0 | 0.1 | 0.09321 | 0.375128182 | AHA_1595 |
| GO:0016749 | N-succinyltransferase activity | molecular_function | 1 | 1 | 0 | 0.1 | 0.09321 | 0.375128182 | AHA_1170 |
| GO:0016842 | amidine-lyase activity | molecular_function | 1 | 1 | 0 | 0.1 | 0.09321 | 0.375128182 | AHA_1413 |
| GO:0016979 | lipoate-protein ligase activity | molecular_function | 1 | 1 | 0 | 0.1 | 0.09321 | 0.375128182 | AHA_3263 |
| GO:0031418 | L-ascorbic acid binding | molecular_function | 1 | 0 | 1 | 0.1 | 0.09321 | 0.375128182 | AHA_2405 |
| GO:0033883 | pyridoxal phosphatase activity | molecular_function | 1 | 1 | 0 | 0.1 | 0.09321 | 0.375128182 | AHA_3603 |
| GO:0034212 | peptide N-acetyltransferase activity | molecular_function | 1 | 0 | 1 | 0.1 | 0.09321 | 0.375128182 | AHA_0724 |
| GO:0042171 | lysophosphatidic acid acyltransferase activity | molecular_function | 1 | 1 | 0 | 0.1 | 0.09321 | 0.375128182 | AHA_3740 |
| GO:0042936 | dipeptide transporter activity | molecular_function | 1 | 1 | 0 | 0.1 | 0.09321 | 0.375128182 | AHA_3324 |
| GO:0060590 | ATPase regulator activity | molecular_function | 1 | 1 | 0 | 0.1 | 0.09321 | 0.375128182 | AHA_2984 |
| GO:0070404 | NADH binding | molecular_function | 1 | 1 | 0 | 0.1 | 0.09321 | 0.375128182 | AHA_4145 |
| GO:0071617 | lysophospholipid acyltransferase activity | molecular_function | 1 | 1 | 0 | 0.1 | 0.09321 | 0.375128182 | AHA_3740 |
| GO:0072510 | trivalent inorganic cation transmembrane transporter activity | molecular_function | 1 | 0 | 1 | 0.1 | 0.09321 | 0.375128182 | AHA_3987 |
| GO:0006089 | lactate metabolic process | biological_process | 1 | 1 | 0 | 0.1 | 0.09396 | 0.375128182 | AHA_4145 |
| GO:0006493 | protein O-linked glycosylation | biological_process | 1 | 1 | 0 | 0.1 | 0.09396 | 0.375128182 | AHA_0988 |
| GO:0006529 | asparagine biosynthetic process | biological_process | 1 | 1 | 0 | 0.1 | 0.09396 | 0.375128182 | AHA_4068 |
| GO:0007219 | Notch signaling pathway | biological_process | 1 | 0 | 1 | 0.1 | 0.09396 | 0.375128182 | AHA_2046 |
| GO:0008216 | spermidine metabolic process | biological_process | 1 | 1 | 0 | 0.1 | 0.09396 | 0.375128182 | AHA_1270 |
| GO:0008295 | spermidine biosynthetic process | biological_process | 1 | 1 | 0 | 0.1 | 0.09396 | 0.375128182 | AHA_1270 |
| GO:0017038 | protein import | biological_process | 1 | 1 | 0 | 0.1 | 0.09396 | 0.375128182 | AHA_3877 |
| GO:0019276 | UDP-N-acetylgalactosamine metabolic process | biological_process | 1 | 1 | 0 | 0.1 | 0.09396 | 0.375128182 | AHA_3935 |
| GO:0019277 | UDP-N-acetylgalactosamine biosynthetic process | biological_process | 1 | 1 | 0 | 0.1 | 0.09396 | 0.375128182 | AHA_3935 |
| GO:0035435 | phosphate ion transmembrane transport | biological_process | 1 | 1 | 0 | 0.1 | 0.09396 | 0.375128182 | AHA_2812 |
| GO:0042026 | protein refolding | biological_process | 1 | 1 | 0 | 0.1 | 0.09396 | 0.375128182 | AHA_0860 |
| GO:0044208 | 'de novo' AMP biosynthetic process | biological_process | 1 | 1 | 0 | 0.1 | 0.09396 | 0.375128182 | AHA_1413 |
| GO:0070981 | L-asparagine biosynthetic process | biological_process | 1 | 1 | 0 | 0.1 | 0.09396 | 0.375128182 | AHA_4068 |
| GO:0070982 | L-asparagine metabolic process | biological_process | 1 | 1 | 0 | 0.1 | 0.09396 | 0.375128182 | AHA_4068 |
| GO:0006605 | protein targeting | biological_process | 2 | 2 | 0 | 0.53 | 0.09513 | 0.378366113 | AHA_1738,AHA_3877 |
| GO:0050896 | response to stimulus | biological_process | 32 | 25 | 7 | 25.75 | 0.09844 | 0.388145259 | AHA_0007,AHA_0008,AHA_0290,AHA_0292,AHA_0382,AHA_0735,AHA_0847,AHA_1110,AHA_1153,AHA_1213,AHA_1338,AHA_1391,AHA_1413,AHA_1713,AHA_2046,AHA_2405,AHA_2490,AHA_2744,AHA_2982,AHA_2983,AHA_2984,AHA_3004,AHA_3324,AHA_3561,AHA_3604,AHA_3875,AHA_4006,AHA_4008,AHA_4071,AHA_4115,AHA_4145,AHA_4258 |
| GO:0009127 | purine nucleoside monophosphate biosynthetic process | biological_process | 4 | 3 | 1 | 1.78 | 0.09937 | 0.388145259 | AHA_0238,AHA_1413,AHA_4261,AHA_4267 |
| GO:0009168 | purine ribonucleoside monophosphate biosynthetic process | biological_process | 4 | 3 | 1 | 1.78 | 0.09937 | 0.388145259 | AHA_0238,AHA_1413,AHA_4261,AHA_4267 |
| GO:0012505 | endomembrane system | cellular_component | 2 | 0 | 2 | 0.54 | 0.0994 | 0.388145259 | AHA_1921,AHA_2046 |
| GO:1901361 | organic cyclic compound catabolic process | biological_process | 11 | 10 | 1 | 7.17 | 0.09943 | 0.388145259 | AHA_0278,AHA_0377,AHA_0378,AHA_0379,AHA_0380,AHA_0608,AHA_1008,AHA_3545,AHA_3987,AHA_4115,AHA_4285 |
| GO:0046034 | ATP metabolic process | biological_process | 8 | 6 | 2 | 4.76 | 0.10064 | 0.391419041 | AHA_0238,AHA_0608,AHA_3545,AHA_3987,AHA_4115,AHA_4261,AHA_4267,AHA_4285 |
| GO:0009205 | purine ribonucleoside triphosphate metabolic process | biological_process | 9 | 7 | 2 | 5.58 | 0.10266 | 0.3978075 | AHA_0238,AHA_0278,AHA_0608,AHA_3545,AHA_3987,AHA_4115,AHA_4261,AHA_4267,AHA_4285 |
| GO:0005622 | intracellular | cellular_component | 58 | 40 | 18 | 51.17 | 0.1036 | 0.398865912 | AHA_0007,AHA_0008,AHA_0262,AHA_0278,AHA_0289,AHA_0290,AHA_0295,AHA_0315,AHA_0377,AHA_0379,AHA_0380,AHA_0596,AHA_0617,AHA_0625,AHA_0677,AHA_0724,AHA_0803,AHA_0859,AHA_0860,AHA_1040,AHA_1170,AHA_1213,AHA_1338,AHA_1391,AHA_1419,AHA_1522,AHA_1652,AHA_1738,AHA_1805,AHA_1921,AHA_1964,AHA_2046,AHA_2305,AHA_2333,AHA_2405,AHA_2490,AHA_2715,AHA_2835,AHA_2837,AHA_2982,AHA_2984,AHA_3125,AHA_3263,AHA_3606,AHA_3821,AHA_3877,AHA_3935,AHA_3949,AHA_4006,AHA_4008,AHA_4031,AHA_4067,AHA_4068,AHA_4071,AHA_4114,AHA_4115,AHA_4261,AHA_4267 |
| GO:0019829 | cation-transporting ATPase activity | molecular_function | 3 | 1 | 2 | 1.15 | 0.10369 | 0.398865912 | AHA_0238,AHA_3987,AHA_4261 |
| GO:0016836 | hydro-lyase activity | molecular_function | 4 | 3 | 1 | 1.81 | 0.10485 | 0.401699567 | AHA_0288,AHA_0379,AHA_2060,AHA_2928 |
| GO:0000097 | sulfur amino acid biosynthetic process | biological_process | 3 | 0 | 3 | 1.16 | 0.10557 | 0.401699567 | AHA_1956,AHA_2807,AHA_3949 |
| GO:0009072 | aromatic amino acid family metabolic process | biological_process | 3 | 2 | 1 | 1.16 | 0.10557 | 0.401699567 | AHA_1654,AHA_2923,AHA_2926 |
| GO:0009144 | purine nucleoside triphosphate metabolic process | biological_process | 9 | 7 | 2 | 5.63 | 0.10688 | 0.402695516 | AHA_0238,AHA_0278,AHA_0608,AHA_3545,AHA_3987,AHA_4115,AHA_4261,AHA_4267,AHA_4285 |
| GO:0033554 | cellular response to stress | biological_process | 9 | 6 | 3 | 5.63 | 0.10688 | 0.402695516 | AHA_0290,AHA_0382,AHA_1153,AHA_1413,AHA_2744,AHA_3004,AHA_3875,AHA_4006,AHA_4008 |
| GO:0009252 | peptidoglycan biosynthetic process | biological_process | 4 | 2 | 2 | 1.83 | 0.10713 | 0.402695516 | AHA_1015,AHA_2130,AHA_2536,AHA_3935 |
| GO:1901564 | organonitrogen compound metabolic process | biological_process | 36 | 24 | 12 | 29.7 | 0.10736 | 0.402695516 | AHA_0238,AHA_0278,AHA_0377,AHA_0378,AHA_0379,AHA_0380,AHA_0481,AHA_0596,AHA_0608,AHA_0803,AHA_1015,AHA_1170,AHA_1270,AHA_1413,AHA_1654,AHA_1834,AHA_1956,AHA_2130,AHA_2405,AHA_2536,AHA_2807,AHA_2923,AHA_2926,AHA_3000,AHA_3004,AHA_3545,AHA_3935,AHA_3949,AHA_3987,AHA_4068,AHA_4070,AHA_4115,AHA_4201,AHA_4261,AHA_4267,AHA_4285 |
| GO:0044281 | small molecule metabolic process | biological_process | 44 | 30 | 14 | 37.3 | 0.1085 | 0.40523331 | AHA_0238,AHA_0278,AHA_0286,AHA_0288,AHA_0290,AHA_0377,AHA_0378,AHA_0379,AHA_0380,AHA_0481,AHA_0596,AHA_0608,AHA_0803,AHA_1008,AHA_1015,AHA_1040,AHA_1170,AHA_1213,AHA_1248,AHA_1270,AHA_1413,AHA_1652,AHA_1654,AHA_1956,AHA_2060,AHA_2405,AHA_2536,AHA_2807,AHA_2923,AHA_2926,AHA_3545,AHA_3601,AHA_3935,AHA_3949,AHA_3987,AHA_4006,AHA_4008,AHA_4068,AHA_4115,AHA_4145,AHA_4201,AHA_4261,AHA_4267,AHA_4285 |
| GO:0006811 | ion transport | biological_process | 12 | 11 | 1 | 8.13 | 0.10911 | 0.40523331 | AHA_0608,AHA_0676,AHA_0913,AHA_1964,AHA_2744,AHA_2812,AHA_2813,AHA_3053,AHA_3728,AHA_4261,AHA_4267,AHA_4285 |
| GO:0004177 | aminopeptidase activity | molecular_function | 2 | 2 | 0 | 0.57 | 0.10919 | 0.40523331 | AHA_1172,AHA_2940 |
| GO:0046174 | polyol catabolic process | biological_process | 2 | 0 | 2 | 0.58 | 0.11068 | 0.409321825 | AHA_4006,AHA_4008 |
| GO:0015171 | amino acid transmembrane transporter activity | molecular_function | 3 | 1 | 2 | 1.19 | 0.11391 | 0.417108729 | AHA_2350,AHA_2806,AHA_3004 |
| GO:0019439 | aromatic compound catabolic process | biological_process | 11 | 10 | 1 | 7.36 | 0.11437 | 0.417108729 | AHA_0278,AHA_0377,AHA_0378,AHA_0379,AHA_0380,AHA_0608,AHA_1008,AHA_3545,AHA_3987,AHA_4115,AHA_4285 |
| GO:0006023 | aminoglycan biosynthetic process | biological_process | 4 | 2 | 2 | 1.88 | 0.11516 | 0.417108729 | AHA_1015,AHA_2130,AHA_2536,AHA_3935 |
| GO:0006024 | glycosaminoglycan biosynthetic process | biological_process | 4 | 2 | 2 | 1.88 | 0.11516 | 0.417108729 | AHA_1015,AHA_2130,AHA_2536,AHA_3935 |
| GO:0042451 | purine nucleoside biosynthetic process | biological_process | 4 | 3 | 1 | 1.88 | 0.11516 | 0.417108729 | AHA_0238,AHA_1413,AHA_4261,AHA_4267 |
| GO:0046129 | purine ribonucleoside biosynthetic process | biological_process | 4 | 3 | 1 | 1.88 | 0.11516 | 0.417108729 | AHA_0238,AHA_1413,AHA_4261,AHA_4267 |
| GO:0009199 | ribonucleoside triphosphate metabolic process | biological_process | 9 | 7 | 2 | 5.73 | 0.11561 | 0.417304589 | AHA_0238,AHA_0278,AHA_0608,AHA_3545,AHA_3987,AHA_4115,AHA_4261,AHA_4267,AHA_4285 |
| GO:0070011 | peptidase activity, acting on L-amino acid peptides | molecular_function | 8 | 7 | 1 | 4.96 | 0.12054 | 0.423382274 | AHA_0612,AHA_0617,AHA_1172,AHA_2163,AHA_2713,AHA_2940,AHA_4114,AHA_4115 |
| GO:0006812 | cation transport | biological_process | 6 | 5 | 1 | 3.42 | 0.12374 | 0.423382274 | AHA_0676,AHA_2744,AHA_3053,AHA_3728,AHA_4261,AHA_4267 |
| GO:0015078 | hydrogen ion transmembrane transporter activity | molecular_function | 3 | 3 | 0 | 1.24 | 0.12449 | 0.423382274 | AHA_2296,AHA_4261,AHA_4267 |
| GO:0016782 | transferase activity, transferring sulfur-containing groups | molecular_function | 2 | 2 | 0 | 0.62 | 0.12515 | 0.423382274 | AHA_3263,AHA_3479 |
| GO:0016783 | sulfurtransferase activity | molecular_function | 2 | 2 | 0 | 0.62 | 0.12515 | 0.423382274 | AHA_3263,AHA_3479 |
| GO:0005623 | cell | cellular_component | 104 | 75 | 29 | 101.16 | 0.1262 | 0.423382274 | AHA_0007,AHA_0008,AHA_0021,AHA_0238,AHA_0262,AHA_0278,AHA_0286,AHA_0289,AHA_0290,AHA_0292,AHA_0295,AHA_0315,AHA_0377,AHA_0379,AHA_0380,AHA_0452,AHA_0596,AHA_0608,AHA_0617,AHA_0625,AHA_0676,AHA_0677,AHA_0724,AHA_0803,AHA_0847,AHA_0857,AHA_0859,AHA_0860,AHA_0913,AHA_0952,AHA_0988,AHA_1008,AHA_1040,AHA_1170,AHA_1213,AHA_1246,AHA_1338,AHA_1391,AHA_1419,AHA_1522,AHA_1595,AHA_1652,AHA_1687,AHA_1713,AHA_1738,AHA_1770,AHA_1805,AHA_1866,AHA_1921,AHA_1964,AHA_2046,AHA_2130,AHA_2305,AHA_2333,AHA_2350,AHA_2405,AHA_2452,AHA_2487,AHA_2490,AHA_2628,AHA_2637,AHA_2715,AHA_2743,AHA_2806,AHA_2809,AHA_2812,AHA_2813,AHA_2835,AHA_2837,AHA_2940,AHA_2976,AHA_2982,AHA_2984,AHA_3004,AHA_3053,AHA_3125,AHA_3263,AHA_3324,AHA_3379,AHA_3477,AHA_3479,AHA_3561,AHA_3604,AHA_3606,AHA_3728,AHA_3740,AHA_3769,AHA_3820,AHA_3821,AHA_3877,AHA_3935,AHA_3949,AHA_3987,AHA_4006,AHA_4008,AHA_4031,AHA_4067,AHA_4068,AHA_4071,AHA_4114,AHA_4115,AHA_4261,AHA_4267,AHA_4285 |
| GO:0044464 | cell part | cellular_component | 104 | 75 | 29 | 101.16 | 0.1262 | 0.423382274 | AHA_0007,AHA_0008,AHA_0021,AHA_0238,AHA_0262,AHA_0278,AHA_0286,AHA_0289,AHA_0290,AHA_0292,AHA_0295,AHA_0315,AHA_0377,AHA_0379,AHA_0380,AHA_0452,AHA_0596,AHA_0608,AHA_0617,AHA_0625,AHA_0676,AHA_0677,AHA_0724,AHA_0803,AHA_0847,AHA_0857,AHA_0859,AHA_0860,AHA_0913,AHA_0952,AHA_0988,AHA_1008,AHA_1040,AHA_1170,AHA_1213,AHA_1246,AHA_1338,AHA_1391,AHA_1419,AHA_1522,AHA_1595,AHA_1652,AHA_1687,AHA_1713,AHA_1738,AHA_1770,AHA_1805,AHA_1866,AHA_1921,AHA_1964,AHA_2046,AHA_2130,AHA_2305,AHA_2333,AHA_2350,AHA_2405,AHA_2452,AHA_2487,AHA_2490,AHA_2628,AHA_2637,AHA_2715,AHA_2743,AHA_2806,AHA_2809,AHA_2812,AHA_2813,AHA_2835,AHA_2837,AHA_2940,AHA_2976,AHA_2982,AHA_2984,AHA_3004,AHA_3053,AHA_3125,AHA_3263,AHA_3324,AHA_3379,AHA_3477,AHA_3479,AHA_3561,AHA_3604,AHA_3606,AHA_3728,AHA_3740,AHA_3769,AHA_3820,AHA_3821,AHA_3877,AHA_3935,AHA_3949,AHA_3987,AHA_4006,AHA_4008,AHA_4031,AHA_4067,AHA_4068,AHA_4071,AHA_4114,AHA_4115,AHA_4261,AHA_4267,AHA_4285 |
| GO:0055082 | cellular chemical homeostasis | biological_process | 2 | 2 | 0 | 0.63 | 0.12683 | 0.423382274 | AHA_1110,AHA_3053 |
| GO:1902600 | hydrogen ion transmembrane transport | biological_process | 2 | 2 | 0 | 0.63 | 0.12683 | 0.423382274 | AHA_4261,AHA_4267 |
| GO:0009126 | purine nucleoside monophosphate metabolic process | biological_process | 9 | 7 | 2 | 5.87 | 0.12942 | 0.423382274 | AHA_0238,AHA_0608,AHA_1413,AHA_3545,AHA_3987,AHA_4115,AHA_4261,AHA_4267,AHA_4285 |
| GO:0009167 | purine ribonucleoside monophosphate metabolic process | biological_process | 9 | 7 | 2 | 5.87 | 0.12942 | 0.423382274 | AHA_0238,AHA_0608,AHA_1413,AHA_3545,AHA_3987,AHA_4115,AHA_4261,AHA_4267,AHA_4285 |
| GO:0044262 | cellular carbohydrate metabolic process | biological_process | 8 | 4 | 4 | 5.05 | 0.12948 | 0.423382274 | AHA_0286,AHA_0288,AHA_0290,AHA_0988,AHA_1213,AHA_1652,AHA_4006,AHA_4008 |
| GO:0044767 | single-organism developmental process | biological_process | 6 | 2 | 4 | 3.47 | 0.12995 | 0.423382274 | AHA_1015,AHA_1921,AHA_2130,AHA_2405,AHA_2536,AHA_3935 |
| GO:0044038 | cell wall macromolecule biosynthetic process | biological_process | 4 | 2 | 2 | 1.97 | 0.13197 | 0.423382274 | AHA_1015,AHA_2130,AHA_2536,AHA_3935 |
| GO:0070589 | cellular component macromolecule biosynthetic process | biological_process | 4 | 2 | 2 | 1.97 | 0.13197 | 0.423382274 | AHA_1015,AHA_2130,AHA_2536,AHA_3935 |
| GO:0005737 | cytoplasm | cellular_component | 45 | 31 | 14 | 39.12 | 0.1337 | 0.423382274 | AHA_0007,AHA_0008,AHA_0289,AHA_0295,AHA_0315,AHA_0377,AHA_0379,AHA_0380,AHA_0596,AHA_0617,AHA_0625,AHA_0677,AHA_0724,AHA_0803,AHA_0859,AHA_0860,AHA_1040,AHA_1170,AHA_1391,AHA_1419,AHA_1522,AHA_1652,AHA_1805,AHA_1921,AHA_1964,AHA_2046,AHA_2305,AHA_2333,AHA_2405,AHA_2490,AHA_2715,AHA_2982,AHA_2984,AHA_3125,AHA_3263,AHA_3877,AHA_3935,AHA_3949,AHA_4006,AHA_4008,AHA_4031,AHA_4068,AHA_4071,AHA_4114,AHA_4115 |
| GO:0005402 | cation:sugar symporter activity | molecular_function | 3 | 1 | 2 | 1.29 | 0.13541 | 0.423382274 | AHA_0286,AHA_1419,AHA_4006 |
| GO:0004029 | aldehyde dehydrogenase (NAD) activity | molecular_function | 1 | 1 | 0 | 0.14 | 0.13653 | 0.423382274 | AHA_1248 |
| GO:0004368 | glycerol-3-phosphate dehydrogenase activity | molecular_function | 1 | 0 | 1 | 0.14 | 0.13653 | 0.423382274 | AHA_1652 |
| GO:0004476 | mannose-6-phosphate isomerase activity | molecular_function | 1 | 0 | 1 | 0.14 | 0.13653 | 0.423382274 | AHA_2338 |
| GO:0004748 | ribonucleoside-diphosphate reductase activity, thioredoxin disulfide as acceptor | molecular_function | 1 | 1 | 0 | 0.14 | 0.13653 | 0.423382274 | AHA_2333 |
| GO:0004792 | thiosulfate sulfurtransferase activity | molecular_function | 1 | 1 | 0 | 0.14 | 0.13653 | 0.423382274 | AHA_3479 |
| GO:0004826 | phenylalanine-tRNA ligase activity | molecular_function | 1 | 0 | 1 | 0.14 | 0.13653 | 0.423382274 | AHA_2537 |
| GO:0015098 | molybdate ion transmembrane transporter activity | molecular_function | 1 | 1 | 0 | 0.14 | 0.13653 | 0.423382274 | AHA_1595 |
| GO:0015128 | gluconate transmembrane transporter activity | molecular_function | 1 | 1 | 0 | 0.14 | 0.13653 | 0.423382274 | AHA_0286 |
| GO:0016411 | acylglycerol O-acyltransferase activity | molecular_function | 1 | 1 | 0 | 0.14 | 0.13653 | 0.423382274 | AHA_3740 |
| GO:0016901 | oxidoreductase activity, acting on the CH-OH group of donors, quinone or similar compound as accepto... | molecular_function | 1 | 0 | 1 | 0.14 | 0.13653 | 0.423382274 | AHA_1652 |
| GO:0022836 | gated channel activity | molecular_function | 1 | 1 | 0 | 0.14 | 0.13653 | 0.423382274 | AHA_3053 |
| GO:0033818 | beta-ketoacyl-acyl-carrier-protein synthase III activity | molecular_function | 1 | 1 | 0 | 0.14 | 0.13653 | 0.423382274 | AHA_1040 |
| GO:0042879 | aldonate transmembrane transporter activity | molecular_function | 1 | 1 | 0 | 0.14 | 0.13653 | 0.423382274 | AHA_0286 |
| GO:0046961 | proton-transporting ATPase activity, rotational mechanism | molecular_function | 1 | 1 | 0 | 0.14 | 0.13653 | 0.423382274 | AHA_4261 |
| GO:0048029 | monosaccharide binding | molecular_function | 1 | 0 | 1 | 0.14 | 0.13653 | 0.423382274 | AHA_2405 |
| GO:0052590 | sn-glycerol-3-phosphate:ubiquinone oxidoreductase activity | molecular_function | 1 | 0 | 1 | 0.14 | 0.13653 | 0.423382274 | AHA_1652 |
| GO:0052591 | sn-glycerol-3-phosphate:ubiquinone-8 oxidoreductase activity | molecular_function | 1 | 0 | 1 | 0.14 | 0.13653 | 0.423382274 | AHA_1652 |
| GO:0060589 | nucleoside-triphosphatase regulator activity | molecular_function | 1 | 1 | 0 | 0.14 | 0.13653 | 0.423382274 | AHA_2984 |
| GO:0071554 | cell wall organization or biogenesis | biological_process | 5 | 3 | 2 | 2.74 | 0.13663 | 0.423382274 | AHA_1015,AHA_2130,AHA_2536,AHA_3935,AHA_4258 |
| GO:0008324 | cation transmembrane transporter activity | molecular_function | 10 | 6 | 4 | 6.78 | 0.13705 | 0.423382274 | AHA_0238,AHA_0286,AHA_1419,AHA_2296,AHA_2743,AHA_3004,AHA_3987,AHA_4006,AHA_4261,AHA_4267 |
| GO:0006083 | acetate metabolic process | biological_process | 1 | 1 | 0 | 0.14 | 0.1376 | 0.423382274 | AHA_4145 |
| GO:0006486 | protein glycosylation | biological_process | 1 | 1 | 0 | 0.14 | 0.1376 | 0.423382274 | AHA_0988 |
| GO:0006885 | regulation of pH | biological_process | 1 | 0 | 1 | 0.14 | 0.1376 | 0.423382274 | AHA_0676 |
| GO:0009446 | putrescine biosynthetic process | biological_process | 1 | 1 | 0 | 0.14 | 0.1376 | 0.423382274 | AHA_1270 |
| GO:0015684 | ferrous iron transport | biological_process | 1 | 1 | 0 | 0.14 | 0.1376 | 0.423382274 | AHA_2744 |
| GO:0015991 | ATP hydrolysis coupled proton transport | biological_process | 1 | 1 | 0 | 0.14 | 0.1376 | 0.423382274 | AHA_4267 |
| GO:0017001 | antibiotic catabolic process | biological_process | 1 | 1 | 0 | 0.14 | 0.1376 | 0.423382274 | AHA_4258 |
| GO:0019662 | non-glycolytic fermentation | biological_process | 1 | 1 | 0 | 0.14 | 0.1376 | 0.423382274 | AHA_4145 |
| GO:0043413 | macromolecule glycosylation | biological_process | 1 | 1 | 0 | 0.14 | 0.1376 | 0.423382274 | AHA_0988 |
| GO:0046176 | aldonic acid catabolic process | biological_process | 1 | 1 | 0 | 0.14 | 0.1376 | 0.423382274 | AHA_0290 |
| GO:0046177 | D-gluconate catabolic process | biological_process | 1 | 1 | 0 | 0.14 | 0.1376 | 0.423382274 | AHA_0290 |
| GO:0048598 | embryonic morphogenesis | biological_process | 1 | 0 | 1 | 0.14 | 0.1376 | 0.423382274 | AHA_1921 |
| GO:0051262 | protein tetramerization | biological_process | 1 | 1 | 0 | 0.14 | 0.1376 | 0.423382274 | AHA_0295 |
| GO:0000096 | sulfur amino acid metabolic process | biological_process | 3 | 0 | 3 | 1.3 | 0.13778 | 0.423382274 | AHA_1956,AHA_2807,AHA_3949 |
| GO:0009141 | nucleoside triphosphate metabolic process | biological_process | 9 | 7 | 2 | 5.97 | 0.1391 | 0.423401709 | AHA_0238,AHA_0278,AHA_0608,AHA_3545,AHA_3987,AHA_4115,AHA_4261,AHA_4267,AHA_4285 |
| GO:0015405 | P-P-bond-hydrolysis-driven transmembrane transporter activity | molecular_function | 6 | 4 | 2 | 3.53 | 0.13914 | 0.423401709 | AHA_0238,AHA_0608,AHA_1595,AHA_1738,AHA_3987,AHA_4261 |
| GO:0008360 | regulation of cell shape | biological_process | 4 | 2 | 2 | 2.02 | 0.14074 | 0.423401709 | AHA_1015,AHA_2130,AHA_2536,AHA_3935 |
| GO:0022603 | regulation of anatomical structure morphogenesis | biological_process | 4 | 2 | 2 | 2.02 | 0.14074 | 0.423401709 | AHA_1015,AHA_2130,AHA_2536,AHA_3935 |
| GO:0022604 | regulation of cell morphogenesis | biological_process | 4 | 2 | 2 | 2.02 | 0.14074 | 0.423401709 | AHA_1015,AHA_2130,AHA_2536,AHA_3935 |
| GO:0005794 | Golgi apparatus | cellular_component | 1 | 0 | 1 | 0.15 | 0.141 | 0.423401709 | AHA_1921 |
| GO:0005971 | ribonucleoside-diphosphate reductase complex | cellular_component | 1 | 1 | 0 | 0.15 | 0.141 | 0.423401709 | AHA_2333 |
| GO:0009331 | glycerol-3-phosphate dehydrogenase complex | cellular_component | 1 | 0 | 1 | 0.15 | 0.141 | 0.423401709 | AHA_1652 |
| GO:0046128 | purine ribonucleoside metabolic process | biological_process | 10 | 8 | 2 | 6.83 | 0.14189 | 0.423666667 | AHA_0238,AHA_0278,AHA_0608,AHA_1413,AHA_3545,AHA_3987,AHA_4115,AHA_4261,AHA_4267,AHA_4285 |
| GO:0032502 | developmental process | biological_process | 6 | 2 | 4 | 3.56 | 0.14283 | 0.423666667 | AHA_1015,AHA_1921,AHA_2130,AHA_2405,AHA_2536,AHA_3935 |
| GO:0000041 | transition metal ion transport | biological_process | 2 | 2 | 0 | 0.67 | 0.1435 | 0.423666667 | AHA_2744,AHA_3728 |
| GO:0007155 | cell adhesion | biological_process | 2 | 1 | 1 | 0.67 | 0.1435 | 0.423666667 | AHA_1921,AHA_3728 |
| GO:0022610 | biological adhesion | biological_process | 2 | 1 | 1 | 0.67 | 0.1435 | 0.423666667 | AHA_1921,AHA_3728 |
| GO:1902582 | single-organism intracellular transport | biological_process | 2 | 2 | 0 | 0.67 | 0.1435 | 0.423666667 | AHA_1738,AHA_3877 |
| GO:0015297 | antiporter activity | molecular_function | 3 | 1 | 2 | 1.34 | 0.14665 | 0.428344432 | AHA_0676,AHA_2806,AHA_3604 |
| GO:0005342 | organic acid transmembrane transporter activity | molecular_function | 4 | 2 | 2 | 2.05 | 0.14671 | 0.428344432 | AHA_0286,AHA_2350,AHA_2806,AHA_3004 |
| GO:0016830 | carbon-carbon lyase activity | molecular_function | 4 | 4 | 0 | 2.05 | 0.14671 | 0.428344432 | AHA_0289,AHA_1270,AHA_2923,AHA_2926 |
| GO:0046943 | carboxylic acid transmembrane transporter activity | molecular_function | 4 | 2 | 2 | 2.05 | 0.14671 | 0.428344432 | AHA_0286,AHA_2350,AHA_2806,AHA_3004 |
| GO:0044391 | ribosomal subunit | cellular_component | 2 | 2 | 0 | 0.69 | 0.1496 | 0.434782259 | AHA_0315,AHA_4031 |
| GO:0050793 | regulation of developmental process | biological_process | 4 | 2 | 2 | 2.07 | 0.14974 | 0.434782259 | AHA_1015,AHA_2130,AHA_2536,AHA_3935 |
| GO:0042278 | purine nucleoside metabolic process | biological_process | 10 | 8 | 2 | 6.93 | 0.15137 | 0.438307637 | AHA_0238,AHA_0278,AHA_0608,AHA_1413,AHA_3545,AHA_3987,AHA_4115,AHA_4261,AHA_4267,AHA_4285 |
| GO:1901565 | organonitrogen compound catabolic process | biological_process | 13 | 10 | 3 | 9.53 | 0.15228 | 0.438619508 | AHA_0278,AHA_0377,AHA_0378,AHA_0379,AHA_0380,AHA_0481,AHA_0608,AHA_3000,AHA_3004,AHA_3545,AHA_3987,AHA_4115,AHA_4285 |
| GO:0015399 | primary active transmembrane transporter activity | molecular_function | 6 | 4 | 2 | 3.63 | 0.15231 | 0.438619508 | AHA_0238,AHA_0608,AHA_1595,AHA_1738,AHA_3987,AHA_4261 |
| GO:0006810 | transport | biological_process | 27 | 23 | 4 | 22.28 | 0.15635 | 0.449026975 | AHA_0022,AHA_0295,AHA_0608,AHA_0676,AHA_0847,AHA_0913,AHA_1246,AHA_1419,AHA_1713,AHA_1738,AHA_1770,AHA_1964,AHA_2637,AHA_2744,AHA_2812,AHA_2813,AHA_3053,AHA_3324,AHA_3545,AHA_3604,AHA_3728,AHA_3820,AHA_3877,AHA_4006,AHA_4261,AHA_4267,AHA_4285 |
| GO:0051179 | localization | biological_process | 29 | 25 | 4 | 24.16 | 0.15697 | 0.449582554 | AHA_0022,AHA_0295,AHA_0608,AHA_0676,AHA_0847,AHA_0913,AHA_1246,AHA_1419,AHA_1713,AHA_1738,AHA_1770,AHA_1964,AHA_2637,AHA_2744,AHA_2812,AHA_2813,AHA_2835,AHA_2837,AHA_3053,AHA_3324,AHA_3545,AHA_3604,AHA_3728,AHA_3820,AHA_3877,AHA_4006,AHA_4261,AHA_4267,AHA_4285 |
| GO:1901606 | alpha-amino acid catabolic process | biological_process | 4 | 4 | 0 | 2.12 | 0.15895 | 0.454019783 | AHA_0377,AHA_0378,AHA_0379,AHA_0380 |
| GO:0006886 | intracellular protein transport | biological_process | 2 | 2 | 0 | 0.72 | 0.16059 | 0.45609686 | AHA_1738,AHA_3877 |
| GO:0008237 | metallopeptidase activity | molecular_function | 4 | 3 | 1 | 2.15 | 0.16504 | 0.45609686 | AHA_0612,AHA_1172,AHA_2163,AHA_2713 |
| GO:0043190 | ATP-binding cassette (ABC) transporter complex | cellular_component | 2 | 1 | 1 | 0.74 | 0.1673 | 0.45609686 | AHA_3324,AHA_3987 |
| GO:0098533 | ATPase dependent transmembrane transport complex | cellular_component | 2 | 1 | 1 | 0.74 | 0.1673 | 0.45609686 | AHA_3324,AHA_3987 |
| GO:0009653 | anatomical structure morphogenesis | biological_process | 5 | 2 | 3 | 2.94 | 0.16749 | 0.45609686 | AHA_1015,AHA_1921,AHA_2130,AHA_2536,AHA_3935 |
| GO:0000902 | cell morphogenesis | biological_process | 4 | 2 | 2 | 2.17 | 0.16837 | 0.45609686 | AHA_1015,AHA_2130,AHA_2536,AHA_3935 |
| GO:0019538 | protein metabolic process | biological_process | 19 | 16 | 3 | 15.11 | 0.16945 | 0.45609686 | AHA_0315,AHA_0612,AHA_0617,AHA_0724,AHA_0859,AHA_0860,AHA_0988,AHA_1172,AHA_2163,AHA_2405,AHA_2490,AHA_2713,AHA_2982,AHA_2983,AHA_2984,AHA_4031,AHA_4068,AHA_4071,AHA_4114 |
| GO:0051234 | establishment of localization | biological_process | 27 | 23 | 4 | 22.57 | 0.17394 | 0.45609686 | AHA_0022,AHA_0295,AHA_0608,AHA_0676,AHA_0847,AHA_0913,AHA_1246,AHA_1419,AHA_1713,AHA_1738,AHA_1770,AHA_1964,AHA_2637,AHA_2744,AHA_2812,AHA_2813,AHA_3053,AHA_3324,AHA_3545,AHA_3604,AHA_3728,AHA_3820,AHA_3877,AHA_4006,AHA_4261,AHA_4267,AHA_4285 |
| GO:0044459 | plasma membrane part | cellular_component | 6 | 3 | 3 | 3.8 | 0.1769 | 0.45609686 | AHA_0286,AHA_0952,AHA_2350,AHA_2812,AHA_3324,AHA_3987 |
| GO:0003849 | 3-deoxy-7-phosphoheptulonate synthase activity | molecular_function | 1 | 0 | 1 | 0.19 | 0.17779 | 0.45609686 | AHA_1654 |
| GO:0008199 | ferric iron binding | molecular_function | 1 | 1 | 0 | 0.19 | 0.17779 | 0.45609686 | AHA_1110 |
| GO:0008409 | 5'-3' exonuclease activity | molecular_function | 1 | 0 | 1 | 0.19 | 0.17779 | 0.45609686 | AHA_1153 |
| GO:0008800 | beta-lactamase activity | molecular_function | 1 | 1 | 0 | 0.19 | 0.17779 | 0.45609686 | AHA_4258 |
| GO:0008965 | phosphoenolpyruvate-protein phosphotransferase activity | molecular_function | 1 | 0 | 1 | 0.19 | 0.17779 | 0.45609686 | AHA_4006 |
| GO:0015095 | magnesium ion transmembrane transporter activity | molecular_function | 1 | 0 | 1 | 0.19 | 0.17779 | 0.45609686 | AHA_0238 |
| GO:0016211 | ammonia ligase activity | molecular_function | 1 | 1 | 0 | 0.19 | 0.17779 | 0.45609686 | AHA_4068 |
| GO:0016728 | oxidoreductase activity, acting on CH or CH2 groups, disulfide as acceptor | molecular_function | 1 | 1 | 0 | 0.19 | 0.17779 | 0.45609686 | AHA_2333 |
| GO:0016748 | succinyltransferase activity | molecular_function | 1 | 1 | 0 | 0.19 | 0.17779 | 0.45609686 | AHA_1170 |
| GO:0016880 | acid-ammonia (or amide) ligase activity | molecular_function | 1 | 1 | 0 | 0.19 | 0.17779 | 0.45609686 | AHA_4068 |
| GO:0036442 | hydrogen-exporting ATPase activity | molecular_function | 1 | 1 | 0 | 0.19 | 0.17779 | 0.45609686 | AHA_4261 |
| GO:0032989 | cellular component morphogenesis | biological_process | 4 | 2 | 2 | 2.21 | 0.17798 | 0.45609686 | AHA_1015,AHA_2130,AHA_2536,AHA_3935 |
| GO:0046907 | intracellular transport | biological_process | 2 | 2 | 0 | 0.77 | 0.17803 | 0.45609686 | AHA_1738,AHA_3877 |
| GO:0006072 | glycerol-3-phosphate metabolic process | biological_process | 1 | 0 | 1 | 0.19 | 0.17915 | 0.45609686 | AHA_1652 |
| GO:0006167 | AMP biosynthetic process | biological_process | 1 | 1 | 0 | 0.19 | 0.17915 | 0.45609686 | AHA_1413 |
| GO:0006474 | N-terminal protein amino acid acetylation | biological_process | 1 | 0 | 1 | 0.19 | 0.17915 | 0.45609686 | AHA_0724 |
| GO:0006528 | asparagine metabolic process | biological_process | 1 | 1 | 0 | 0.19 | 0.17915 | 0.45609686 | AHA_4068 |
| GO:0006591 | ornithine metabolic process | biological_process | 1 | 1 | 0 | 0.19 | 0.17915 | 0.45609686 | AHA_1270 |
| GO:0006596 | polyamine biosynthetic process | biological_process | 1 | 1 | 0 | 0.19 | 0.17915 | 0.45609686 | AHA_1270 |
| GO:0006805 | xenobiotic metabolic process | biological_process | 1 | 1 | 0 | 0.19 | 0.17915 | 0.45609686 | AHA_0735 |
| GO:0006855 | drug transmembrane transport | biological_process | 1 | 1 | 0 | 0.19 | 0.17915 | 0.45609686 | AHA_1713 |
| GO:0007166 | cell surface receptor signaling pathway | biological_process | 1 | 0 | 1 | 0.19 | 0.17915 | 0.45609686 | AHA_2046 |
| GO:0009100 | glycoprotein metabolic process | biological_process | 1 | 1 | 0 | 0.19 | 0.17915 | 0.45609686 | AHA_0988 |
| GO:0009101 | glycoprotein biosynthetic process | biological_process | 1 | 1 | 0 | 0.19 | 0.17915 | 0.45609686 | AHA_0988 |
| GO:0016024 | CDP-diacylglycerol biosynthetic process | biological_process | 1 | 1 | 0 | 0.19 | 0.17915 | 0.45609686 | AHA_3740 |
| GO:0031365 | N-terminal protein amino acid modification | biological_process | 1 | 0 | 1 | 0.19 | 0.17915 | 0.45609686 | AHA_0724 |
| GO:0035999 | tetrahydrofolate interconversion | biological_process | 1 | 0 | 1 | 0.19 | 0.17915 | 0.45609686 | AHA_3949 |
| GO:0042182 | ketone catabolic process | biological_process | 1 | 0 | 1 | 0.19 | 0.17915 | 0.45609686 | AHA_4008 |
| GO:0044702 | single organism reproductive process | biological_process | 1 | 0 | 1 | 0.19 | 0.17915 | 0.45609686 | AHA_2405 |
| GO:0046033 | AMP metabolic process | biological_process | 1 | 1 | 0 | 0.19 | 0.17915 | 0.45609686 | AHA_1413 |
| GO:0046341 | CDP-diacylglycerol metabolic process | biological_process | 1 | 1 | 0 | 0.19 | 0.17915 | 0.45609686 | AHA_3740 |
| GO:0046349 | amino sugar biosynthetic process | biological_process | 1 | 1 | 0 | 0.19 | 0.17915 | 0.45609686 | AHA_3935 |
| GO:0048513 | organ development | biological_process | 1 | 0 | 1 | 0.19 | 0.17915 | 0.45609686 | AHA_2405 |
| GO:0052646 | alditol phosphate metabolic process | biological_process | 1 | 0 | 1 | 0.19 | 0.17915 | 0.45609686 | AHA_1652 |
| GO:0055067 | monovalent inorganic cation homeostasis | biological_process | 1 | 0 | 1 | 0.19 | 0.17915 | 0.45609686 | AHA_0676 |
| GO:0071466 | cellular response to xenobiotic stimulus | biological_process | 1 | 1 | 0 | 0.19 | 0.17915 | 0.45609686 | AHA_0735 |
| GO:0009150 | purine ribonucleotide metabolic process | biological_process | 10 | 8 | 2 | 7.22 | 0.1817 | 0.460120288 | AHA_0238,AHA_0278,AHA_0608,AHA_1413,AHA_3545,AHA_3987,AHA_4115,AHA_4261,AHA_4267,AHA_4285 |
| GO:0015295 | solute:hydrogen symporter activity | molecular_function | 3 | 1 | 2 | 1.48 | 0.18204 | 0.460120288 | AHA_1419,AHA_3004,AHA_4006 |
| GO:0016765 | transferase activity, transferring alkyl or aryl (other than methyl) groups | molecular_function | 3 | 2 | 1 | 1.48 | 0.18204 | 0.460120288 | AHA_1654,AHA_3935,AHA_4070 |
| GO:0033177 | proton-transporting two-sector ATPase complex, proton-transporting domain | cellular_component | 1 | 1 | 0 | 0.2 | 0.1835 | 0.460812796 | AHA_4267 |
| GO:0045263 | proton-transporting ATP synthase complex, coupling factor F(o) | cellular_component | 1 | 1 | 0 | 0.2 | 0.1835 | 0.460812796 | AHA_4267 |
| GO:1901575 | organic substance catabolic process | biological_process | 20 | 15 | 5 | 16.22 | 0.18369 | 0.460812796 | AHA_0278,AHA_0290,AHA_0377,AHA_0378,AHA_0379,AHA_0380,AHA_0481,AHA_0608,AHA_1008,AHA_1248,AHA_3000,AHA_3004,AHA_3545,AHA_3987,AHA_4006,AHA_4008,AHA_4114,AHA_4115,AHA_4145,AHA_4285 |
| GO:0008514 | organic anion transmembrane transporter activity | molecular_function | 4 | 2 | 2 | 2.24 | 0.18414 | 0.460812796 | AHA_0286,AHA_2350,AHA_2806,AHA_3004 |
| GO:0044422 | organelle part | cellular_component | 6 | 4 | 2 | 3.85 | 0.1845 | 0.460812796 | AHA_0315,AHA_1921,AHA_2046,AHA_2835,AHA_2837,AHA_4031 |
| GO:1902495 | transmembrane transporter complex | cellular_component | 2 | 1 | 1 | 0.79 | 0.1853 | 0.461716785 | AHA_3324,AHA_3987 |
| GO:0030001 | metal ion transport | biological_process | 4 | 3 | 1 | 2.26 | 0.18777 | 0.4664384 | AHA_0676,AHA_2744,AHA_3053,AHA_3728 |
| GO:0042626 | ATPase activity, coupled to transmembrane movement of substances | molecular_function | 5 | 3 | 2 | 3.06 | 0.18808 | 0.4664384 | AHA_0238,AHA_0608,AHA_1595,AHA_3987,AHA_4261 |
| GO:0008233 | peptidase activity | molecular_function | 8 | 7 | 1 | 5.59 | 0.19275 | 0.476073084 | AHA_0612,AHA_0617,AHA_1172,AHA_2163,AHA_2713,AHA_2940,AHA_4114,AHA_4115 |
| GO:0016861 | intramolecular oxidoreductase activity, interconverting aldoses and ketoses | molecular_function | 2 | 1 | 1 | 0.81 | 0.19332 | 0.476073084 | AHA_2338,AHA_2926 |
| GO:0033218 | amide binding | molecular_function | 2 | 2 | 0 | 0.81 | 0.19332 | 0.476073084 | AHA_3324,AHA_4258 |
| GO:0032501 | multicellular organismal process | biological_process | 2 | 0 | 2 | 0.82 | 0.19575 | 0.479399213 | AHA_1921,AHA_2405 |
| GO:0044707 | single-multicellular organism process | biological_process | 2 | 0 | 2 | 0.82 | 0.19575 | 0.479399213 | AHA_1921,AHA_2405 |
| GO:0043492 | ATPase activity, coupled to movement of substances | molecular_function | 5 | 3 | 2 | 3.1 | 0.19648 | 0.479399213 | AHA_0238,AHA_0608,AHA_1595,AHA_3987,AHA_4261 |
| GO:0044267 | cellular protein metabolic process | biological_process | 13 | 11 | 2 | 10.01 | 0.19649 | 0.479399213 | AHA_0315,AHA_0724,AHA_0859,AHA_0860,AHA_0988,AHA_2405,AHA_2490,AHA_2982,AHA_2983,AHA_2984,AHA_4031,AHA_4068,AHA_4114 |
| GO:0009152 | purine ribonucleotide biosynthetic process | biological_process | 4 | 3 | 1 | 2.31 | 0.19774 | 0.481334781 | AHA_0238,AHA_1413,AHA_4261,AHA_4267 |
| GO:0009161 | ribonucleoside monophosphate metabolic process | biological_process | 9 | 7 | 2 | 6.5 | 0.19864 | 0.482411429 | AHA_0238,AHA_0608,AHA_1413,AHA_3545,AHA_3987,AHA_4115,AHA_4261,AHA_4267,AHA_4285 |
| GO:0005887 | integral component of plasma membrane | cellular_component | 4 | 2 | 2 | 2.32 | 0.1997 | 0.483870805 | AHA_0286,AHA_0952,AHA_2350,AHA_2812 |
| GO:0009156 | ribonucleoside monophosphate biosynthetic process | biological_process | 4 | 3 | 1 | 2.36 | 0.20786 | 0.4991671 | AHA_0238,AHA_1413,AHA_4261,AHA_4267 |
| GO:0009056 | catabolic process | biological_process | 21 | 16 | 5 | 17.47 | 0.20866 | 0.4991671 | AHA_0278,AHA_0290,AHA_0377,AHA_0378,AHA_0379,AHA_0380,AHA_0481,AHA_0608,AHA_1008,AHA_1248,AHA_3000,AHA_3004,AHA_3545,AHA_3987,AHA_4006,AHA_4008,AHA_4114,AHA_4115,AHA_4145,AHA_4258,AHA_4285 |
| GO:0044238 | primary metabolic process | biological_process | 83 | 52 | 31 | 78.26 | 0.21301 | 0.4991671 | AHA_0238,AHA_0262,AHA_0278,AHA_0286,AHA_0288,AHA_0290,AHA_0315,AHA_0377,AHA_0378,AHA_0379,AHA_0380,AHA_0481,AHA_0596,AHA_0608,AHA_0612,AHA_0617,AHA_0677,AHA_0724,AHA_0859,AHA_0860,AHA_0988,AHA_1008,AHA_1015,AHA_1040,AHA_1153,AHA_1170,AHA_1172,AHA_1201,AHA_1213,AHA_1270,AHA_1338,AHA_1339,AHA_1413,AHA_1522,AHA_1652,AHA_1654,AHA_1805,AHA_1956,AHA_1960,AHA_2060,AHA_2163,AHA_2305,AHA_2333,AHA_2338,AHA_2405,AHA_2490,AHA_2536,AHA_2538,AHA_2561,AHA_2713,AHA_2807,AHA_2851,AHA_2923,AHA_2926,AHA_2982,AHA_2983,AHA_2984,AHA_3000,AHA_3415,AHA_3545,AHA_3561,AHA_3601,AHA_3606,AHA_3740,AHA_3821,AHA_3874,AHA_3875,AHA_3935,AHA_3949,AHA_3987,AHA_4006,AHA_4008,AHA_4031,AHA_4067,AHA_4068,AHA_4071,AHA_4114,AHA_4115,AHA_4145,AHA_4201,AHA_4261,AHA_4267,AHA_4285 |
| GO:0042493 | response to drug | biological_process | 2 | 2 | 0 | 0.87 | 0.21368 | 0.4991671 | AHA_0292,AHA_1713 |
| GO:0009123 | nucleoside monophosphate metabolic process | biological_process | 9 | 7 | 2 | 6.64 | 0.21655 | 0.4991671 | AHA_0238,AHA_0608,AHA_1413,AHA_3545,AHA_3987,AHA_4115,AHA_4261,AHA_4267,AHA_4285 |
| GO:0004129 | cytochrome-c oxidase activity | molecular_function | 1 | 1 | 0 | 0.24 | 0.21709 | 0.4991671 | AHA_2296 |
| GO:0004315 | 3-oxoacyl-[acyl-carrier-protein] synthase activity | molecular_function | 1 | 1 | 0 | 0.24 | 0.21709 | 0.4991671 | AHA_1040 |
| GO:0016675 | oxidoreductase activity, acting on a heme group of donors | molecular_function | 1 | 1 | 0 | 0.24 | 0.21709 | 0.4991671 | AHA_2296 |
| GO:0016676 | oxidoreductase activity, acting on a heme group of donors, oxygen as acceptor | molecular_function | 1 | 1 | 0 | 0.24 | 0.21709 | 0.4991671 | AHA_2296 |
| GO:0016722 | oxidoreductase activity, oxidizing metal ions | molecular_function | 1 | 1 | 0 | 0.24 | 0.21709 | 0.4991671 | AHA_1110 |
| GO:0042277 | peptide binding | molecular_function | 1 | 1 | 0 | 0.24 | 0.21709 | 0.4991671 | AHA_3324 |
| GO:0044769 | ATPase activity, coupled to transmembrane movement of ions, rotational mechanism | molecular_function | 1 | 1 | 0 | 0.24 | 0.21709 | 0.4991671 | AHA_4261 |
| GO:0046933 | proton-transporting ATP synthase activity, rotational mechanism | molecular_function | 1 | 1 | 0 | 0.24 | 0.21709 | 0.4991671 | AHA_4261 |
| GO:0047429 | nucleoside-triphosphate diphosphatase activity | molecular_function | 1 | 1 | 0 | 0.24 | 0.21709 | 0.4991671 | AHA_3875 |
| GO:0003006 | developmental process involved in reproduction | biological_process | 1 | 0 | 1 | 0.24 | 0.21872 | 0.4991671 | AHA_2405 |
| GO:0006105 | succinate metabolic process | biological_process | 1 | 1 | 0 | 0.24 | 0.21872 | 0.4991671 | AHA_4145 |
| GO:0006113 | fermentation | biological_process | 1 | 1 | 0 | 0.24 | 0.21872 | 0.4991671 | AHA_4145 |
| GO:0006826 | iron ion transport | biological_process | 1 | 1 | 0 | 0.24 | 0.21872 | 0.4991671 | AHA_2744 |
| GO:0006829 | zinc ion transport | biological_process | 1 | 1 | 0 | 0.24 | 0.21872 | 0.4991671 | AHA_3728 |
| GO:0009410 | response to xenobiotic stimulus | biological_process | 1 | 1 | 0 | 0.24 | 0.21872 | 0.4991671 | AHA_0735 |
| GO:0009790 | embryo development | biological_process | 1 | 0 | 1 | 0.24 | 0.21872 | 0.4991671 | AHA_1921 |
| GO:0022414 | reproductive process | biological_process | 1 | 0 | 1 | 0.24 | 0.21872 | 0.4991671 | AHA_2405 |
| GO:0043952 | protein transport by the Sec complex | biological_process | 1 | 1 | 0 | 0.24 | 0.21872 | 0.4991671 | AHA_1738 |
| GO:0048731 | system development | biological_process | 1 | 0 | 1 | 0.24 | 0.21872 | 0.4991671 | AHA_2405 |
| GO:0070085 | glycosylation | biological_process | 1 | 1 | 0 | 0.24 | 0.21872 | 0.4991671 | AHA_0988 |
| GO:0070838 | divalent metal ion transport | biological_process | 1 | 1 | 0 | 0.24 | 0.21872 | 0.4991671 | AHA_2744 |
| GO:0044444 | cytoplasmic part | cellular_component | 11 | 6 | 5 | 8.45 | 0.2188 | 0.4991671 | AHA_0315,AHA_1652,AHA_1921,AHA_2046,AHA_2333,AHA_2715,AHA_3949,AHA_4008,AHA_4031,AHA_4114,AHA_4115 |
| GO:0006163 | purine nucleotide metabolic process | biological_process | 10 | 8 | 2 | 7.56 | 0.22036 | 0.501640259 | AHA_0238,AHA_0278,AHA_0608,AHA_1413,AHA_3545,AHA_3987,AHA_4115,AHA_4261,AHA_4267,AHA_4285 |
| GO:1901566 | organonitrogen compound biosynthetic process | biological_process | 20 | 13 | 7 | 16.7 | 0.22156 | 0.503285 | AHA_0238,AHA_0596,AHA_0803,AHA_1015,AHA_1170,AHA_1270,AHA_1413,AHA_1654,AHA_1956,AHA_2130,AHA_2536,AHA_2807,AHA_2923,AHA_2926,AHA_3935,AHA_3949,AHA_4068,AHA_4201,AHA_4261,AHA_4267 |
| GO:0009425 | bacterial-type flagellum basal body | cellular_component | 2 | 2 | 0 | 0.89 | 0.2221 | 0.503426667 | AHA_2835,AHA_2837 |
| GO:0005789 | endoplasmic reticulum membrane | cellular_component | 1 | 0 | 1 | 0.25 | 0.2239 | 0.504253419 | AHA_2046 |
| GO:0042175 | nuclear outer membrane-endoplasmic reticulum membrane network | cellular_component | 1 | 0 | 1 | 0.25 | 0.2239 | 0.504253419 | AHA_2046 |
| GO:0045261 | proton-transporting ATP synthase complex, catalytic core F(1) | cellular_component | 1 | 1 | 0 | 0.25 | 0.2239 | 0.504253419 | AHA_4261 |
| GO:0006164 | purine nucleotide biosynthetic process | biological_process | 4 | 3 | 1 | 2.45 | 0.22855 | 0.513477404 | AHA_0238,AHA_1413,AHA_4261,AHA_4267 |
| GO:0048038 | quinone binding | molecular_function | 2 | 1 | 1 | 0.91 | 0.22897 | 0.513477404 | AHA_1770,AHA_1834 |
| GO:0005829 | cytosol | cellular_component | 4 | 2 | 2 | 2.47 | 0.2315 | 0.516437717 | AHA_3949,AHA_4008,AHA_4114,AHA_4115 |
| GO:0006818 | hydrogen transport | biological_process | 2 | 2 | 0 | 0.91 | 0.23176 | 0.516437717 | AHA_4261,AHA_4267 |
| GO:0015992 | proton transport | biological_process | 2 | 2 | 0 | 0.91 | 0.23176 | 0.516437717 | AHA_4261,AHA_4267 |
| GO:0030288 | outer membrane-bounded periplasmic space | cellular_component | 5 | 4 | 1 | 3.31 | 0.2336 | 0.519439662 | AHA_1595,AHA_2452,AHA_2835,AHA_3324,AHA_3769 |
| GO:0004175 | endopeptidase activity | molecular_function | 4 | 3 | 1 | 2.48 | 0.23472 | 0.520831326 | AHA_0612,AHA_0617,AHA_2713,AHA_4114 |
| GO:0044283 | small molecule biosynthetic process | biological_process | 15 | 10 | 5 | 12.22 | 0.2354 | 0.521242857 | AHA_0596,AHA_0803,AHA_1015,AHA_1040,AHA_1170,AHA_1654,AHA_1956,AHA_2536,AHA_2807,AHA_2923,AHA_2926,AHA_3601,AHA_3949,AHA_4068,AHA_4201 |
| GO:0009063 | cellular amino acid catabolic process | biological_process | 4 | 4 | 0 | 2.5 | 0.23908 | 0.526075825 | AHA_0377,AHA_0378,AHA_0379,AHA_0380 |
| GO:0009124 | nucleoside monophosphate biosynthetic process | biological_process | 4 | 3 | 1 | 2.5 | 0.23908 | 0.526075825 | AHA_0238,AHA_1413,AHA_4261,AHA_4267 |
| GO:0015672 | monovalent inorganic cation transport | biological_process | 4 | 3 | 1 | 2.5 | 0.23908 | 0.526075825 | AHA_0676,AHA_3053,AHA_4261,AHA_4267 |
| GO:0016820 | hydrolase activity, acting on acid anhydrides, catalyzing transmembrane movement of substances | molecular_function | 5 | 3 | 2 | 3.34 | 0.24031 | 0.52765738 | AHA_0238,AHA_0608,AHA_1595,AHA_3987,AHA_4261 |
| GO:0031090 | organelle membrane | cellular_component | 2 | 0 | 2 | 0.94 | 0.2408 | 0.52765738 | AHA_1921,AHA_2046 |
| GO:0009119 | ribonucleoside metabolic process | biological_process | 10 | 8 | 2 | 7.75 | 0.24384 | 0.53195878 | AHA_0238,AHA_0278,AHA_0608,AHA_1413,AHA_3545,AHA_3987,AHA_4115,AHA_4261,AHA_4267,AHA_4285 |
| GO:0051119 | sugar transmembrane transporter activity | molecular_function | 3 | 1 | 2 | 1.72 | 0.24519 | 0.53195878 | AHA_0286,AHA_1419,AHA_4006 |
| GO:0016831 | carboxy-lyase activity | molecular_function | 2 | 2 | 0 | 0.95 | 0.24698 | 0.53195878 | AHA_1270,AHA_2926 |
| GO:0051128 | regulation of cellular component organization | biological_process | 4 | 2 | 2 | 2.55 | 0.24973 | 0.53195878 | AHA_1015,AHA_2130,AHA_2536,AHA_3935 |
| GO:0042558 | pteridine-containing compound metabolic process | biological_process | 2 | 1 | 1 | 0.96 | 0.24993 | 0.53195878 | AHA_3949,AHA_4070 |
| GO:0008235 | metalloexopeptidase activity | molecular_function | 1 | 1 | 0 | 0.29 | 0.25453 | 0.53195878 | AHA_1172 |
| GO:0015002 | heme-copper terminal oxidase activity | molecular_function | 1 | 1 | 0 | 0.29 | 0.25453 | 0.53195878 | AHA_2296 |
| GO:0016813 | hydrolase activity, acting on carbon-nitrogen (but not peptide) bonds, in linear amidines | molecular_function | 1 | 1 | 0 | 0.29 | 0.25453 | 0.53195878 | AHA_0378 |
| GO:0019203 | carbohydrate phosphatase activity | molecular_function | 1 | 1 | 0 | 0.29 | 0.25453 | 0.53195878 | AHA_3603 |
| GO:0042954 | lipoprotein transporter activity | molecular_function | 1 | 1 | 0 | 0.29 | 0.25453 | 0.53195878 | AHA_1866 |
| GO:0050308 | sugar-phosphatase activity | molecular_function | 1 | 1 | 0 | 0.29 | 0.25453 | 0.53195878 | AHA_3603 |
| GO:0070283 | radical SAM enzyme activity | molecular_function | 1 | 1 | 0 | 0.29 | 0.25453 | 0.53195878 | AHA_3263 |
| GO:0006473 | protein acetylation | biological_process | 1 | 0 | 1 | 0.29 | 0.25639 | 0.53195878 | AHA_0724 |
| GO:0006879 | cellular iron ion homeostasis | biological_process | 1 | 1 | 0 | 0.29 | 0.25639 | 0.53195878 | AHA_1110 |
| GO:0008272 | sulfate transport | biological_process | 1 | 1 | 0 | 0.29 | 0.25639 | 0.53195878 | AHA_0608 |
| GO:0008614 | pyridoxine metabolic process | biological_process | 1 | 1 | 0 | 0.29 | 0.25639 | 0.53195878 | AHA_0803 |
| GO:0008615 | pyridoxine biosynthetic process | biological_process | 1 | 1 | 0 | 0.29 | 0.25639 | 0.53195878 | AHA_0803 |
| GO:0009877 | nodulation | biological_process | 1 | 1 | 0 | 0.29 | 0.25639 | 0.53195878 | AHA_0452 |
| GO:0042594 | response to starvation | biological_process | 1 | 0 | 1 | 0.29 | 0.25639 | 0.53195878 | AHA_2405 |
| GO:0042773 | ATP synthesis coupled electron transport | biological_process | 1 | 1 | 0 | 0.29 | 0.25639 | 0.53195878 | AHA_1770 |
| GO:0042816 | vitamin B6 metabolic process | biological_process | 1 | 1 | 0 | 0.29 | 0.25639 | 0.53195878 | AHA_0803 |
| GO:0042819 | vitamin B6 biosynthetic process | biological_process | 1 | 1 | 0 | 0.29 | 0.25639 | 0.53195878 | AHA_0803 |
| GO:0043543 | protein acylation | biological_process | 1 | 0 | 1 | 0.29 | 0.25639 | 0.53195878 | AHA_0724 |
| GO:0044257 | cellular protein catabolic process | biological_process | 1 | 1 | 0 | 0.29 | 0.25639 | 0.53195878 | AHA_4114 |
| GO:0051603 | proteolysis involved in cellular protein catabolic process | biological_process | 1 | 1 | 0 | 0.29 | 0.25639 | 0.53195878 | AHA_4114 |
| GO:0065002 | intracellular protein transmembrane transport | biological_process | 1 | 1 | 0 | 0.29 | 0.25639 | 0.53195878 | AHA_1738 |
| GO:0072511 | divalent inorganic cation transport | biological_process | 1 | 1 | 0 | 0.29 | 0.25639 | 0.53195878 | AHA_2744 |
| GO:0022890 | inorganic cation transmembrane transporter activity | molecular_function | 6 | 4 | 2 | 4.3 | 0.25771 | 0.533647033 | AHA_0238,AHA_2296,AHA_2743,AHA_3987,AHA_4261,AHA_4267 |
| GO:0008238 | exopeptidase activity | molecular_function | 3 | 3 | 0 | 1.77 | 0.25824 | 0.533696 | AHA_0617,AHA_1172,AHA_2940 |
| GO:0044036 | cell wall macromolecule metabolic process | biological_process | 4 | 2 | 2 | 2.6 | 0.26048 | 0.536164338 | AHA_1015,AHA_2130,AHA_2536,AHA_3935 |
| GO:0009259 | ribonucleotide metabolic process | biological_process | 10 | 8 | 2 | 7.89 | 0.26203 | 0.536164338 | AHA_0238,AHA_0278,AHA_0608,AHA_1413,AHA_3545,AHA_3987,AHA_4115,AHA_4261,AHA_4267,AHA_4285 |
| GO:0005743 | mitochondrial inner membrane | cellular_component | 1 | 0 | 1 | 0.3 | 0.2624 | 0.536164338 | AHA_1921 |
| GO:0033178 | proton-transporting two-sector ATPase complex, catalytic domain | cellular_component | 1 | 1 | 0 | 0.3 | 0.2624 | 0.536164338 | AHA_4261 |
| GO:0044432 | endoplasmic reticulum part | cellular_component | 1 | 0 | 1 | 0.3 | 0.2624 | 0.536164338 | AHA_2046 |
| GO:0016853 | isomerase activity | molecular_function | 7 | 3 | 4 | 5.2 | 0.26249 | 0.536164338 | AHA_0262,AHA_1015,AHA_2060,AHA_2338,AHA_2536,AHA_2851,AHA_2926 |
| GO:0006820 | anion transport | biological_process | 5 | 5 | 0 | 3.47 | 0.26388 | 0.536164338 | AHA_0608,AHA_0913,AHA_2812,AHA_2813,AHA_4285 |
| GO:0031226 | intrinsic component of plasma membrane | cellular_component | 4 | 2 | 2 | 2.62 | 0.2645 | 0.536164338 | AHA_0286,AHA_0952,AHA_2350,AHA_2812 |
| GO:0044446 | intracellular organelle part | cellular_component | 4 | 2 | 2 | 2.62 | 0.2645 | 0.536164338 | AHA_0315,AHA_1921,AHA_2046,AHA_4031 |
| GO:0004222 | metalloendopeptidase activity | molecular_function | 2 | 1 | 1 | 1 | 0.26503 | 0.536164338 | AHA_0612,AHA_2713 |
| GO:0046915 | transition metal ion transmembrane transporter activity | molecular_function | 2 | 1 | 1 | 1 | 0.26503 | 0.536164338 | AHA_2743,AHA_3987 |
| GO:0015294 | solute:cation symporter activity | molecular_function | 4 | 2 | 2 | 2.63 | 0.26655 | 0.538206322 | AHA_0286,AHA_1419,AHA_3004,AHA_4006 |
| GO:0034613 | cellular protein localization | biological_process | 2 | 2 | 0 | 1.01 | 0.26814 | 0.539350305 | AHA_1738,AHA_3877 |
| GO:0070727 | cellular macromolecule localization | biological_process | 2 | 2 | 0 | 1.01 | 0.26814 | 0.539350305 | AHA_1738,AHA_3877 |
| GO:0016485 | protein processing | biological_process | 7 | 6 | 1 | 5.25 | 0.26899 | 0.540029448 | AHA_0612,AHA_0617,AHA_1172,AHA_2163,AHA_2713,AHA_4071,AHA_4114 |
| GO:0019693 | ribose phosphate metabolic process | biological_process | 10 | 8 | 2 | 7.99 | 0.27441 | 0.549863384 | AHA_0238,AHA_0278,AHA_0608,AHA_1413,AHA_3545,AHA_3987,AHA_4115,AHA_4261,AHA_4267,AHA_4285 |
| GO:0044248 | cellular catabolic process | biological_process | 15 | 13 | 2 | 12.66 | 0.27942 | 0.55884 | AHA_0278,AHA_0290,AHA_0377,AHA_0378,AHA_0379,AHA_0380,AHA_0608,AHA_1008,AHA_3545,AHA_3987,AHA_4008,AHA_4114,AHA_4115,AHA_4258,AHA_4285 |
| GO:0000270 | peptidoglycan metabolic process | biological_process | 4 | 2 | 2 | 2.7 | 0.28225 | 0.563430871 | AHA_1015,AHA_2130,AHA_2536,AHA_3935 |
| GO:0016854 | racemase and epimerase activity | molecular_function | 2 | 1 | 1 | 1.05 | 0.28309 | 0.564039433 | AHA_1015,AHA_2536 |
| GO:0005506 | iron ion binding | molecular_function | 4 | 3 | 1 | 2.72 | 0.2882 | 0.568288125 | AHA_1110,AHA_1948,AHA_2405,AHA_3601 |
| GO:0008028 | monocarboxylic acid transmembrane transporter activity | molecular_function | 1 | 1 | 0 | 0.33 | 0.29019 | 0.568288125 | AHA_0286 |
| GO:0016832 | aldehyde-lyase activity | molecular_function | 1 | 1 | 0 | 0.33 | 0.29019 | 0.568288125 | AHA_0289 |
| GO:0046857 | oxidoreductase activity, acting on other nitrogenous compounds as donors, with NAD or NADP as accept... | molecular_function | 1 | 1 | 0 | 0.33 | 0.29019 | 0.568288125 | AHA_0735 |
| GO:0009166 | nucleotide catabolic process | biological_process | 7 | 6 | 1 | 5.39 | 0.2921 | 0.568288125 | AHA_0278,AHA_0608,AHA_1008,AHA_3545,AHA_3987,AHA_4115,AHA_4285 |
| GO:0015988 | energy coupled proton transmembrane transport, against electrochemical gradient | biological_process | 1 | 1 | 0 | 0.34 | 0.29226 | 0.568288125 | AHA_4267 |
| GO:0016999 | antibiotic metabolic process | biological_process | 1 | 1 | 0 | 0.34 | 0.29226 | 0.568288125 | AHA_4258 |
| GO:0017144 | drug metabolic process | biological_process | 1 | 1 | 0 | 0.34 | 0.29226 | 0.568288125 | AHA_4258 |
| GO:0031667 | response to nutrient levels | biological_process | 1 | 0 | 1 | 0.34 | 0.29226 | 0.568288125 | AHA_2405 |
| GO:0009163 | nucleoside biosynthetic process | biological_process | 4 | 3 | 1 | 2.74 | 0.29324 | 0.568288125 | AHA_0238,AHA_1413,AHA_4261,AHA_4267 |
| GO:0030203 | glycosaminoglycan metabolic process | biological_process | 4 | 2 | 2 | 2.74 | 0.29324 | 0.568288125 | AHA_1015,AHA_2130,AHA_2536,AHA_3935 |
| GO:0042455 | ribonucleoside biosynthetic process | biological_process | 4 | 3 | 1 | 2.74 | 0.29324 | 0.568288125 | AHA_0238,AHA_1413,AHA_4261,AHA_4267 |
| GO:0072522 | purine-containing compound biosynthetic process | biological_process | 4 | 3 | 1 | 2.74 | 0.29324 | 0.568288125 | AHA_0238,AHA_1413,AHA_4261,AHA_4267 |
| GO:1901659 | glycosyl compound biosynthetic process | biological_process | 4 | 3 | 1 | 2.74 | 0.29324 | 0.568288125 | AHA_0238,AHA_1413,AHA_4261,AHA_4267 |
| GO:0072521 | purine-containing compound metabolic process | biological_process | 10 | 8 | 2 | 8.13 | 0.29331 | 0.568288125 | AHA_0238,AHA_0278,AHA_0608,AHA_1413,AHA_3545,AHA_3987,AHA_4115,AHA_4261,AHA_4267,AHA_4285 |
| GO:0044712 | single-organism catabolic process | biological_process | 16 | 13 | 3 | 13.76 | 0.29691 | 0.571618698 | AHA_0278,AHA_0290,AHA_0377,AHA_0378,AHA_0379,AHA_0380,AHA_0608,AHA_1008,AHA_1248,AHA_3545,AHA_3987,AHA_4006,AHA_4008,AHA_4115,AHA_4145,AHA_4285 |
| GO:0005740 | mitochondrial envelope | cellular_component | 1 | 0 | 1 | 0.35 | 0.2989 | 0.571618698 | AHA_1921 |
| GO:0015934 | large ribosomal subunit | cellular_component | 1 | 1 | 0 | 0.35 | 0.2989 | 0.571618698 | AHA_4031 |
| GO:0015935 | small ribosomal subunit | cellular_component | 1 | 1 | 0 | 0.35 | 0.2989 | 0.571618698 | AHA_0315 |
| GO:0031966 | mitochondrial membrane | cellular_component | 1 | 0 | 1 | 0.35 | 0.2989 | 0.571618698 | AHA_1921 |
| GO:0009116 | nucleoside metabolic process | biological_process | 10 | 8 | 2 | 8.18 | 0.2997 | 0.571618698 | AHA_0238,AHA_0278,AHA_0608,AHA_1413,AHA_3545,AHA_3987,AHA_4115,AHA_4261,AHA_4267,AHA_4285 |
| GO:1901657 | glycosyl compound metabolic process | biological_process | 10 | 8 | 2 | 8.18 | 0.2997 | 0.571618698 | AHA_0238,AHA_0278,AHA_0608,AHA_1413,AHA_3545,AHA_3987,AHA_4115,AHA_4261,AHA_4267,AHA_4285 |
| GO:0051604 | protein maturation | biological_process | 7 | 6 | 1 | 5.44 | 0.29991 | 0.571618698 | AHA_0612,AHA_0617,AHA_1172,AHA_2163,AHA_2713,AHA_4071,AHA_4114 |
| GO:1901292 | nucleoside phosphate catabolic process | biological_process | 7 | 6 | 1 | 5.44 | 0.29991 | 0.571618698 | AHA_0278,AHA_0608,AHA_1008,AHA_3545,AHA_3987,AHA_4115,AHA_4285 |
| GO:0016860 | intramolecular oxidoreductase activity | molecular_function | 2 | 1 | 1 | 1.1 | 0.30111 | 0.572117266 | AHA_2338,AHA_2926 |
| GO:0016054 | organic acid catabolic process | biological_process | 5 | 5 | 0 | 3.66 | 0.3018 | 0.572117266 | AHA_0290,AHA_0377,AHA_0378,AHA_0379,AHA_0380 |
| GO:0046395 | carboxylic acid catabolic process | biological_process | 5 | 5 | 0 | 3.66 | 0.3018 | 0.572117266 | AHA_0290,AHA_0377,AHA_0378,AHA_0379,AHA_0380 |
| GO:0005215 | transporter activity | molecular_function | 27 | 19 | 8 | 24.35 | 0.30487 | 0.576899425 | AHA_0021,AHA_0238,AHA_0286,AHA_0608,AHA_0676,AHA_0847,AHA_1419,AHA_1595,AHA_1687,AHA_1713,AHA_1738,AHA_1866,AHA_2296,AHA_2350,AHA_2743,AHA_2806,AHA_2812,AHA_2813,AHA_3004,AHA_3053,AHA_3324,AHA_3604,AHA_3769,AHA_3987,AHA_4006,AHA_4261,AHA_4267 |
| GO:0016835 | carbon-oxygen lyase activity | molecular_function | 4 | 3 | 1 | 2.82 | 0.3101 | 0.585744444 | AHA_0288,AHA_0379,AHA_2060,AHA_2928 |
| GO:0016879 | ligase activity, forming carbon-nitrogen bonds | molecular_function | 3 | 3 | 0 | 1.96 | 0.31121 | 0.586789517 | AHA_0596,AHA_3263,AHA_4068 |
| GO:0004022 | alcohol dehydrogenase (NAD) activity | molecular_function | 1 | 0 | 1 | 0.38 | 0.32416 | 0.605715986 | AHA_1331 |
| GO:0008253 | 5'-nucleotidase activity | molecular_function | 1 | 1 | 0 | 0.38 | 0.32416 | 0.605715986 | AHA_1008 |
| GO:0016725 | oxidoreductase activity, acting on CH or CH2 groups | molecular_function | 1 | 1 | 0 | 0.38 | 0.32416 | 0.605715986 | AHA_2333 |
| GO:0019877 | diaminopimelate biosynthetic process | biological_process | 1 | 1 | 0 | 0.39 | 0.32642 | 0.605715986 | AHA_1170 |
| GO:0022618 | ribonucleoprotein complex assembly | biological_process | 1 | 1 | 0 | 0.39 | 0.32642 | 0.605715986 | AHA_2715 |
| GO:0042255 | ribosome assembly | biological_process | 1 | 1 | 0 | 0.39 | 0.32642 | 0.605715986 | AHA_2715 |
| GO:0051259 | protein oligomerization | biological_process | 1 | 1 | 0 | 0.39 | 0.32642 | 0.605715986 | AHA_0295 |
| GO:0070925 | organelle assembly | biological_process | 1 | 1 | 0 | 0.39 | 0.32642 | 0.605715986 | AHA_2715 |
| GO:0071826 | ribonucleoprotein complex subunit organization | biological_process | 1 | 1 | 0 | 0.39 | 0.32642 | 0.605715986 | AHA_2715 |
| GO:0016810 | hydrolase activity, acting on carbon-nitrogen (but not peptide) bonds | molecular_function | 4 | 3 | 1 | 2.91 | 0.33216 | 0.614037631 | AHA_0377,AHA_0378,AHA_0481,AHA_4258 |
| GO:0019866 | organelle inner membrane | cellular_component | 1 | 0 | 1 | 0.4 | 0.3336 | 0.614037631 | AHA_1921 |
| GO:0098588 | bounding membrane of organelle | cellular_component | 1 | 0 | 1 | 0.4 | 0.3336 | 0.614037631 | AHA_2046 |
| GO:0098589 | membrane region | cellular_component | 1 | 0 | 1 | 0.4 | 0.3336 | 0.614037631 | AHA_2046 |
| GO:1990204 | oxidoreductase complex | cellular_component | 2 | 1 | 1 | 1.19 | 0.3341 | 0.614037631 | AHA_1652,AHA_2333 |
| GO:0042597 | periplasmic space | cellular_component | 9 | 8 | 1 | 7.51 | 0.3344 | 0.614037631 | AHA_0292,AHA_1595,AHA_1866,AHA_2452,AHA_2835,AHA_3324,AHA_3479,AHA_3728,AHA_3769 |
| GO:0046914 | transition metal ion binding | molecular_function | 10 | 6 | 4 | 8.45 | 0.33603 | 0.6159576 | AHA_1110,AHA_1834,AHA_1948,AHA_1956,AHA_2405,AHA_2744,AHA_3125,AHA_3601,AHA_3874,AHA_3875 |
| GO:0009260 | ribonucleotide biosynthetic process | biological_process | 4 | 3 | 1 | 2.94 | 0.33767 | 0.617889201 | AHA_0238,AHA_1413,AHA_4261,AHA_4267 |
| GO:0044765 | single-organism transport | biological_process | 20 | 17 | 3 | 18.1 | 0.34846 | 0.629323648 | AHA_0022,AHA_0608,AHA_0676,AHA_0847,AHA_0913,AHA_1419,AHA_1713,AHA_1738,AHA_1964,AHA_2744,AHA_2812,AHA_2813,AHA_3053,AHA_3604,AHA_3728,AHA_3877,AHA_4006,AHA_4261,AHA_4267,AHA_4285 |
| GO:0043234 | protein complex | cellular_component | 8 | 6 | 2 | 6.67 | 0.3487 | 0.629323648 | AHA_1652,AHA_2333,AHA_3324,AHA_3987,AHA_4114,AHA_4115,AHA_4261,AHA_4267 |
| GO:0009203 | ribonucleoside triphosphate catabolic process | biological_process | 6 | 5 | 1 | 4.81 | 0.34953 | 0.629323648 | AHA_0278,AHA_0608,AHA_3545,AHA_3987,AHA_4115,AHA_4285 |
| GO:0009207 | purine ribonucleoside triphosphate catabolic process | biological_process | 6 | 5 | 1 | 4.81 | 0.34953 | 0.629323648 | AHA_0278,AHA_0608,AHA_3545,AHA_3987,AHA_4115,AHA_4285 |
| GO:0015144 | carbohydrate transmembrane transporter activity | molecular_function | 3 | 1 | 2 | 2.1 | 0.3512 | 0.629323648 | AHA_0286,AHA_1419,AHA_4006 |
| GO:1901476 | carbohydrate transporter activity | molecular_function | 3 | 1 | 2 | 2.1 | 0.3512 | 0.629323648 | AHA_0286,AHA_1419,AHA_4006 |
| GO:0044461 | bacterial-type flagellum part | cellular_component | 2 | 2 | 0 | 1.23 | 0.3524 | 0.629323648 | AHA_2835,AHA_2837 |
| GO:0005351 | sugar:hydrogen symporter activity | molecular_function | 2 | 0 | 2 | 1.24 | 0.35459 | 0.629323648 | AHA_1419,AHA_4006 |
| GO:0016763 | transferase activity, transferring pentosyl groups | molecular_function | 2 | 1 | 1 | 1.24 | 0.35459 | 0.629323648 | AHA_0988,AHA_2130 |
| GO:0003916 | DNA topoisomerase activity | molecular_function | 1 | 0 | 1 | 0.43 | 0.35651 | 0.629323648 | AHA_0262 |
| GO:0008144 | drug binding | molecular_function | 1 | 1 | 0 | 0.43 | 0.35651 | 0.629323648 | AHA_4258 |
| GO:0008289 | lipid binding | molecular_function | 1 | 1 | 0 | 0.43 | 0.35651 | 0.629323648 | AHA_4267 |
| GO:0008658 | penicillin binding | molecular_function | 1 | 1 | 0 | 0.43 | 0.35651 | 0.629323648 | AHA_4258 |
| GO:0015489 | putrescine transmembrane transporter activity | molecular_function | 1 | 1 | 0 | 0.43 | 0.35651 | 0.629323648 | AHA_3004 |
| GO:0015662 | ATPase activity, coupled to transmembrane movement of ions, phosphorylative mechanism | molecular_function | 1 | 0 | 1 | 0.43 | 0.35651 | 0.629323648 | AHA_0238 |
| GO:0016646 | oxidoreductase activity, acting on the CH-NH group of donors, NAD or NADP as acceptor | molecular_function | 1 | 0 | 1 | 0.43 | 0.35651 | 0.629323648 | AHA_3949 |
| GO:0009146 | purine nucleoside triphosphate catabolic process | biological_process | 6 | 5 | 1 | 4.86 | 0.35833 | 0.629323648 | AHA_0278,AHA_0608,AHA_3545,AHA_3987,AHA_4115,AHA_4285 |
| GO:0009154 | purine ribonucleotide catabolic process | biological_process | 6 | 5 | 1 | 4.86 | 0.35833 | 0.629323648 | AHA_0278,AHA_0608,AHA_3545,AHA_3987,AHA_4115,AHA_4285 |
| GO:0006265 | DNA topological change | biological_process | 1 | 0 | 1 | 0.43 | 0.35893 | 0.629323648 | AHA_0262 |
| GO:0015893 | drug transport | biological_process | 1 | 1 | 0 | 0.43 | 0.35893 | 0.629323648 | AHA_1713 |
| GO:0044403 | symbiosis, encompassing mutualism through parasitism | biological_process | 1 | 1 | 0 | 0.43 | 0.35893 | 0.629323648 | AHA_0452 |
| GO:0044419 | interspecies interaction between organisms | biological_process | 1 | 1 | 0 | 0.43 | 0.35893 | 0.629323648 | AHA_0452 |
| GO:0046916 | cellular transition metal ion homeostasis | biological_process | 1 | 1 | 0 | 0.43 | 0.35893 | 0.629323648 | AHA_1110 |
| GO:0042623 | ATPase activity, coupled | molecular_function | 6 | 4 | 2 | 4.87 | 0.35987 | 0.629323648 | AHA_0238,AHA_0608,AHA_1595,AHA_2715,AHA_3987,AHA_4261 |
| GO:0006022 | aminoglycan metabolic process | biological_process | 4 | 2 | 2 | 3.03 | 0.36004 | 0.629323648 | AHA_1015,AHA_2130,AHA_2536,AHA_3935 |
| GO:0046390 | ribose phosphate biosynthetic process | biological_process | 4 | 3 | 1 | 3.03 | 0.36004 | 0.629323648 | AHA_0238,AHA_1413,AHA_4261,AHA_4267 |
| GO:0048869 | cellular developmental process | biological_process | 4 | 2 | 2 | 3.03 | 0.36004 | 0.629323648 | AHA_1015,AHA_2130,AHA_2536,AHA_3935 |
| GO:0016887 | ATPase activity | molecular_function | 9 | 7 | 2 | 7.69 | 0.36098 | 0.629922053 | AHA_0238,AHA_0608,AHA_1595,AHA_2715,AHA_3545,AHA_3987,AHA_4115,AHA_4261,AHA_4285 |
| GO:0015293 | symporter activity | molecular_function | 4 | 2 | 2 | 3.06 | 0.36538 | 0.63436977 | AHA_0286,AHA_1419,AHA_3004,AHA_4006 |
| GO:0032155 | cell division site part | cellular_component | 1 | 1 | 0 | 0.44 | 0.3667 | 0.63436977 | AHA_0292 |
| GO:0006152 | purine nucleoside catabolic process | biological_process | 6 | 5 | 1 | 4.91 | 0.36714 | 0.63436977 | AHA_0278,AHA_0608,AHA_3545,AHA_3987,AHA_4115,AHA_4285 |
| GO:0009143 | nucleoside triphosphate catabolic process | biological_process | 6 | 5 | 1 | 4.91 | 0.36714 | 0.63436977 | AHA_0278,AHA_0608,AHA_3545,AHA_3987,AHA_4115,AHA_4285 |
| GO:0009261 | ribonucleotide catabolic process | biological_process | 6 | 5 | 1 | 4.91 | 0.36714 | 0.63436977 | AHA_0278,AHA_0608,AHA_3545,AHA_3987,AHA_4115,AHA_4285 |
| GO:0046130 | purine ribonucleoside catabolic process | biological_process | 6 | 5 | 1 | 4.91 | 0.36714 | 0.63436977 | AHA_0278,AHA_0608,AHA_3545,AHA_3987,AHA_4115,AHA_4285 |
| GO:0046434 | organophosphate catabolic process | biological_process | 7 | 6 | 1 | 5.87 | 0.37182 | 0.641404714 | AHA_0278,AHA_0608,AHA_1008,AHA_3545,AHA_3987,AHA_4115,AHA_4285 |
| GO:0006195 | purine nucleotide catabolic process | biological_process | 6 | 5 | 1 | 4.96 | 0.37596 | 0.646430408 | AHA_0278,AHA_0608,AHA_3545,AHA_3987,AHA_4115,AHA_4285 |
| GO:0042454 | ribonucleoside catabolic process | biological_process | 6 | 5 | 1 | 4.96 | 0.37596 | 0.646430408 | AHA_0278,AHA_0608,AHA_3545,AHA_3987,AHA_4115,AHA_4285 |
| GO:0044724 | single-organism carbohydrate catabolic process | biological_process | 4 | 2 | 2 | 3.13 | 0.38239 | 0.654369522 | AHA_0290,AHA_4006,AHA_4008,AHA_4145 |
| GO:0003724 | RNA helicase activity | molecular_function | 1 | 1 | 0 | 0.48 | 0.38733 | 0.654369522 | AHA_2715 |
| GO:0004004 | ATP-dependent RNA helicase activity | molecular_function | 1 | 1 | 0 | 0.48 | 0.38733 | 0.654369522 | AHA_2715 |
| GO:0015450 | P-P-bond-hydrolysis-driven protein transmembrane transporter activity | molecular_function | 1 | 1 | 0 | 0.48 | 0.38733 | 0.654369522 | AHA_1738 |
| GO:0044463 | cell projection part | cellular_component | 2 | 2 | 0 | 1.33 | 0.3886 | 0.654369522 | AHA_2835,AHA_2837 |
| GO:0006200 | ATP catabolic process | biological_process | 5 | 4 | 1 | 4.09 | 0.38945 | 0.654369522 | AHA_0608,AHA_3545,AHA_3987,AHA_4115,AHA_4285 |
| GO:0009128 | purine nucleoside monophosphate catabolic process | biological_process | 5 | 4 | 1 | 4.09 | 0.38945 | 0.654369522 | AHA_0608,AHA_3545,AHA_3987,AHA_4115,AHA_4285 |
| GO:0009169 | purine ribonucleoside monophosphate catabolic process | biological_process | 5 | 4 | 1 | 4.09 | 0.38945 | 0.654369522 | AHA_0608,AHA_3545,AHA_3987,AHA_4115,AHA_4285 |
| GO:0006549 | isoleucine metabolic process | biological_process | 1 | 1 | 0 | 0.48 | 0.38989 | 0.654369522 | AHA_4201 |
| GO:0006566 | threonine metabolic process | biological_process | 1 | 1 | 0 | 0.48 | 0.38989 | 0.654369522 | AHA_4201 |
| GO:0006721 | terpenoid metabolic process | biological_process | 1 | 1 | 0 | 0.48 | 0.38989 | 0.654369522 | AHA_3601 |
| GO:0009097 | isoleucine biosynthetic process | biological_process | 1 | 1 | 0 | 0.48 | 0.38989 | 0.654369522 | AHA_4201 |
| GO:0009423 | chorismate biosynthetic process | biological_process | 1 | 0 | 1 | 0.48 | 0.38989 | 0.654369522 | AHA_1654 |
| GO:0016114 | terpenoid biosynthetic process | biological_process | 1 | 1 | 0 | 0.48 | 0.38989 | 0.654369522 | AHA_3601 |
| GO:0019954 | asexual reproduction | biological_process | 1 | 0 | 1 | 0.48 | 0.38989 | 0.654369522 | AHA_2405 |
| GO:0009164 | nucleoside catabolic process | biological_process | 6 | 5 | 1 | 5.05 | 0.39362 | 0.658532508 | AHA_0278,AHA_0608,AHA_3545,AHA_3987,AHA_4115,AHA_4285 |
| GO:1901658 | glycosyl compound catabolic process | biological_process | 6 | 5 | 1 | 5.05 | 0.39362 | 0.658532508 | AHA_0278,AHA_0608,AHA_3545,AHA_3987,AHA_4115,AHA_4285 |
| GO:0034655 | nucleobase-containing compound catabolic process | biological_process | 7 | 6 | 1 | 6.02 | 0.39615 | 0.661714897 | AHA_0278,AHA_0608,AHA_1008,AHA_3545,AHA_3987,AHA_4115,AHA_4285 |
| GO:0032153 | cell division site | cellular_component | 1 | 1 | 0 | 0.49 | 0.3981 | 0.663505449 | AHA_0292 |
| GO:0009125 | nucleoside monophosphate catabolic process | biological_process | 5 | 4 | 1 | 4.14 | 0.39923 | 0.663505449 | AHA_0608,AHA_3545,AHA_3987,AHA_4115,AHA_4285 |
| GO:0009158 | ribonucleoside monophosphate catabolic process | biological_process | 5 | 4 | 1 | 4.14 | 0.39923 | 0.663505449 | AHA_0608,AHA_3545,AHA_3987,AHA_4115,AHA_4285 |
| GO:0071704 | organic substance metabolic process | biological_process | 89 | 56 | 33 | 87.16 | 0.39974 | 0.663505449 | AHA_0238,AHA_0262,AHA_0278,AHA_0286,AHA_0288,AHA_0290,AHA_0315,AHA_0377,AHA_0378,AHA_0379,AHA_0380,AHA_0481,AHA_0596,AHA_0608,AHA_0612,AHA_0617,AHA_0677,AHA_0724,AHA_0803,AHA_0859,AHA_0860,AHA_0988,AHA_1008,AHA_1015,AHA_1040,AHA_1153,AHA_1170,AHA_1172,AHA_1201,AHA_1213,AHA_1248,AHA_1270,AHA_1338,AHA_1339,AHA_1413,AHA_1522,AHA_1652,AHA_1654,AHA_1805,AHA_1834,AHA_1956,AHA_1960,AHA_2060,AHA_2130,AHA_2163,AHA_2305,AHA_2333,AHA_2338,AHA_2405,AHA_2490,AHA_2536,AHA_2538,AHA_2561,AHA_2713,AHA_2807,AHA_2851,AHA_2923,AHA_2926,AHA_2982,AHA_2983,AHA_2984,AHA_3000,AHA_3004,AHA_3415,AHA_3545,AHA_3561,AHA_3601,AHA_3606,AHA_3740,AHA_3821,AHA_3874,AHA_3875,AHA_3935,AHA_3949,AHA_3987,AHA_4006,AHA_4008,AHA_4031,AHA_4067,AHA_4068,AHA_4070,AHA_4071,AHA_4114,AHA_4115,AHA_4145,AHA_4201,AHA_4261,AHA_4267,AHA_4285 |
| GO:0006508 | proteolysis | biological_process | 6 | 5 | 1 | 5.15 | 0.41127 | 0.680900341 | AHA_0612,AHA_0617,AHA_1172,AHA_2163,AHA_2713,AHA_4114 |
| GO:0044723 | single-organism carbohydrate metabolic process | biological_process | 10 | 5 | 5 | 9 | 0.41226 | 0.680900341 | AHA_0286,AHA_0288,AHA_0290,AHA_0988,AHA_1213,AHA_1652,AHA_2338,AHA_4006,AHA_4008,AHA_4145 |
| GO:0016829 | lyase activity | molecular_function | 11 | 10 | 1 | 9.98 | 0.41356 | 0.680900341 | AHA_0288,AHA_0289,AHA_0379,AHA_0380,AHA_1270,AHA_1413,AHA_2060,AHA_2923,AHA_2926,AHA_2928,AHA_4201 |
| GO:0008186 | RNA-dependent ATPase activity | molecular_function | 1 | 1 | 0 | 0.53 | 0.41668 | 0.680900341 | AHA_2715 |
| GO:0008252 | nucleotidase activity | molecular_function | 1 | 1 | 0 | 0.53 | 0.41668 | 0.680900341 | AHA_1008 |
| GO:0015101 | organic cation transmembrane transporter activity | molecular_function | 1 | 1 | 0 | 0.53 | 0.41668 | 0.680900341 | AHA_3004 |
| GO:0015203 | polyamine transmembrane transporter activity | molecular_function | 1 | 1 | 0 | 0.53 | 0.41668 | 0.680900341 | AHA_3004 |
| GO:0016645 | oxidoreductase activity, acting on the CH-NH group of donors | molecular_function | 1 | 0 | 1 | 0.53 | 0.41668 | 0.680900341 | AHA_3949 |
| GO:0033293 | monocarboxylic acid binding | molecular_function | 1 | 1 | 0 | 0.53 | 0.41668 | 0.680900341 | AHA_4258 |
| GO:0071111 | cyclic-guanylate-specific phosphodiesterase activity | molecular_function | 1 | 1 | 0 | 0.53 | 0.41668 | 0.680900341 | AHA_0382 |
| GO:0006875 | cellular metal ion homeostasis | biological_process | 1 | 1 | 0 | 0.53 | 0.41937 | 0.683177713 | AHA_1110 |
| GO:0030003 | cellular cation homeostasis | biological_process | 1 | 1 | 0 | 0.53 | 0.41937 | 0.683177713 | AHA_1110 |
| GO:0006575 | cellular modified amino acid metabolic process | biological_process | 2 | 0 | 2 | 1.44 | 0.42773 | 0.695721327 | AHA_2405,AHA_3949 |
| GO:0006260 | DNA replication | biological_process | 3 | 3 | 0 | 2.41 | 0.43549 | 0.706504308 | AHA_2333,AHA_2982,AHA_3875 |
| GO:1902494 | catalytic complex | cellular_component | 4 | 2 | 2 | 3.36 | 0.4357 | 0.706504308 | AHA_1652,AHA_2333,AHA_3324,AHA_3987 |
| GO:0072523 | purine-containing compound catabolic process | biological_process | 6 | 5 | 1 | 5.29 | 0.43768 | 0.708624762 | AHA_0278,AHA_0608,AHA_3545,AHA_3987,AHA_4115,AHA_4285 |
| GO:0009288 | bacterial-type flagellum | cellular_component | 2 | 2 | 0 | 1.48 | 0.4412 | 0.713228221 | AHA_2835,AHA_2837 |
| GO:0005216 | ion channel activity | molecular_function | 1 | 1 | 0 | 0.57 | 0.44463 | 0.713451165 | AHA_3053 |
| GO:0008320 | protein transmembrane transporter activity | molecular_function | 1 | 1 | 0 | 0.57 | 0.44463 | 0.713451165 | AHA_1738 |
| GO:0065007 | biological regulation | biological_process | 24 | 11 | 13 | 23.01 | 0.44464 | 0.713451165 | AHA_0676,AHA_1015,AHA_1110,AHA_1213,AHA_1338,AHA_1339,AHA_1522,AHA_1921,AHA_1960,AHA_1964,AHA_2046,AHA_2130,AHA_2536,AHA_2538,AHA_2561,AHA_2743,AHA_2744,AHA_3000,AHA_3053,AHA_3561,AHA_3874,AHA_3935,AHA_3987,AHA_4031 |
| GO:0006119 | oxidative phosphorylation | biological_process | 1 | 1 | 0 | 0.58 | 0.44743 | 0.713451165 | AHA_1770 |
| GO:0006352 | DNA-templated transcription, initiation | biological_process | 1 | 0 | 1 | 0.58 | 0.44743 | 0.713451165 | AHA_2561 |
| GO:0006730 | one-carbon metabolic process | biological_process | 1 | 0 | 1 | 0.58 | 0.44743 | 0.713451165 | AHA_3949 |
| GO:0006873 | cellular ion homeostasis | biological_process | 1 | 1 | 0 | 0.58 | 0.44743 | 0.713451165 | AHA_1110 |
| GO:0018208 | peptidyl-proline modification | biological_process | 1 | 0 | 1 | 0.58 | 0.44743 | 0.713451165 | AHA_2405 |
| GO:0030163 | protein catabolic process | biological_process | 1 | 1 | 0 | 0.58 | 0.44743 | 0.713451165 | AHA_4114 |
| GO:0044429 | mitochondrial part | cellular_component | 1 | 0 | 1 | 0.59 | 0.4564 | 0.723614586 | AHA_1921 |
| GO:0004812 | aminoacyl-tRNA ligase activity | molecular_function | 2 | 1 | 1 | 1.53 | 0.45655 | 0.723614586 | AHA_2537,AHA_4068 |
| GO:0016875 | ligase activity, forming carbon-oxygen bonds | molecular_function | 2 | 1 | 1 | 1.53 | 0.45655 | 0.723614586 | AHA_2537,AHA_4068 |
| GO:0016876 | ligase activity, forming aminoacyl-tRNA and related compounds | molecular_function | 2 | 1 | 1 | 1.53 | 0.45655 | 0.723614586 | AHA_2537,AHA_4068 |
| GO:0009987 | cellular process | biological_process | 102 | 70 | 32 | 100.97 | 0.46024 | 0.728366147 | AHA_0022,AHA_0238,AHA_0262,AHA_0278,AHA_0286,AHA_0288,AHA_0290,AHA_0292,AHA_0315,AHA_0377,AHA_0378,AHA_0379,AHA_0380,AHA_0382,AHA_0481,AHA_0596,AHA_0608,AHA_0677,AHA_0724,AHA_0735,AHA_0803,AHA_0847,AHA_0859,AHA_0860,AHA_0988,AHA_1008,AHA_1015,AHA_1040,AHA_1110,AHA_1153,AHA_1170,AHA_1172,AHA_1201,AHA_1213,AHA_1270,AHA_1338,AHA_1339,AHA_1413,AHA_1522,AHA_1652,AHA_1654,AHA_1713,AHA_1738,AHA_1770,AHA_1805,AHA_1921,AHA_1956,AHA_1960,AHA_2046,AHA_2060,AHA_2130,AHA_2305,AHA_2333,AHA_2405,AHA_2490,AHA_2536,AHA_2538,AHA_2561,AHA_2637,AHA_2715,AHA_2744,AHA_2807,AHA_2812,AHA_2835,AHA_2837,AHA_2851,AHA_2923,AHA_2926,AHA_2982,AHA_2983,AHA_2984,AHA_3000,AHA_3004,AHA_3053,AHA_3415,AHA_3545,AHA_3561,AHA_3601,AHA_3604,AHA_3606,AHA_3728,AHA_3740,AHA_3821,AHA_3874,AHA_3875,AHA_3935,AHA_3949,AHA_3987,AHA_4006,AHA_4008,AHA_4031,AHA_4067,AHA_4068,AHA_4070,AHA_4114,AHA_4115,AHA_4145,AHA_4201,AHA_4258,AHA_4261,AHA_4267,AHA_4285 |
| GO:0001539 | ciliary or bacterial-type flagellar motility | biological_process | 2 | 2 | 0 | 1.54 | 0.46093 | 0.728366147 | AHA_2835,AHA_2837 |
| GO:0004312 | fatty acid synthase activity | molecular_function | 1 | 1 | 0 | 0.62 | 0.47125 | 0.736014875 | AHA_1040 |
| GO:0009982 | pseudouridine synthase activity | molecular_function | 1 | 1 | 0 | 0.62 | 0.47125 | 0.736014875 | AHA_2851 |
| GO:0022838 | substrate-specific channel activity | molecular_function | 1 | 1 | 0 | 0.62 | 0.47125 | 0.736014875 | AHA_3053 |
| GO:0032991 | macromolecular complex | cellular_component | 11 | 9 | 2 | 10.42 | 0.4734 | 0.736014875 | AHA_0315,AHA_1652,AHA_2333,AHA_2715,AHA_3324,AHA_3987,AHA_4031,AHA_4114,AHA_4115,AHA_4261,AHA_4267 |
| GO:0000003 | reproduction | biological_process | 1 | 0 | 1 | 0.63 | 0.47415 | 0.736014875 | AHA_2405 |
| GO:0001522 | pseudouridine synthesis | biological_process | 1 | 1 | 0 | 0.63 | 0.47415 | 0.736014875 | AHA_2851 |
| GO:0006189 | 'de novo' IMP biosynthetic process | biological_process | 1 | 1 | 0 | 0.63 | 0.47415 | 0.736014875 | AHA_1413 |
| GO:0009089 | lysine biosynthetic process via diaminopimelate | biological_process | 1 | 1 | 0 | 0.63 | 0.47415 | 0.736014875 | AHA_1170 |
| GO:0046417 | chorismate metabolic process | biological_process | 1 | 0 | 1 | 0.63 | 0.47415 | 0.736014875 | AHA_1654 |
| GO:0046451 | diaminopimelate metabolic process | biological_process | 1 | 1 | 0 | 0.63 | 0.47415 | 0.736014875 | AHA_1170 |
| GO:0046474 | glycerophospholipid biosynthetic process | biological_process | 1 | 1 | 0 | 0.63 | 0.47415 | 0.736014875 | AHA_3740 |
| GO:0072348 | sulfur compound transport | biological_process | 1 | 1 | 0 | 0.63 | 0.47415 | 0.736014875 | AHA_0608 |
| GO:0048870 | cell motility | biological_process | 2 | 2 | 0 | 1.59 | 0.47711 | 0.738434567 | AHA_2835,AHA_2837 |
| GO:0051674 | localization of cell | biological_process | 2 | 2 | 0 | 1.59 | 0.47711 | 0.738434567 | AHA_2835,AHA_2837 |
| GO:0044085 | cellular component biogenesis | biological_process | 8 | 6 | 2 | 7.51 | 0.48048 | 0.741421199 | AHA_0295,AHA_1015,AHA_2130,AHA_2536,AHA_2715,AHA_2851,AHA_3935,AHA_4258 |
| GO:0008565 | protein transporter activity | molecular_function | 3 | 2 | 1 | 2.58 | 0.48084 | 0.741421199 | AHA_1738,AHA_1866,AHA_3769 |
| GO:0016052 | carbohydrate catabolic process | biological_process | 4 | 2 | 2 | 3.56 | 0.48115 | 0.741421199 | AHA_0290,AHA_4006,AHA_4008,AHA_4145 |
| GO:0031967 | organelle envelope | cellular_component | 1 | 0 | 1 | 0.64 | 0.4834 | 0.743800876 | AHA_1921 |
| GO:0005198 | structural molecule activity | molecular_function | 4 | 4 | 0 | 3.58 | 0.48514 | 0.745390029 | AHA_0315,AHA_2835,AHA_2837,AHA_4031 |
| GO:0044272 | sulfur compound biosynthetic process | biological_process | 3 | 0 | 3 | 2.6 | 0.48649 | 0.746376215 | AHA_1956,AHA_2807,AHA_3949 |
| GO:0008137 | NADH dehydrogenase (ubiquinone) activity | molecular_function | 1 | 1 | 0 | 0.67 | 0.49661 | 0.756547874 | AHA_1770 |
| GO:0008374 | O-acyltransferase activity | molecular_function | 1 | 1 | 0 | 0.67 | 0.49661 | 0.756547874 | AHA_3740 |
| GO:0030234 | enzyme regulator activity | molecular_function | 1 | 1 | 0 | 0.67 | 0.49661 | 0.756547874 | AHA_2984 |
| GO:0070008 | serine-type exopeptidase activity | molecular_function | 1 | 1 | 0 | 0.67 | 0.49661 | 0.756547874 | AHA_0617 |
| GO:0016462 | pyrophosphatase activity | molecular_function | 13 | 11 | 2 | 12.6 | 0.49735 | 0.756547874 | AHA_0238,AHA_0278,AHA_0608,AHA_1595,AHA_2715,AHA_2837,AHA_3545,AHA_3875,AHA_3987,AHA_4071,AHA_4115,AHA_4261,AHA_4285 |
| GO:0006526 | arginine biosynthetic process | biological_process | 1 | 1 | 0 | 0.67 | 0.49958 | 0.756547874 | AHA_0596 |
| GO:0009085 | lysine biosynthetic process | biological_process | 1 | 1 | 0 | 0.67 | 0.49958 | 0.756547874 | AHA_1170 |
| GO:0044265 | cellular macromolecule catabolic process | biological_process | 1 | 1 | 0 | 0.67 | 0.49958 | 0.756547874 | AHA_4114 |
| GO:0045017 | glycerolipid biosynthetic process | biological_process | 1 | 1 | 0 | 0.67 | 0.49958 | 0.756547874 | AHA_3740 |
| GO:0017111 | nucleoside-triphosphatase activity | molecular_function | 12 | 10 | 2 | 11.65 | 0.50265 | 0.760104878 | AHA_0238,AHA_0278,AHA_0608,AHA_1595,AHA_2715,AHA_2837,AHA_3545,AHA_3987,AHA_4071,AHA_4115,AHA_4261,AHA_4285 |
| GO:0016410 | N-acyltransferase activity | molecular_function | 3 | 1 | 2 | 2.67 | 0.50543 | 0.763213782 | AHA_0724,AHA_1170,AHA_1921 |
| GO:0009401 | phosphoenolpyruvate-dependent sugar phosphotransferase system | biological_process | 2 | 0 | 2 | 1.68 | 0.50858 | 0.765164907 | AHA_1419,AHA_4006 |
| GO:0019725 | cellular homeostasis | biological_process | 2 | 2 | 0 | 1.68 | 0.50858 | 0.765164907 | AHA_1110,AHA_3053 |
| GO:0016818 | hydrolase activity, acting on acid anhydrides, in phosphorus-containing anhydrides | molecular_function | 13 | 11 | 2 | 12.7 | 0.5089 | 0.765164907 | AHA_0238,AHA_0278,AHA_0608,AHA_1595,AHA_2715,AHA_2837,AHA_3545,AHA_3875,AHA_3987,AHA_4071,AHA_4115,AHA_4261,AHA_4285 |
| GO:0046677 | response to antibiotic | biological_process | 3 | 3 | 0 | 2.7 | 0.51116 | 0.767468148 | AHA_0847,AHA_3604,AHA_4258 |
| GO:0016817 | hydrolase activity, acting on acid anhydrides | molecular_function | 13 | 11 | 2 | 12.75 | 0.51466 | 0.771623954 | AHA_0238,AHA_0278,AHA_0608,AHA_1595,AHA_2715,AHA_2837,AHA_3545,AHA_3875,AHA_3987,AHA_4071,AHA_4115,AHA_4261,AHA_4285 |
| GO:0005507 | copper ion binding | molecular_function | 1 | 0 | 1 | 0.72 | 0.52076 | 0.776491139 | AHA_1834 |
| GO:0022884 | macromolecule transmembrane transporter activity | molecular_function | 1 | 1 | 0 | 0.72 | 0.52076 | 0.776491139 | AHA_1738 |
| GO:0006184 | GTP catabolic process | biological_process | 1 | 1 | 0 | 0.72 | 0.5238 | 0.776491139 | AHA_0278 |
| GO:0006188 | IMP biosynthetic process | biological_process | 1 | 1 | 0 | 0.72 | 0.5238 | 0.776491139 | AHA_1413 |
| GO:0006720 | isoprenoid metabolic process | biological_process | 1 | 1 | 0 | 0.72 | 0.5238 | 0.776491139 | AHA_3601 |
| GO:0008299 | isoprenoid biosynthetic process | biological_process | 1 | 1 | 0 | 0.72 | 0.5238 | 0.776491139 | AHA_3601 |
| GO:0046040 | IMP metabolic process | biological_process | 1 | 1 | 0 | 0.72 | 0.5238 | 0.776491139 | AHA_1413 |
| GO:0046653 | tetrahydrofolate metabolic process | biological_process | 1 | 0 | 1 | 0.72 | 0.5238 | 0.776491139 | AHA_3949 |
| GO:0031406 | carboxylic acid binding | molecular_function | 2 | 1 | 1 | 1.77 | 0.53416 | 0.789627826 | AHA_2405,AHA_4258 |
| GO:0043177 | organic acid binding | molecular_function | 2 | 1 | 1 | 1.77 | 0.53416 | 0.789627826 | AHA_2405,AHA_4258 |
| GO:0042995 | cell projection | cellular_component | 2 | 2 | 0 | 1.78 | 0.5385 | 0.794928571 | AHA_2835,AHA_2837 |
| GO:0003700 | sequence-specific DNA binding transcription factor activity | molecular_function | 9 | 3 | 6 | 8.93 | 0.54174 | 0.796894561 | AHA_0290,AHA_0677,AHA_1201,AHA_1213,AHA_1805,AHA_2561,AHA_3606,AHA_3821,AHA_4067 |
| GO:0043228 | non-membrane-bounded organelle | cellular_component | 6 | 5 | 1 | 5.88 | 0.5421 | 0.796894561 | AHA_0262,AHA_0315,AHA_2715,AHA_2835,AHA_2837,AHA_4031 |
| GO:0043232 | intracellular non-membrane-bounded organelle | cellular_component | 6 | 5 | 1 | 5.88 | 0.5421 | 0.796894561 | AHA_0262,AHA_0315,AHA_2715,AHA_2835,AHA_2837,AHA_4031 |
| GO:0015197 | peptide transporter activity | molecular_function | 1 | 1 | 0 | 0.76 | 0.54376 | 0.797111321 | AHA_3324 |
| GO:0016758 | transferase activity, transferring hexosyl groups | molecular_function | 1 | 1 | 0 | 0.76 | 0.54376 | 0.797111321 | AHA_0988 |
| GO:0003723 | RNA binding | molecular_function | 7 | 4 | 3 | 6.92 | 0.5455 | 0.797205947 | AHA_0315,AHA_2423,AHA_2537,AHA_2715,AHA_2851,AHA_3415,AHA_4031 |
| GO:1901137 | carbohydrate derivative biosynthetic process | biological_process | 9 | 6 | 3 | 8.95 | 0.54565 | 0.797205947 | AHA_0238,AHA_0988,AHA_1015,AHA_1413,AHA_2130,AHA_2536,AHA_3935,AHA_4261,AHA_4267 |
| GO:0006760 | folic acid-containing compound metabolic process | biological_process | 1 | 0 | 1 | 0.77 | 0.54685 | 0.797205947 | AHA_3949 |
| GO:0006813 | potassium ion transport | biological_process | 1 | 1 | 0 | 0.77 | 0.54685 | 0.797205947 | AHA_3053 |
| GO:0043650 | dicarboxylic acid biosynthetic process | biological_process | 2 | 1 | 1 | 1.83 | 0.55343 | 0.804572717 | AHA_1170,AHA_1654 |
| GO:0065003 | macromolecular complex assembly | biological_process | 2 | 2 | 0 | 1.83 | 0.55343 | 0.804572717 | AHA_0295,AHA_2715 |
| GO:0001071 | nucleic acid binding transcription factor activity | molecular_function | 9 | 3 | 6 | 9.02 | 0.55508 | 0.805859945 | AHA_0290,AHA_0677,AHA_1201,AHA_1213,AHA_1805,AHA_2561,AHA_3606,AHA_3821,AHA_4067 |
| GO:0040011 | locomotion | biological_process | 5 | 5 | 0 | 4.96 | 0.55947 | 0.811116066 | AHA_1391,AHA_2835,AHA_2837,AHA_3324,AHA_3561 |
| GO:0006807 | nitrogen compound metabolic process | biological_process | 61 | 35 | 26 | 61.36 | 0.56237 | 0.812937205 | AHA_0238,AHA_0262,AHA_0278,AHA_0290,AHA_0377,AHA_0378,AHA_0379,AHA_0380,AHA_0481,AHA_0596,AHA_0608,AHA_0677,AHA_0803,AHA_1008,AHA_1015,AHA_1153,AHA_1170,AHA_1201,AHA_1213,AHA_1270,AHA_1338,AHA_1339,AHA_1413,AHA_1522,AHA_1654,AHA_1805,AHA_1834,AHA_1956,AHA_1960,AHA_2130,AHA_2305,AHA_2333,AHA_2405,AHA_2536,AHA_2538,AHA_2561,AHA_2807,AHA_2851,AHA_2923,AHA_2926,AHA_2982,AHA_3000,AHA_3004,AHA_3415,AHA_3545,AHA_3561,AHA_3606,AHA_3821,AHA_3874,AHA_3875,AHA_3935,AHA_3949,AHA_3987,AHA_4067,AHA_4068,AHA_4070,AHA_4115,AHA_4201,AHA_4261,AHA_4267,AHA_4285 |
| GO:0016791 | phosphatase activity | molecular_function | 2 | 2 | 0 | 1.86 | 0.56304 | 0.812937205 | AHA_1008,AHA_3603 |
| GO:0019843 | rRNA binding | molecular_function | 2 | 2 | 0 | 1.86 | 0.56304 | 0.812937205 | AHA_0315,AHA_4031 |
| GO:0030145 | manganese ion binding | molecular_function | 1 | 1 | 0 | 0.81 | 0.56566 | 0.813439159 | AHA_3875 |
| GO:0006928 | cellular component movement | biological_process | 2 | 2 | 0 | 1.88 | 0.56773 | 0.813439159 | AHA_2835,AHA_2837 |
| GO:0006040 | amino sugar metabolic process | biological_process | 1 | 1 | 0 | 0.82 | 0.56879 | 0.813439159 | AHA_3935 |
| GO:0006553 | lysine metabolic process | biological_process | 1 | 1 | 0 | 0.82 | 0.56879 | 0.813439159 | AHA_1170 |
| GO:0032506 | cytokinetic process | biological_process | 1 | 1 | 0 | 0.82 | 0.56879 | 0.813439159 | AHA_0292 |
| GO:0046039 | GTP metabolic process | biological_process | 1 | 1 | 0 | 0.82 | 0.56879 | 0.813439159 | AHA_0278 |
| GO:1901069 | guanosine-containing compound catabolic process | biological_process | 1 | 1 | 0 | 0.82 | 0.56879 | 0.813439159 | AHA_0278 |
| GO:0003774 | motor activity | molecular_function | 1 | 1 | 0 | 0.86 | 0.58652 | 0.833140375 | AHA_2837 |
| GO:0003924 | GTPase activity | molecular_function | 1 | 1 | 0 | 0.86 | 0.58652 | 0.833140375 | AHA_0278 |
| GO:0003954 | NADH dehydrogenase activity | molecular_function | 1 | 1 | 0 | 0.86 | 0.58652 | 0.833140375 | AHA_1770 |
| GO:0019842 | vitamin binding | molecular_function | 1 | 0 | 1 | 0.86 | 0.58652 | 0.833140375 | AHA_2405 |
| GO:0050136 | NADH dehydrogenase (quinone) activity | molecular_function | 1 | 1 | 0 | 0.86 | 0.58652 | 0.833140375 | AHA_1770 |
| GO:0006541 | glutamine metabolic process | biological_process | 1 | 0 | 1 | 0.87 | 0.58968 | 0.833140375 | AHA_0481 |
| GO:0006814 | sodium ion transport | biological_process | 1 | 0 | 1 | 0.87 | 0.58968 | 0.833140375 | AHA_0676 |
| GO:0009082 | branched-chain amino acid biosynthetic process | biological_process | 1 | 1 | 0 | 0.87 | 0.58968 | 0.833140375 | AHA_4201 |
| GO:0072525 | pyridine-containing compound biosynthetic process | biological_process | 1 | 1 | 0 | 0.87 | 0.58968 | 0.833140375 | AHA_0803 |
| GO:0006790 | sulfur compound metabolic process | biological_process | 3 | 0 | 3 | 3.03 | 0.59233 | 0.83576415 | AHA_1956,AHA_2807,AHA_3949 |
| GO:1901135 | carbohydrate derivative metabolic process | biological_process | 16 | 11 | 5 | 16.51 | 0.59649 | 0.840508636 | AHA_0238,AHA_0278,AHA_0608,AHA_0988,AHA_1015,AHA_1413,AHA_1652,AHA_2130,AHA_2536,AHA_3545,AHA_3935,AHA_3987,AHA_4115,AHA_4261,AHA_4267,AHA_4285 |
| GO:0015077 | monovalent inorganic cation transmembrane transporter activity | molecular_function | 3 | 3 | 0 | 3.06 | 0.5973 | 0.840526302 | AHA_2296,AHA_4261,AHA_4267 |
| GO:0004252 | serine-type endopeptidase activity | molecular_function | 1 | 1 | 0 | 0.91 | 0.60638 | 0.849899628 | AHA_0617 |
| GO:0015238 | drug transmembrane transporter activity | molecular_function | 1 | 1 | 0 | 0.91 | 0.60638 | 0.849899628 | AHA_1713 |
| GO:0090484 | drug transporter activity | molecular_function | 1 | 1 | 0 | 0.91 | 0.60638 | 0.849899628 | AHA_1713 |
| GO:0006417 | regulation of translation | biological_process | 1 | 1 | 0 | 0.91 | 0.60957 | 0.852104483 | AHA_4031 |
| GO:0070887 | cellular response to chemical stimulus | biological_process | 1 | 1 | 0 | 0.91 | 0.60957 | 0.852104483 | AHA_0735 |
| GO:0016747 | transferase activity, transferring acyl groups other than amino-acyl groups | molecular_function | 5 | 3 | 2 | 5.25 | 0.61152 | 0.853698119 | AHA_0724,AHA_1040,AHA_1170,AHA_1921,AHA_3740 |
| GO:0016614 | oxidoreductase activity, acting on CH-OH group of donors | molecular_function | 3 | 1 | 2 | 3.15 | 0.61849 | 0.861147239 | AHA_1331,AHA_1652,AHA_4145 |
| GO:0042578 | phosphoric ester hydrolase activity | molecular_function | 3 | 3 | 0 | 3.15 | 0.61849 | 0.861147239 | AHA_0382,AHA_1008,AHA_3603 |
| GO:1901136 | carbohydrate derivative catabolic process | biological_process | 6 | 5 | 1 | 6.35 | 0.61946 | 0.861359947 | AHA_0278,AHA_0608,AHA_3545,AHA_3987,AHA_4115,AHA_4285 |
| GO:0015031 | protein transport | biological_process | 4 | 4 | 0 | 4.24 | 0.62051 | 0.861683188 | AHA_0295,AHA_1738,AHA_3324,AHA_3877 |
| GO:0043933 | macromolecular complex subunit organization | biological_process | 2 | 2 | 0 | 2.07 | 0.62164 | 0.862116526 | AHA_0295,AHA_2715 |
| GO:0009636 | response to toxic substance | biological_process | 3 | 3 | 0 | 3.18 | 0.62438 | 0.862537057 | AHA_0847,AHA_3604,AHA_4258 |
| GO:0006725 | cellular aromatic compound metabolic process | biological_process | 48 | 29 | 19 | 49.24 | 0.62515 | 0.862537057 | AHA_0238,AHA_0262,AHA_0278,AHA_0290,AHA_0377,AHA_0378,AHA_0379,AHA_0380,AHA_0608,AHA_0677,AHA_0803,AHA_1008,AHA_1153,AHA_1201,AHA_1213,AHA_1338,AHA_1339,AHA_1413,AHA_1522,AHA_1654,AHA_1805,AHA_1960,AHA_2305,AHA_2333,AHA_2538,AHA_2561,AHA_2851,AHA_2923,AHA_2926,AHA_2982,AHA_3000,AHA_3415,AHA_3545,AHA_3561,AHA_3606,AHA_3821,AHA_3874,AHA_3875,AHA_3935,AHA_3949,AHA_3987,AHA_4067,AHA_4068,AHA_4070,AHA_4115,AHA_4261,AHA_4267,AHA_4285 |
| GO:0000049 | tRNA binding | molecular_function | 1 | 1 | 0 | 0.95 | 0.6253 | 0.862537057 | AHA_4031 |
| GO:1901681 | sulfur compound binding | molecular_function | 1 | 1 | 0 | 0.95 | 0.6253 | 0.862537057 | AHA_4258 |
| GO:0006650 | glycerophospholipid metabolic process | biological_process | 1 | 1 | 0 | 0.96 | 0.62849 | 0.862537057 | AHA_3740 |
| GO:0006996 | organelle organization | biological_process | 1 | 1 | 0 | 0.96 | 0.62849 | 0.862537057 | AHA_2715 |
| GO:0009226 | nucleotide-sugar biosynthetic process | biological_process | 1 | 1 | 0 | 0.96 | 0.62849 | 0.862537057 | AHA_3935 |
| GO:0043604 | amide biosynthetic process | biological_process | 1 | 1 | 0 | 0.96 | 0.62849 | 0.862537057 | AHA_4068 |
| GO:0042221 | response to chemical | biological_process | 9 | 9 | 0 | 9.58 | 0.62999 | 0.863471339 | AHA_0292,AHA_0735,AHA_0847,AHA_1391,AHA_1713,AHA_3324,AHA_3561,AHA_3604,AHA_4258 |
| GO:0005840 | ribosome | cellular_component | 3 | 3 | 0 | 3.21 | 0.6325 | 0.865785714 | AHA_0315,AHA_2715,AHA_4031 |
| GO:0005694 | chromosome | cellular_component | 1 | 0 | 1 | 0.99 | 0.6386 | 0.872294223 | AHA_0262 |
| GO:0046873 | metal ion transmembrane transporter activity | molecular_function | 3 | 1 | 2 | 3.25 | 0.63891 | 0.872294223 | AHA_0238,AHA_2743,AHA_3987 |
| GO:0016661 | oxidoreductase activity, acting on other nitrogenous compounds as donors | molecular_function | 1 | 1 | 0 | 1 | 0.64331 | 0.873617359 | AHA_0735 |
| GO:0009225 | nucleotide-sugar metabolic process | biological_process | 1 | 1 | 0 | 1.01 | 0.64651 | 0.873617359 | AHA_3935 |
| GO:0009245 | lipid A biosynthetic process | biological_process | 1 | 1 | 0 | 1.01 | 0.64651 | 0.873617359 | AHA_0988 |
| GO:0010608 | posttranscriptional regulation of gene expression | biological_process | 1 | 1 | 0 | 1.01 | 0.64651 | 0.873617359 | AHA_4031 |
| GO:0046486 | glycerolipid metabolic process | biological_process | 1 | 1 | 0 | 1.01 | 0.64651 | 0.873617359 | AHA_3740 |
| GO:0046493 | lipid A metabolic process | biological_process | 1 | 1 | 0 | 1.01 | 0.64651 | 0.873617359 | AHA_0988 |
| GO:0072329 | monocarboxylic acid catabolic process | biological_process | 1 | 1 | 0 | 1.01 | 0.64651 | 0.873617359 | AHA_0290 |
| GO:1901617 | organic hydroxy compound biosynthetic process | biological_process | 1 | 1 | 0 | 1.01 | 0.64651 | 0.873617359 | AHA_0803 |
| GO:0030554 | adenyl nucleotide binding | molecular_function | 21 | 16 | 5 | 22.29 | 0.65737 | 0.887154904 | AHA_0238,AHA_0596,AHA_0608,AHA_0859,AHA_0860,AHA_2305,AHA_2333,AHA_2490,AHA_2537,AHA_2715,AHA_2982,AHA_2983,AHA_2984,AHA_3545,AHA_3877,AHA_3987,AHA_4006,AHA_4068,AHA_4071,AHA_4115,AHA_4285 |
| GO:0046365 | monosaccharide catabolic process | biological_process | 2 | 1 | 1 | 2.21 | 0.65859 | 0.887664783 | AHA_4008,AHA_4145 |
| GO:0001510 | RNA methylation | biological_process | 1 | 0 | 1 | 1.06 | 0.66366 | 0.891004518 | AHA_3415 |
| GO:0032268 | regulation of cellular protein metabolic process | biological_process | 1 | 1 | 0 | 1.06 | 0.66366 | 0.891004518 | AHA_4031 |
| GO:0045184 | establishment of protein localization | biological_process | 4 | 4 | 0 | 4.48 | 0.66431 | 0.891004518 | AHA_0295,AHA_1738,AHA_3324,AHA_3877 |
| GO:0046483 | heterocycle metabolic process | biological_process | 48 | 29 | 19 | 49.81 | 0.66528 | 0.891004518 | AHA_0238,AHA_0262,AHA_0278,AHA_0290,AHA_0377,AHA_0378,AHA_0379,AHA_0380,AHA_0608,AHA_0677,AHA_0803,AHA_1008,AHA_1153,AHA_1201,AHA_1213,AHA_1338,AHA_1339,AHA_1413,AHA_1522,AHA_1805,AHA_1960,AHA_2305,AHA_2333,AHA_2405,AHA_2538,AHA_2561,AHA_2851,AHA_2923,AHA_2926,AHA_2982,AHA_3000,AHA_3415,AHA_3545,AHA_3561,AHA_3606,AHA_3821,AHA_3874,AHA_3875,AHA_3935,AHA_3949,AHA_3987,AHA_4067,AHA_4068,AHA_4070,AHA_4115,AHA_4261,AHA_4267,AHA_4285 |
| GO:1901360 | organic cyclic compound metabolic process | biological_process | 49 | 29 | 20 | 50.82 | 0.66561 | 0.891004518 | AHA_0238,AHA_0262,AHA_0278,AHA_0290,AHA_0377,AHA_0378,AHA_0379,AHA_0380,AHA_0608,AHA_0677,AHA_0803,AHA_1008,AHA_1153,AHA_1201,AHA_1213,AHA_1338,AHA_1339,AHA_1413,AHA_1522,AHA_1654,AHA_1805,AHA_1960,AHA_2305,AHA_2333,AHA_2405,AHA_2538,AHA_2561,AHA_2851,AHA_2923,AHA_2926,AHA_2982,AHA_3000,AHA_3415,AHA_3545,AHA_3561,AHA_3606,AHA_3821,AHA_3874,AHA_3875,AHA_3935,AHA_3949,AHA_3987,AHA_4067,AHA_4068,AHA_4070,AHA_4115,AHA_4261,AHA_4267,AHA_4285 |
| GO:0016787 | hydrolase activity | molecular_function | 31 | 25 | 6 | 32.65 | 0.66614 | 0.891004518 | AHA_0238,AHA_0278,AHA_0292,AHA_0377,AHA_0378,AHA_0382,AHA_0481,AHA_0608,AHA_0612,AHA_0617,AHA_1008,AHA_1153,AHA_1172,AHA_1595,AHA_2163,AHA_2360,AHA_2713,AHA_2715,AHA_2837,AHA_2940,AHA_3545,AHA_3603,AHA_3875,AHA_3987,AHA_4070,AHA_4071,AHA_4114,AHA_4115,AHA_4258,AHA_4261,AHA_4285 |
| GO:0022613 | ribonucleoprotein complex biogenesis | biological_process | 2 | 2 | 0 | 2.26 | 0.67026 | 0.891231719 | AHA_2715,AHA_2851 |
| GO:0042254 | ribosome biogenesis | biological_process | 2 | 2 | 0 | 2.26 | 0.67026 | 0.891231719 | AHA_2715,AHA_2851 |
| GO:0043226 | organelle | cellular_component | 8 | 5 | 3 | 8.84 | 0.6726 | 0.891231719 | AHA_0262,AHA_0315,AHA_1921,AHA_2046,AHA_2715,AHA_2835,AHA_2837,AHA_4031 |
| GO:0043229 | intracellular organelle | cellular_component | 8 | 5 | 3 | 8.84 | 0.6726 | 0.891231719 | AHA_0262,AHA_0315,AHA_1921,AHA_2046,AHA_2715,AHA_2835,AHA_2837,AHA_4031 |
| GO:0008104 | protein localization | biological_process | 4 | 4 | 0 | 4.52 | 0.67266 | 0.891231719 | AHA_0295,AHA_1738,AHA_3324,AHA_3877 |
| GO:0030529 | ribonucleoprotein complex | cellular_component | 3 | 3 | 0 | 3.41 | 0.6731 | 0.891231719 | AHA_0315,AHA_2715,AHA_4031 |
| GO:0009274 | peptidoglycan-based cell wall | cellular_component | 1 | 0 | 1 | 1.09 | 0.6737 | 0.891231719 | AHA_2130 |
| GO:0006464 | cellular protein modification process | biological_process | 3 | 1 | 2 | 3.42 | 0.67392 | 0.891231719 | AHA_0724,AHA_0988,AHA_2405 |
| GO:0036211 | protein modification process | biological_process | 3 | 1 | 2 | 3.42 | 0.67392 | 0.891231719 | AHA_0724,AHA_0988,AHA_2405 |
| GO:0003677 | DNA binding | molecular_function | 16 | 4 | 12 | 17.33 | 0.67664 | 0.891429677 | AHA_0290,AHA_0677,AHA_1153,AHA_1201,AHA_1213,AHA_1338,AHA_1339,AHA_1522,AHA_1805,AHA_1960,AHA_2538,AHA_2561,AHA_3000,AHA_3606,AHA_3821,AHA_4067 |
| GO:0008982 | protein-N(PI)-phosphohistidine-sugar phosphotransferase activity | molecular_function | 1 | 0 | 1 | 1.1 | 0.6768 | 0.891429677 | AHA_1419 |
| GO:0010181 | FMN binding | molecular_function | 1 | 1 | 0 | 1.1 | 0.6768 | 0.891429677 | AHA_0735 |
| GO:0071840 | cellular component organization or biogenesis | biological_process | 8 | 6 | 2 | 8.9 | 0.67868 | 0.891429677 | AHA_0295,AHA_1015,AHA_2130,AHA_2536,AHA_2715,AHA_2851,AHA_3935,AHA_4258 |
| GO:0022904 | respiratory electron transport chain | biological_process | 1 | 1 | 0 | 1.11 | 0.67999 | 0.891429677 | AHA_1770 |
| GO:1901269 | lipooligosaccharide metabolic process | biological_process | 1 | 1 | 0 | 1.11 | 0.67999 | 0.891429677 | AHA_0988 |
| GO:1901271 | lipooligosaccharide biosynthetic process | biological_process | 1 | 1 | 0 | 1.11 | 0.67999 | 0.891429677 | AHA_0988 |
| GO:0016043 | cellular component organization | biological_process | 6 | 4 | 2 | 6.79 | 0.68378 | 0.895284621 | AHA_0295,AHA_1015,AHA_2130,AHA_2536,AHA_2715,AHA_3935 |
| GO:0005739 | mitochondrion | cellular_component | 1 | 0 | 1 | 1.14 | 0.69 | 0.900162787 | AHA_1921 |
| GO:0008026 | ATP-dependent helicase activity | molecular_function | 1 | 1 | 0 | 1.15 | 0.69235 | 0.900162787 | AHA_2715 |
| GO:0070035 | purine NTP-dependent helicase activity | molecular_function | 1 | 1 | 0 | 1.15 | 0.69235 | 0.900162787 | AHA_2715 |
| GO:0005996 | monosaccharide metabolic process | biological_process | 3 | 1 | 2 | 3.51 | 0.69236 | 0.900162787 | AHA_2338,AHA_4008,AHA_4145 |
| GO:0006351 | transcription, DNA-templated | biological_process | 17 | 6 | 11 | 18.53 | 0.6924 | 0.900162787 | AHA_0290,AHA_0677,AHA_1201,AHA_1213,AHA_1338,AHA_1339,AHA_1522,AHA_1805,AHA_1960,AHA_2538,AHA_2561,AHA_3000,AHA_3561,AHA_3606,AHA_3821,AHA_3874,AHA_4067 |
| GO:0051649 | establishment of localization in cell | biological_process | 2 | 2 | 0 | 2.36 | 0.69263 | 0.900162787 | AHA_1738,AHA_3877 |
| GO:1901068 | guanosine-containing compound metabolic process | biological_process | 1 | 1 | 0 | 1.16 | 0.69552 | 0.902805517 | AHA_0278 |
| GO:0005975 | carbohydrate metabolic process | biological_process | 10 | 5 | 5 | 11.21 | 0.69873 | 0.905856605 | AHA_0286,AHA_0288,AHA_0290,AHA_0988,AHA_1213,AHA_1652,AHA_2338,AHA_4006,AHA_4008,AHA_4145 |
| GO:0008654 | phospholipid biosynthetic process | biological_process | 2 | 2 | 0 | 2.41 | 0.70335 | 0.907832558 | AHA_0988,AHA_3740 |
| GO:0034641 | cellular nitrogen compound metabolic process | biological_process | 48 | 30 | 18 | 50.39 | 0.7035 | 0.907832558 | AHA_0238,AHA_0262,AHA_0278,AHA_0290,AHA_0377,AHA_0378,AHA_0379,AHA_0380,AHA_0481,AHA_0608,AHA_0677,AHA_0803,AHA_1008,AHA_1153,AHA_1201,AHA_1213,AHA_1270,AHA_1338,AHA_1339,AHA_1413,AHA_1522,AHA_1805,AHA_1960,AHA_2305,AHA_2333,AHA_2538,AHA_2561,AHA_2851,AHA_2923,AHA_2926,AHA_2982,AHA_3000,AHA_3004,AHA_3415,AHA_3545,AHA_3561,AHA_3606,AHA_3821,AHA_3874,AHA_3875,AHA_3935,AHA_3987,AHA_4067,AHA_4068,AHA_4115,AHA_4261,AHA_4267,AHA_4285 |
| GO:0008270 | zinc ion binding | molecular_function | 3 | 1 | 2 | 3.58 | 0.70434 | 0.907832558 | AHA_1956,AHA_3125,AHA_3874 |
| GO:0044260 | cellular macromolecule metabolic process | biological_process | 41 | 22 | 19 | 43.36 | 0.70671 | 0.907832558 | AHA_0262,AHA_0290,AHA_0315,AHA_0677,AHA_0724,AHA_0859,AHA_0860,AHA_0988,AHA_1015,AHA_1153,AHA_1201,AHA_1213,AHA_1338,AHA_1339,AHA_1522,AHA_1805,AHA_1960,AHA_2130,AHA_2305,AHA_2333,AHA_2405,AHA_2490,AHA_2536,AHA_2538,AHA_2561,AHA_2851,AHA_2982,AHA_2983,AHA_2984,AHA_3000,AHA_3415,AHA_3561,AHA_3606,AHA_3821,AHA_3874,AHA_3875,AHA_3935,AHA_4031,AHA_4067,AHA_4068,AHA_4114 |
| GO:0016811 | hydrolase activity, acting on carbon-nitrogen (but not peptide) bonds, in linear amides | molecular_function | 1 | 0 | 1 | 1.19 | 0.70716 | 0.907832558 | AHA_0481 |
| GO:0030246 | carbohydrate binding | molecular_function | 1 | 0 | 1 | 1.19 | 0.70716 | 0.907832558 | AHA_2405 |
| GO:0006525 | arginine metabolic process | biological_process | 1 | 1 | 0 | 1.2 | 0.71031 | 0.907832558 | AHA_0596 |
| GO:0009084 | glutamine family amino acid biosynthetic process | biological_process | 1 | 1 | 0 | 1.2 | 0.71031 | 0.907832558 | AHA_0596 |
| GO:0018193 | peptidyl-amino acid modification | biological_process | 1 | 0 | 1 | 1.2 | 0.71031 | 0.907832558 | AHA_2405 |
| GO:0043414 | macromolecule methylation | biological_process | 1 | 0 | 1 | 1.2 | 0.71031 | 0.907832558 | AHA_3415 |
| GO:0051246 | regulation of protein metabolic process | biological_process | 1 | 1 | 0 | 1.2 | 0.71031 | 0.907832558 | AHA_4031 |
| GO:0017076 | purine nucleotide binding | molecular_function | 23 | 18 | 5 | 25.01 | 0.71059 | 0.907832558 | AHA_0238,AHA_0278,AHA_0596,AHA_0608,AHA_0859,AHA_0860,AHA_2305,AHA_2333,AHA_2490,AHA_2537,AHA_2715,AHA_2743,AHA_2982,AHA_2983,AHA_2984,AHA_3545,AHA_3877,AHA_3987,AHA_4006,AHA_4068,AHA_4071,AHA_4115,AHA_4285 |
| GO:0006865 | amino acid transport | biological_process | 2 | 2 | 0 | 2.45 | 0.71375 | 0.908321718 | AHA_0913,AHA_4285 |
| GO:0032774 | RNA biosynthetic process | biological_process | 17 | 6 | 11 | 18.82 | 0.71762 | 0.908321718 | AHA_0290,AHA_0677,AHA_1201,AHA_1213,AHA_1338,AHA_1339,AHA_1522,AHA_1805,AHA_1960,AHA_2538,AHA_2561,AHA_3000,AHA_3561,AHA_3606,AHA_3821,AHA_3874,AHA_4067 |
| GO:0003676 | nucleic acid binding | molecular_function | 23 | 8 | 15 | 25.11 | 0.71785 | 0.908321718 | AHA_0290,AHA_0315,AHA_0677,AHA_1153,AHA_1201,AHA_1213,AHA_1338,AHA_1339,AHA_1522,AHA_1805,AHA_1960,AHA_2423,AHA_2537,AHA_2538,AHA_2561,AHA_2715,AHA_2851,AHA_3000,AHA_3415,AHA_3606,AHA_3821,AHA_4031,AHA_4067 |
| GO:0043170 | macromolecule metabolic process | biological_process | 47 | 27 | 20 | 49.62 | 0.71868 | 0.908321718 | AHA_0262,AHA_0290,AHA_0315,AHA_0612,AHA_0617,AHA_0677,AHA_0724,AHA_0859,AHA_0860,AHA_0988,AHA_1015,AHA_1153,AHA_1172,AHA_1201,AHA_1213,AHA_1338,AHA_1339,AHA_1522,AHA_1805,AHA_1960,AHA_2130,AHA_2163,AHA_2305,AHA_2333,AHA_2405,AHA_2490,AHA_2536,AHA_2538,AHA_2561,AHA_2713,AHA_2851,AHA_2982,AHA_2983,AHA_2984,AHA_3000,AHA_3415,AHA_3561,AHA_3606,AHA_3821,AHA_3874,AHA_3875,AHA_3935,AHA_4031,AHA_4067,AHA_4068,AHA_4071,AHA_4114 |
| GO:0016655 | oxidoreductase activity, acting on NAD(P)H, quinone or similar compound as acceptor | molecular_function | 1 | 1 | 0 | 1.24 | 0.72126 | 0.908321718 | AHA_1770 |
| GO:0071944 | cell periphery | cellular_component | 48 | 37 | 11 | 50.48 | 0.7235 | 0.908321718 | AHA_0021,AHA_0238,AHA_0286,AHA_0452,AHA_0608,AHA_0676,AHA_0847,AHA_0857,AHA_0913,AHA_0952,AHA_0988,AHA_1008,AHA_1246,AHA_1595,AHA_1687,AHA_1713,AHA_1738,AHA_1770,AHA_1964,AHA_2130,AHA_2350,AHA_2452,AHA_2487,AHA_2628,AHA_2637,AHA_2743,AHA_2806,AHA_2809,AHA_2812,AHA_2813,AHA_2835,AHA_2940,AHA_2976,AHA_3004,AHA_3053,AHA_3324,AHA_3379,AHA_3477,AHA_3561,AHA_3604,AHA_3740,AHA_3769,AHA_3820,AHA_3877,AHA_3987,AHA_4261,AHA_4267,AHA_4285 |
| GO:0000910 | cytokinesis | biological_process | 1 | 1 | 0 | 1.25 | 0.72439 | 0.908321718 | AHA_0292 |
| GO:0006643 | membrane lipid metabolic process | biological_process | 1 | 1 | 0 | 1.25 | 0.72439 | 0.908321718 | AHA_0988 |
| GO:0006664 | glycolipid metabolic process | biological_process | 1 | 1 | 0 | 1.25 | 0.72439 | 0.908321718 | AHA_0988 |
| GO:0009247 | glycolipid biosynthetic process | biological_process | 1 | 1 | 0 | 1.25 | 0.72439 | 0.908321718 | AHA_0988 |
| GO:0042180 | cellular ketone metabolic process | biological_process | 1 | 0 | 1 | 1.25 | 0.72439 | 0.908321718 | AHA_4008 |
| GO:0046467 | membrane lipid biosynthetic process | biological_process | 1 | 1 | 0 | 1.25 | 0.72439 | 0.908321718 | AHA_0988 |
| GO:0071103 | DNA conformation change | biological_process | 1 | 0 | 1 | 1.25 | 0.72439 | 0.908321718 | AHA_0262 |
| GO:0071806 | protein transmembrane transport | biological_process | 1 | 1 | 0 | 1.25 | 0.72439 | 0.908321718 | AHA_1738 |
| GO:0005524 | ATP binding | molecular_function | 20 | 15 | 5 | 22.1 | 0.72719 | 0.908321718 | AHA_0238,AHA_0596,AHA_0608,AHA_0859,AHA_0860,AHA_2305,AHA_2333,AHA_2490,AHA_2537,AHA_2715,AHA_2982,AHA_2983,AHA_3545,AHA_3877,AHA_3987,AHA_4006,AHA_4068,AHA_4071,AHA_4115,AHA_4285 |
| GO:0008080 | N-acetyltransferase activity | molecular_function | 2 | 0 | 2 | 2.53 | 0.72917 | 0.908321718 | AHA_0724,AHA_1921 |
| GO:0044237 | cellular metabolic process | biological_process | 84 | 54 | 30 | 86.63 | 0.73353 | 0.908321718 | AHA_0238,AHA_0262,AHA_0278,AHA_0286,AHA_0288,AHA_0290,AHA_0315,AHA_0377,AHA_0378,AHA_0379,AHA_0380,AHA_0481,AHA_0596,AHA_0608,AHA_0677,AHA_0724,AHA_0735,AHA_0803,AHA_0859,AHA_0860,AHA_0988,AHA_1008,AHA_1015,AHA_1040,AHA_1153,AHA_1170,AHA_1201,AHA_1213,AHA_1270,AHA_1338,AHA_1339,AHA_1413,AHA_1522,AHA_1652,AHA_1654,AHA_1770,AHA_1805,AHA_1956,AHA_1960,AHA_2060,AHA_2130,AHA_2305,AHA_2333,AHA_2405,AHA_2490,AHA_2536,AHA_2538,AHA_2561,AHA_2637,AHA_2807,AHA_2851,AHA_2923,AHA_2926,AHA_2982,AHA_2983,AHA_2984,AHA_3000,AHA_3004,AHA_3415,AHA_3545,AHA_3561,AHA_3601,AHA_3606,AHA_3740,AHA_3821,AHA_3874,AHA_3875,AHA_3935,AHA_3949,AHA_3987,AHA_4006,AHA_4008,AHA_4031,AHA_4067,AHA_4068,AHA_4070,AHA_4114,AHA_4115,AHA_4145,AHA_4201,AHA_4258,AHA_4261,AHA_4267,AHA_4285 |
| GO:0004527 | exonuclease activity | molecular_function | 1 | 0 | 1 | 1.29 | 0.73469 | 0.908321718 | AHA_1153 |
| GO:0006418 | tRNA aminoacylation for protein translation | biological_process | 1 | 1 | 0 | 1.3 | 0.73779 | 0.908321718 | AHA_4068 |
| GO:0009081 | branched-chain amino acid metabolic process | biological_process | 1 | 1 | 0 | 1.3 | 0.73779 | 0.908321718 | AHA_4201 |
| GO:0034622 | cellular macromolecular complex assembly | biological_process | 1 | 1 | 0 | 1.3 | 0.73779 | 0.908321718 | AHA_2715 |
| GO:0032559 | adenyl ribonucleotide binding | molecular_function | 20 | 15 | 5 | 22.24 | 0.73822 | 0.908321718 | AHA_0238,AHA_0596,AHA_0608,AHA_0859,AHA_0860,AHA_2305,AHA_2333,AHA_2490,AHA_2537,AHA_2715,AHA_2982,AHA_2983,AHA_3545,AHA_3877,AHA_3987,AHA_4006,AHA_4068,AHA_4071,AHA_4115,AHA_4285 |
| GO:0010556 | regulation of macromolecule biosynthetic process | biological_process | 10 | 4 | 6 | 11.6 | 0.73905 | 0.908321718 | AHA_1338,AHA_1339,AHA_1522,AHA_1960,AHA_2538,AHA_2561,AHA_3000,AHA_3561,AHA_3874,AHA_4031 |
| GO:2000112 | regulation of cellular macromolecule biosynthetic process | biological_process | 10 | 4 | 6 | 11.6 | 0.73905 | 0.908321718 | AHA_1338,AHA_1339,AHA_1522,AHA_1960,AHA_2538,AHA_2561,AHA_3000,AHA_3561,AHA_3874,AHA_4031 |
| GO:0009117 | nucleotide metabolic process | biological_process | 12 | 10 | 2 | 13.76 | 0.73988 | 0.908321718 | AHA_0238,AHA_0278,AHA_0608,AHA_1008,AHA_1413,AHA_3545,AHA_3935,AHA_3987,AHA_4115,AHA_4261,AHA_4267,AHA_4285 |
| GO:0050794 | regulation of cellular process | biological_process | 17 | 6 | 11 | 19.11 | 0.7416 | 0.908321718 | AHA_1015,AHA_1213,AHA_1338,AHA_1339,AHA_1522,AHA_1921,AHA_1960,AHA_2046,AHA_2130,AHA_2536,AHA_2538,AHA_2561,AHA_3000,AHA_3561,AHA_3874,AHA_3935,AHA_4031 |
| GO:0044425 | membrane part | cellular_component | 40 | 28 | 12 | 42.73 | 0.7426 | 0.908321718 | AHA_0021,AHA_0022,AHA_0238,AHA_0286,AHA_0452,AHA_0676,AHA_0847,AHA_0857,AHA_0952,AHA_0988,AHA_1246,AHA_1687,AHA_1713,AHA_1738,AHA_1770,AHA_1921,AHA_2046,AHA_2130,AHA_2350,AHA_2487,AHA_2628,AHA_2637,AHA_2743,AHA_2806,AHA_2809,AHA_2812,AHA_2813,AHA_2940,AHA_2976,AHA_3004,AHA_3053,AHA_3324,AHA_3379,AHA_3561,AHA_3604,AHA_3820,AHA_3987,AHA_4006,AHA_4261,AHA_4267 |
| GO:0015849 | organic acid transport | biological_process | 2 | 2 | 0 | 2.6 | 0.74316 | 0.908321718 | AHA_0913,AHA_4285 |
| GO:0046942 | carboxylic acid transport | biological_process | 2 | 2 | 0 | 2.6 | 0.74316 | 0.908321718 | AHA_0913,AHA_4285 |
| GO:0016020 | membrane | cellular_component | 51 | 36 | 15 | 53.79 | 0.744 | 0.908321718 | AHA_0021,AHA_0022,AHA_0238,AHA_0286,AHA_0452,AHA_0608,AHA_0676,AHA_0847,AHA_0857,AHA_0913,AHA_0952,AHA_0988,AHA_1008,AHA_1055,AHA_1246,AHA_1419,AHA_1687,AHA_1713,AHA_1738,AHA_1770,AHA_1921,AHA_1964,AHA_2046,AHA_2130,AHA_2350,AHA_2487,AHA_2628,AHA_2637,AHA_2743,AHA_2806,AHA_2809,AHA_2812,AHA_2813,AHA_2940,AHA_2976,AHA_3004,AHA_3053,AHA_3324,AHA_3379,AHA_3477,AHA_3561,AHA_3604,AHA_3740,AHA_3769,AHA_3820,AHA_3877,AHA_3987,AHA_4006,AHA_4261,AHA_4267,AHA_4285 |
| GO:0006753 | nucleoside phosphate metabolic process | biological_process | 12 | 10 | 2 | 13.81 | 0.74432 | 0.908321718 | AHA_0238,AHA_0278,AHA_0608,AHA_1008,AHA_1413,AHA_3545,AHA_3935,AHA_3987,AHA_4115,AHA_4261,AHA_4267,AHA_4285 |
| GO:0034645 | cellular macromolecule biosynthetic process | biological_process | 28 | 15 | 13 | 30.66 | 0.74622 | 0.908321718 | AHA_0290,AHA_0315,AHA_0677,AHA_0988,AHA_1015,AHA_1201,AHA_1213,AHA_1338,AHA_1339,AHA_1522,AHA_1805,AHA_1960,AHA_2130,AHA_2333,AHA_2536,AHA_2538,AHA_2561,AHA_2982,AHA_3000,AHA_3561,AHA_3606,AHA_3821,AHA_3874,AHA_3875,AHA_3935,AHA_4031,AHA_4067,AHA_4068 |
| GO:0008081 | phosphoric diester hydrolase activity | molecular_function | 1 | 1 | 0 | 1.34 | 0.74748 | 0.908321718 | AHA_0382 |
| GO:0016769 | transferase activity, transferring nitrogenous groups | molecular_function | 1 | 1 | 0 | 1.34 | 0.74748 | 0.908321718 | AHA_0803 |
| GO:0005618 | cell wall | cellular_component | 1 | 0 | 1 | 1.33 | 0.7475 | 0.908321718 | AHA_2130 |
| GO:0003735 | structural constituent of ribosome | molecular_function | 2 | 2 | 0 | 2.63 | 0.74798 | 0.908321718 | AHA_0315,AHA_4031 |
| GO:0033036 | macromolecule localization | biological_process | 4 | 4 | 0 | 5.01 | 0.74827 | 0.908321718 | AHA_0295,AHA_1738,AHA_3324,AHA_3877 |
| GO:0009889 | regulation of biosynthetic process | biological_process | 10 | 4 | 6 | 11.7 | 0.74855 | 0.908321718 | AHA_1338,AHA_1339,AHA_1522,AHA_1960,AHA_2538,AHA_2561,AHA_3000,AHA_3561,AHA_3874,AHA_4031 |
| GO:0031326 | regulation of cellular biosynthetic process | biological_process | 10 | 4 | 6 | 11.7 | 0.74855 | 0.908321718 | AHA_1338,AHA_1339,AHA_1522,AHA_1960,AHA_2538,AHA_2561,AHA_3000,AHA_3561,AHA_3874,AHA_4031 |
| GO:0016874 | ligase activity | molecular_function | 4 | 3 | 1 | 5.01 | 0.74878 | 0.908321718 | AHA_0596,AHA_2537,AHA_3263,AHA_4068 |
| GO:0043038 | amino acid activation | biological_process | 1 | 1 | 0 | 1.35 | 0.75054 | 0.908321718 | AHA_4068 |
| GO:0043039 | tRNA aminoacylation | biological_process | 1 | 1 | 0 | 1.35 | 0.75054 | 0.908321718 | AHA_4068 |
| GO:0006355 | regulation of transcription, DNA-templated | biological_process | 9 | 3 | 6 | 10.64 | 0.75179 | 0.908321718 | AHA_1338,AHA_1339,AHA_1522,AHA_1960,AHA_2538,AHA_2561,AHA_3000,AHA_3561,AHA_3874 |
| GO:2001141 | regulation of RNA biosynthetic process | biological_process | 9 | 3 | 6 | 10.64 | 0.75179 | 0.908321718 | AHA_1338,AHA_1339,AHA_1522,AHA_1960,AHA_2538,AHA_2561,AHA_3000,AHA_3561,AHA_3874 |
| GO:0015711 | organic anion transport | biological_process | 2 | 2 | 0 | 2.65 | 0.75238 | 0.908321718 | AHA_0913,AHA_4285 |
| GO:0051641 | cellular localization | biological_process | 2 | 2 | 0 | 2.65 | 0.75238 | 0.908321718 | AHA_1738,AHA_3877 |
| GO:0010468 | regulation of gene expression | biological_process | 10 | 4 | 6 | 11.74 | 0.75322 | 0.908321718 | AHA_1338,AHA_1339,AHA_1522,AHA_1960,AHA_2538,AHA_2561,AHA_3000,AHA_3561,AHA_3874,AHA_4031 |
| GO:0016021 | integral component of membrane | cellular_component | 39 | 27 | 12 | 41.89 | 0.7538 | 0.908321718 | AHA_0021,AHA_0022,AHA_0238,AHA_0286,AHA_0452,AHA_0676,AHA_0847,AHA_0857,AHA_0952,AHA_0988,AHA_1246,AHA_1687,AHA_1713,AHA_1738,AHA_1770,AHA_1921,AHA_2046,AHA_2130,AHA_2350,AHA_2487,AHA_2628,AHA_2637,AHA_2743,AHA_2806,AHA_2809,AHA_2812,AHA_2813,AHA_2940,AHA_2976,AHA_3004,AHA_3053,AHA_3324,AHA_3379,AHA_3561,AHA_3604,AHA_3820,AHA_3987,AHA_4006,AHA_4267 |
| GO:0001883 | purine nucleoside binding | molecular_function | 22 | 17 | 5 | 24.63 | 0.75915 | 0.908321718 | AHA_0238,AHA_0278,AHA_0596,AHA_0608,AHA_0859,AHA_0860,AHA_2305,AHA_2333,AHA_2490,AHA_2537,AHA_2715,AHA_2743,AHA_2982,AHA_2983,AHA_3545,AHA_3877,AHA_3987,AHA_4006,AHA_4068,AHA_4071,AHA_4115,AHA_4285 |
| GO:0032550 | purine ribonucleoside binding | molecular_function | 22 | 17 | 5 | 24.63 | 0.75915 | 0.908321718 | AHA_0238,AHA_0278,AHA_0596,AHA_0608,AHA_0859,AHA_0860,AHA_2305,AHA_2333,AHA_2490,AHA_2537,AHA_2715,AHA_2743,AHA_2982,AHA_2983,AHA_3545,AHA_3877,AHA_3987,AHA_4006,AHA_4068,AHA_4071,AHA_4115,AHA_4285 |
| GO:0035639 | purine ribonucleoside triphosphate binding | molecular_function | 22 | 17 | 5 | 24.63 | 0.75915 | 0.908321718 | AHA_0238,AHA_0278,AHA_0596,AHA_0608,AHA_0859,AHA_0860,AHA_2305,AHA_2333,AHA_2490,AHA_2537,AHA_2715,AHA_2743,AHA_2982,AHA_2983,AHA_3545,AHA_3877,AHA_3987,AHA_4006,AHA_4068,AHA_4071,AHA_4115,AHA_4285 |
| GO:0015267 | channel activity | molecular_function | 1 | 1 | 0 | 1.38 | 0.75965 | 0.908321718 | AHA_3053 |
| GO:0022803 | passive transmembrane transporter activity | molecular_function | 1 | 1 | 0 | 1.38 | 0.75965 | 0.908321718 | AHA_3053 |
| GO:0006644 | phospholipid metabolic process | biological_process | 2 | 2 | 0 | 2.7 | 0.76131 | 0.908321718 | AHA_0988,AHA_3740 |
| GO:0051252 | regulation of RNA metabolic process | biological_process | 9 | 3 | 6 | 10.73 | 0.76138 | 0.908321718 | AHA_1338,AHA_1339,AHA_1522,AHA_1960,AHA_2538,AHA_2561,AHA_3000,AHA_3561,AHA_3874 |
| GO:0010467 | gene expression | biological_process | 30 | 16 | 14 | 32.97 | 0.76208 | 0.908321718 | AHA_0290,AHA_0315,AHA_0612,AHA_0617,AHA_0677,AHA_1172,AHA_1201,AHA_1213,AHA_1338,AHA_1339,AHA_1522,AHA_1805,AHA_1960,AHA_2163,AHA_2305,AHA_2538,AHA_2561,AHA_2713,AHA_2851,AHA_3000,AHA_3415,AHA_3561,AHA_3606,AHA_3821,AHA_3874,AHA_4031,AHA_4067,AHA_4068,AHA_4071,AHA_4114 |
| GO:0032549 | ribonucleoside binding | molecular_function | 22 | 17 | 5 | 24.68 | 0.76248 | 0.908321718 | AHA_0238,AHA_0278,AHA_0596,AHA_0608,AHA_0859,AHA_0860,AHA_2305,AHA_2333,AHA_2490,AHA_2537,AHA_2715,AHA_2743,AHA_2982,AHA_2983,AHA_3545,AHA_3877,AHA_3987,AHA_4006,AHA_4068,AHA_4071,AHA_4115,AHA_4285 |
| GO:0006461 | protein complex assembly | biological_process | 1 | 1 | 0 | 1.4 | 0.76268 | 0.908321718 | AHA_0295 |
| GO:0009312 | oligosaccharide biosynthetic process | biological_process | 1 | 1 | 0 | 1.4 | 0.76268 | 0.908321718 | AHA_0988 |
| GO:0070271 | protein complex biogenesis | biological_process | 1 | 1 | 0 | 1.4 | 0.76268 | 0.908321718 | AHA_0295 |
| GO:0009059 | macromolecule biosynthetic process | biological_process | 28 | 15 | 13 | 30.95 | 0.76525 | 0.910353837 | AHA_0290,AHA_0315,AHA_0677,AHA_0988,AHA_1015,AHA_1201,AHA_1213,AHA_1338,AHA_1339,AHA_1522,AHA_1805,AHA_1960,AHA_2130,AHA_2333,AHA_2536,AHA_2538,AHA_2561,AHA_2982,AHA_3000,AHA_3561,AHA_3606,AHA_3821,AHA_3874,AHA_3875,AHA_3935,AHA_4031,AHA_4067,AHA_4068 |
| GO:0008168 | methyltransferase activity | molecular_function | 3 | 0 | 3 | 3.96 | 0.7677 | 0.910757165 | AHA_1956,AHA_2807,AHA_3415 |
| GO:0031224 | intrinsic component of membrane | cellular_component | 39 | 27 | 12 | 42.13 | 0.7693 | 0.910757165 | AHA_0021,AHA_0022,AHA_0238,AHA_0286,AHA_0452,AHA_0676,AHA_0847,AHA_0857,AHA_0952,AHA_0988,AHA_1246,AHA_1687,AHA_1713,AHA_1738,AHA_1770,AHA_1921,AHA_2046,AHA_2130,AHA_2350,AHA_2487,AHA_2628,AHA_2637,AHA_2743,AHA_2806,AHA_2809,AHA_2812,AHA_2813,AHA_2940,AHA_2976,AHA_3004,AHA_3053,AHA_3324,AHA_3379,AHA_3561,AHA_3604,AHA_3820,AHA_3987,AHA_4006,AHA_4267 |
| GO:0016866 | intramolecular transferase activity | molecular_function | 1 | 1 | 0 | 1.43 | 0.77124 | 0.910757165 | AHA_2851 |
| GO:0016746 | transferase activity, transferring acyl groups | molecular_function | 5 | 3 | 2 | 6.35 | 0.7723 | 0.910757165 | AHA_0724,AHA_1040,AHA_1170,AHA_1921,AHA_3740 |
| GO:0006935 | chemotaxis | biological_process | 3 | 3 | 0 | 3.99 | 0.77296 | 0.910757165 | AHA_1391,AHA_3324,AHA_3561 |
| GO:0042330 | taxis | biological_process | 3 | 3 | 0 | 3.99 | 0.77296 | 0.910757165 | AHA_1391,AHA_3324,AHA_3561 |
| GO:0006259 | DNA metabolic process | biological_process | 5 | 3 | 2 | 6.35 | 0.77338 | 0.910757165 | AHA_0262,AHA_1153,AHA_2333,AHA_2982,AHA_3875 |
| GO:0000287 | magnesium ion binding | molecular_function | 2 | 2 | 0 | 2.77 | 0.77411 | 0.910757165 | AHA_3603,AHA_3875 |
| GO:0005525 | GTP binding | molecular_function | 2 | 2 | 0 | 2.77 | 0.77411 | 0.910757165 | AHA_0278,AHA_2743 |
| GO:0032259 | methylation | biological_process | 1 | 0 | 1 | 1.44 | 0.77423 | 0.910757165 | AHA_3415 |
| GO:0046872 | metal ion binding | molecular_function | 26 | 18 | 8 | 29.07 | 0.77567 | 0.911433868 | AHA_0238,AHA_0377,AHA_0378,AHA_1008,AHA_1110,AHA_1172,AHA_1331,AHA_1834,AHA_1948,AHA_1956,AHA_2163,AHA_2405,AHA_2713,AHA_2744,AHA_2807,AHA_2982,AHA_3125,AHA_3263,AHA_3601,AHA_3603,AHA_3728,AHA_3874,AHA_3875,AHA_3877,AHA_4070,AHA_4114 |
| GO:0009451 | RNA modification | biological_process | 2 | 1 | 1 | 2.79 | 0.77835 | 0.911897378 | AHA_2851,AHA_3415 |
| GO:0051301 | cell division | biological_process | 2 | 2 | 0 | 2.79 | 0.77835 | 0.911897378 | AHA_0292,AHA_3935 |
| GO:0032555 | purine ribonucleotide binding | molecular_function | 22 | 17 | 5 | 24.92 | 0.77866 | 0.911897378 | AHA_0238,AHA_0278,AHA_0596,AHA_0608,AHA_0859,AHA_0860,AHA_2305,AHA_2333,AHA_2490,AHA_2537,AHA_2715,AHA_2743,AHA_2982,AHA_2983,AHA_3545,AHA_3877,AHA_3987,AHA_4006,AHA_4068,AHA_4071,AHA_4115,AHA_4285 |
| GO:0051716 | cellular response to stimulus | biological_process | 13 | 7 | 6 | 15.3 | 0.77983 | 0.912083097 | AHA_0290,AHA_0382,AHA_0735,AHA_1153,AHA_1213,AHA_1338,AHA_1413,AHA_2046,AHA_2744,AHA_3004,AHA_3875,AHA_4006,AHA_4008 |
| GO:0032553 | ribonucleotide binding | molecular_function | 23 | 18 | 5 | 26.02 | 0.78155 | 0.912083097 | AHA_0238,AHA_0278,AHA_0596,AHA_0608,AHA_0735,AHA_0859,AHA_0860,AHA_2305,AHA_2333,AHA_2490,AHA_2537,AHA_2715,AHA_2743,AHA_2982,AHA_2983,AHA_3545,AHA_3877,AHA_3987,AHA_4006,AHA_4068,AHA_4071,AHA_4115,AHA_4285 |
| GO:0001882 | nucleoside binding | molecular_function | 22 | 17 | 5 | 24.97 | 0.78181 | 0.912083097 | AHA_0238,AHA_0278,AHA_0596,AHA_0608,AHA_0859,AHA_0860,AHA_2305,AHA_2333,AHA_2490,AHA_2537,AHA_2715,AHA_2743,AHA_2982,AHA_2983,AHA_3545,AHA_3877,AHA_3987,AHA_4006,AHA_4068,AHA_4071,AHA_4115,AHA_4285 |
| GO:0016620 | oxidoreductase activity, acting on the aldehyde or oxo group of donors, NAD or NADP as acceptor | molecular_function | 1 | 1 | 0 | 1.48 | 0.78228 | 0.912083097 | AHA_1248 |
| GO:0044271 | cellular nitrogen compound biosynthetic process | biological_process | 27 | 15 | 12 | 30.22 | 0.78529 | 0.91458084 | AHA_0238,AHA_0290,AHA_0677,AHA_0803,AHA_1201,AHA_1213,AHA_1270,AHA_1338,AHA_1339,AHA_1413,AHA_1522,AHA_1805,AHA_1960,AHA_2538,AHA_2561,AHA_2923,AHA_2926,AHA_3000,AHA_3561,AHA_3606,AHA_3821,AHA_3874,AHA_3935,AHA_4067,AHA_4068,AHA_4261,AHA_4267 |
| GO:0043412 | macromolecule modification | biological_process | 5 | 2 | 3 | 6.5 | 0.79021 | 0.916153333 | AHA_0724,AHA_0988,AHA_2405,AHA_2851,AHA_3415 |
| GO:0050789 | regulation of biological process | biological_process | 17 | 6 | 11 | 19.78 | 0.7925 | 0.916153333 | AHA_1015,AHA_1213,AHA_1338,AHA_1339,AHA_1522,AHA_1921,AHA_1960,AHA_2046,AHA_2130,AHA_2536,AHA_2538,AHA_2561,AHA_3000,AHA_3561,AHA_3874,AHA_3935,AHA_4031 |
| GO:0008173 | RNA methyltransferase activity | molecular_function | 1 | 0 | 1 | 1.53 | 0.79279 | 0.916153333 | AHA_3415 |
| GO:0006364 | rRNA processing | biological_process | 1 | 1 | 0 | 1.54 | 0.79568 | 0.916153333 | AHA_2851 |
| GO:0016072 | rRNA metabolic process | biological_process | 1 | 1 | 0 | 1.54 | 0.79568 | 0.916153333 | AHA_2851 |
| GO:0048519 | negative regulation of biological process | biological_process | 1 | 0 | 1 | 1.54 | 0.79568 | 0.916153333 | AHA_1921 |
| GO:0048523 | negative regulation of cellular process | biological_process | 1 | 0 | 1 | 1.54 | 0.79568 | 0.916153333 | AHA_1921 |
| GO:0072524 | pyridine-containing compound metabolic process | biological_process | 1 | 1 | 0 | 1.54 | 0.79568 | 0.916153333 | AHA_0803 |
| GO:0031323 | regulation of cellular metabolic process | biological_process | 10 | 4 | 6 | 12.22 | 0.79669 | 0.916153333 | AHA_1338,AHA_1339,AHA_1522,AHA_1960,AHA_2538,AHA_2561,AHA_3000,AHA_3561,AHA_3874,AHA_4031 |
| GO:0060255 | regulation of macromolecule metabolic process | biological_process | 10 | 4 | 6 | 12.22 | 0.79669 | 0.916153333 | AHA_1338,AHA_1339,AHA_1522,AHA_1960,AHA_2538,AHA_2561,AHA_3000,AHA_3561,AHA_3874,AHA_4031 |
| GO:0019219 | regulation of nucleobase-containing compound metabolic process | biological_process | 9 | 3 | 6 | 11.12 | 0.79715 | 0.916153333 | AHA_1338,AHA_1339,AHA_1522,AHA_1960,AHA_2538,AHA_2561,AHA_3000,AHA_3561,AHA_3874 |
| GO:0032561 | guanyl ribonucleotide binding | molecular_function | 2 | 2 | 0 | 2.91 | 0.79786 | 0.916153333 | AHA_0278,AHA_2743 |
| GO:0044711 | single-organism biosynthetic process | biological_process | 24 | 17 | 7 | 27.29 | 0.79794 | 0.916153333 | AHA_0238,AHA_0596,AHA_0803,AHA_0988,AHA_1015,AHA_1040,AHA_1170,AHA_1270,AHA_1413,AHA_1654,AHA_1956,AHA_2130,AHA_2536,AHA_2807,AHA_2923,AHA_2926,AHA_3601,AHA_3740,AHA_3935,AHA_3949,AHA_4068,AHA_4201,AHA_4261,AHA_4267 |
| GO:0080090 | regulation of primary metabolic process | biological_process | 10 | 4 | 6 | 12.27 | 0.80072 | 0.917723059 | AHA_1338,AHA_1339,AHA_1522,AHA_1960,AHA_2538,AHA_2561,AHA_3000,AHA_3561,AHA_3874,AHA_4031 |
| GO:0051171 | regulation of nitrogen compound metabolic process | biological_process | 9 | 3 | 6 | 11.17 | 0.80134 | 0.917723059 | AHA_1338,AHA_1339,AHA_1522,AHA_1960,AHA_2538,AHA_2561,AHA_3000,AHA_3561,AHA_3874 |
| GO:0006631 | fatty acid metabolic process | biological_process | 2 | 1 | 1 | 2.94 | 0.80193 | 0.917723059 | AHA_1040,AHA_2060 |
| GO:0020037 | heme binding | molecular_function | 1 | 1 | 0 | 1.58 | 0.80279 | 0.917723059 | AHA_2296 |
| GO:0019001 | guanyl nucleotide binding | molecular_function | 2 | 2 | 0 | 2.96 | 0.80528 | 0.918976212 | AHA_0278,AHA_2743 |
| GO:0022402 | cell cycle process | biological_process | 1 | 1 | 0 | 1.59 | 0.80563 | 0.918976212 | AHA_0292 |
| GO:0005886 | plasma membrane | cellular_component | 44 | 34 | 10 | 47.86 | 0.8085 | 0.920163603 | AHA_0021,AHA_0238,AHA_0286,AHA_0452,AHA_0608,AHA_0676,AHA_0847,AHA_0857,AHA_0913,AHA_0952,AHA_0988,AHA_1008,AHA_1246,AHA_1687,AHA_1713,AHA_1738,AHA_1770,AHA_1964,AHA_2130,AHA_2350,AHA_2487,AHA_2628,AHA_2637,AHA_2743,AHA_2806,AHA_2809,AHA_2812,AHA_2813,AHA_2940,AHA_2976,AHA_3004,AHA_3053,AHA_3324,AHA_3379,AHA_3477,AHA_3561,AHA_3604,AHA_3740,AHA_3820,AHA_3877,AHA_3987,AHA_4261,AHA_4267,AHA_4285 |
| GO:0008643 | carbohydrate transport | biological_process | 2 | 0 | 2 | 2.98 | 0.80929 | 0.920163603 | AHA_1419,AHA_4006 |
| GO:0019318 | hexose metabolic process | biological_process | 2 | 1 | 1 | 2.98 | 0.80929 | 0.920163603 | AHA_2338,AHA_4145 |
| GO:0016616 | oxidoreductase activity, acting on the CH-OH group of donors, NAD or NADP as acceptor | molecular_function | 2 | 1 | 1 | 3.01 | 0.81245 | 0.922761099 | AHA_1331,AHA_4145 |
| GO:0097367 | carbohydrate derivative binding | molecular_function | 23 | 18 | 5 | 26.54 | 0.81385 | 0.923356189 | AHA_0238,AHA_0278,AHA_0596,AHA_0608,AHA_0735,AHA_0859,AHA_0860,AHA_2305,AHA_2333,AHA_2490,AHA_2537,AHA_2715,AHA_2743,AHA_2982,AHA_2983,AHA_3545,AHA_3877,AHA_3987,AHA_4006,AHA_4068,AHA_4071,AHA_4115,AHA_4285 |
| GO:0071822 | protein complex subunit organization | biological_process | 1 | 1 | 0 | 1.64 | 0.81511 | 0.923791333 | AHA_0295 |
| GO:0034654 | nucleobase-containing compound biosynthetic process | biological_process | 22 | 10 | 12 | 25.51 | 0.81794 | 0.925699249 | AHA_0238,AHA_0290,AHA_0677,AHA_1201,AHA_1213,AHA_1338,AHA_1339,AHA_1413,AHA_1522,AHA_1805,AHA_1960,AHA_2538,AHA_2561,AHA_3000,AHA_3561,AHA_3606,AHA_3821,AHA_3874,AHA_3935,AHA_4067,AHA_4261,AHA_4267 |
| GO:0006396 | RNA processing | biological_process | 3 | 1 | 2 | 4.33 | 0.81855 | 0.925699249 | AHA_2305,AHA_2851,AHA_3415 |
| GO:0016070 | RNA metabolic process | biological_process | 21 | 8 | 13 | 24.5 | 0.82106 | 0.926036759 | AHA_0290,AHA_0677,AHA_1201,AHA_1213,AHA_1338,AHA_1339,AHA_1522,AHA_1805,AHA_1960,AHA_2305,AHA_2538,AHA_2561,AHA_2851,AHA_3000,AHA_3415,AHA_3561,AHA_3606,AHA_3821,AHA_3874,AHA_4067,AHA_4068 |
| GO:0004386 | helicase activity | molecular_function | 1 | 1 | 0 | 1.67 | 0.82138 | 0.926036759 | AHA_2715 |
| GO:0043231 | intracellular membrane-bounded organelle | cellular_component | 2 | 0 | 2 | 3.06 | 0.8216 | 0.926036759 | AHA_1921,AHA_2046 |
| GO:0015980 | energy derivation by oxidation of organic compounds | biological_process | 2 | 2 | 0 | 3.08 | 0.82328 | 0.926036759 | AHA_1770,AHA_4145 |
| GO:0019438 | aromatic compound biosynthetic process | biological_process | 26 | 13 | 13 | 29.84 | 0.82382 | 0.926036759 | AHA_0238,AHA_0290,AHA_0677,AHA_0803,AHA_1201,AHA_1213,AHA_1338,AHA_1339,AHA_1413,AHA_1522,AHA_1654,AHA_1805,AHA_1960,AHA_2538,AHA_2561,AHA_2923,AHA_2926,AHA_3000,AHA_3561,AHA_3606,AHA_3821,AHA_3874,AHA_3935,AHA_4067,AHA_4261,AHA_4267 |
| GO:0009991 | response to extracellular stimulus | biological_process | 1 | 0 | 1 | 1.68 | 0.82412 | 0.926036759 | AHA_2405 |
| GO:0016741 | transferase activity, transferring one-carbon groups | molecular_function | 3 | 0 | 3 | 4.39 | 0.8254 | 0.926487327 | AHA_1956,AHA_2807,AHA_3415 |
| GO:0008152 | metabolic process | biological_process | 94 | 60 | 34 | 97.8 | 0.82734 | 0.927676979 | AHA_0238,AHA_0262,AHA_0278,AHA_0286,AHA_0288,AHA_0290,AHA_0315,AHA_0377,AHA_0378,AHA_0379,AHA_0380,AHA_0481,AHA_0596,AHA_0608,AHA_0612,AHA_0617,AHA_0677,AHA_0724,AHA_0735,AHA_0803,AHA_0859,AHA_0860,AHA_0988,AHA_1008,AHA_1015,AHA_1040,AHA_1153,AHA_1170,AHA_1172,AHA_1201,AHA_1213,AHA_1248,AHA_1270,AHA_1338,AHA_1339,AHA_1413,AHA_1522,AHA_1652,AHA_1654,AHA_1770,AHA_1805,AHA_1834,AHA_1956,AHA_1960,AHA_2060,AHA_2093,AHA_2130,AHA_2163,AHA_2305,AHA_2333,AHA_2338,AHA_2405,AHA_2490,AHA_2536,AHA_2538,AHA_2561,AHA_2637,AHA_2713,AHA_2807,AHA_2851,AHA_2923,AHA_2926,AHA_2982,AHA_2983,AHA_2984,AHA_3000,AHA_3004,AHA_3415,AHA_3545,AHA_3561,AHA_3601,AHA_3606,AHA_3740,AHA_3821,AHA_3874,AHA_3875,AHA_3935,AHA_3949,AHA_3987,AHA_4006,AHA_4008,AHA_4031,AHA_4067,AHA_4068,AHA_4070,AHA_4071,AHA_4114,AHA_4115,AHA_4145,AHA_4201,AHA_4258,AHA_4261,AHA_4267,AHA_4285 |
| GO:0046906 | tetrapyrrole binding | molecular_function | 1 | 1 | 0 | 1.72 | 0.83002 | 0.927765116 | AHA_2296 |
| GO:0044763 | single-organism cellular process | biological_process | 66 | 47 | 19 | 70.7 | 0.83076 | 0.927765116 | AHA_0022,AHA_0238,AHA_0278,AHA_0286,AHA_0288,AHA_0290,AHA_0292,AHA_0377,AHA_0378,AHA_0379,AHA_0380,AHA_0481,AHA_0596,AHA_0608,AHA_0803,AHA_0847,AHA_0988,AHA_1008,AHA_1015,AHA_1040,AHA_1110,AHA_1153,AHA_1170,AHA_1213,AHA_1270,AHA_1338,AHA_1413,AHA_1652,AHA_1654,AHA_1713,AHA_1738,AHA_1770,AHA_1921,AHA_1956,AHA_2046,AHA_2060,AHA_2130,AHA_2405,AHA_2536,AHA_2637,AHA_2744,AHA_2807,AHA_2812,AHA_2835,AHA_2837,AHA_2923,AHA_2926,AHA_3053,AHA_3545,AHA_3601,AHA_3604,AHA_3728,AHA_3740,AHA_3875,AHA_3935,AHA_3949,AHA_3987,AHA_4006,AHA_4008,AHA_4068,AHA_4115,AHA_4145,AHA_4201,AHA_4261,AHA_4267,AHA_4285 |
| GO:0043169 | cation binding | molecular_function | 26 | 18 | 8 | 30.03 | 0.8309 | 0.927765116 | AHA_0238,AHA_0377,AHA_0378,AHA_1008,AHA_1110,AHA_1172,AHA_1331,AHA_1834,AHA_1948,AHA_1956,AHA_2163,AHA_2405,AHA_2713,AHA_2744,AHA_2807,AHA_2982,AHA_3125,AHA_3263,AHA_3601,AHA_3603,AHA_3728,AHA_3874,AHA_3875,AHA_3877,AHA_4070,AHA_4114 |
| GO:0044249 | cellular biosynthetic process | biological_process | 47 | 29 | 18 | 51.69 | 0.83224 | 0.927765116 | AHA_0238,AHA_0290,AHA_0315,AHA_0596,AHA_0677,AHA_0803,AHA_0988,AHA_1015,AHA_1040,AHA_1170,AHA_1201,AHA_1213,AHA_1270,AHA_1338,AHA_1339,AHA_1413,AHA_1522,AHA_1654,AHA_1805,AHA_1956,AHA_1960,AHA_2130,AHA_2333,AHA_2536,AHA_2538,AHA_2561,AHA_2807,AHA_2923,AHA_2926,AHA_2982,AHA_3000,AHA_3561,AHA_3601,AHA_3606,AHA_3740,AHA_3821,AHA_3874,AHA_3875,AHA_3935,AHA_3949,AHA_4031,AHA_4067,AHA_4068,AHA_4070,AHA_4201,AHA_4261,AHA_4267 |
| GO:0006633 | fatty acid biosynthetic process | biological_process | 1 | 1 | 0 | 1.73 | 0.8327 | 0.927765116 | AHA_1040 |
| GO:0019835 | cytolysis | biological_process | 1 | 1 | 0 | 1.73 | 0.8327 | 0.927765116 | AHA_0292 |
| GO:0043227 | membrane-bounded organelle | cellular_component | 2 | 0 | 2 | 3.16 | 0.8352 | 0.928886091 | AHA_1921,AHA_2046 |
| GO:0006281 | DNA repair | biological_process | 2 | 1 | 1 | 3.18 | 0.83635 | 0.928886091 | AHA_1153,AHA_3875 |
| GO:0007049 | cell cycle | biological_process | 2 | 2 | 0 | 3.18 | 0.83635 | 0.928886091 | AHA_0292,AHA_3935 |
| GO:0009311 | oligosaccharide metabolic process | biological_process | 1 | 1 | 0 | 1.78 | 0.84087 | 0.932923137 | AHA_0988 |
| GO:0016407 | acetyltransferase activity | molecular_function | 2 | 0 | 2 | 3.25 | 0.84492 | 0.934745535 | AHA_0724,AHA_1921 |
| GO:0008236 | serine-type peptidase activity | molecular_function | 1 | 1 | 0 | 1.81 | 0.84606 | 0.934745535 | AHA_0617 |
| GO:0016903 | oxidoreductase activity, acting on the aldehyde or oxo group of donors | molecular_function | 1 | 1 | 0 | 1.81 | 0.84606 | 0.934745535 | AHA_1248 |
| GO:0017171 | serine hydrolase activity | molecular_function | 1 | 1 | 0 | 1.81 | 0.84606 | 0.934745535 | AHA_0617 |
| GO:0019222 | regulation of metabolic process | biological_process | 10 | 4 | 6 | 12.9 | 0.84792 | 0.935634477 | AHA_1338,AHA_1339,AHA_1522,AHA_1960,AHA_2538,AHA_2561,AHA_3000,AHA_3561,AHA_3874,AHA_4031 |
| GO:0006007 | glucose catabolic process | biological_process | 1 | 1 | 0 | 1.83 | 0.84864 | 0.935634477 | AHA_4145 |
| GO:0019637 | organophosphate metabolic process | biological_process | 15 | 12 | 3 | 18.53 | 0.85139 | 0.937080814 | AHA_0238,AHA_0278,AHA_0608,AHA_0988,AHA_1008,AHA_1413,AHA_1652,AHA_3545,AHA_3740,AHA_3935,AHA_3987,AHA_4115,AHA_4261,AHA_4267,AHA_4285 |
| GO:0055086 | nucleobase-containing small molecule metabolic process | biological_process | 12 | 10 | 2 | 15.21 | 0.85173 | 0.937080814 | AHA_0238,AHA_0278,AHA_0608,AHA_1008,AHA_1413,AHA_3545,AHA_3935,AHA_3987,AHA_4115,AHA_4261,AHA_4267,AHA_4285 |
| GO:0009057 | macromolecule catabolic process | biological_process | 1 | 1 | 0 | 1.88 | 0.85603 | 0.939849604 | AHA_4114 |
| GO:0019320 | hexose catabolic process | biological_process | 1 | 1 | 0 | 1.88 | 0.85603 | 0.939849604 | AHA_4145 |
| GO:0008610 | lipid biosynthetic process | biological_process | 4 | 4 | 0 | 5.97 | 0.85885 | 0.941964516 | AHA_0988,AHA_1040,AHA_3601,AHA_3740 |
| GO:0090304 | nucleic acid metabolic process | biological_process | 26 | 11 | 15 | 30.61 | 0.86251 | 0.944995364 | AHA_0262,AHA_0290,AHA_0677,AHA_1153,AHA_1201,AHA_1213,AHA_1338,AHA_1339,AHA_1522,AHA_1805,AHA_1960,AHA_2305,AHA_2333,AHA_2538,AHA_2561,AHA_2851,AHA_2982,AHA_3000,AHA_3415,AHA_3561,AHA_3606,AHA_3821,AHA_3874,AHA_3875,AHA_4067,AHA_4068 |
| GO:0009605 | response to external stimulus | biological_process | 4 | 3 | 1 | 6.06 | 0.86724 | 0.947758984 | AHA_1391,AHA_2405,AHA_3324,AHA_3561 |
| GO:0008757 | S-adenosylmethionine-dependent methyltransferase activity | molecular_function | 1 | 0 | 1 | 1.96 | 0.86735 | 0.947758984 | AHA_2807 |
| GO:0005488 | binding | molecular_function | 76 | 50 | 26 | 81.72 | 0.86773 | 0.947758984 | AHA_0238,AHA_0278,AHA_0290,AHA_0315,AHA_0377,AHA_0378,AHA_0596,AHA_0608,AHA_0677,AHA_0735,AHA_0859,AHA_0860,AHA_1008,AHA_1110,AHA_1153,AHA_1172,AHA_1201,AHA_1213,AHA_1331,AHA_1338,AHA_1339,AHA_1522,AHA_1770,AHA_1805,AHA_1834,AHA_1948,AHA_1956,AHA_1960,AHA_2163,AHA_2296,AHA_2305,AHA_2333,AHA_2405,AHA_2423,AHA_2490,AHA_2537,AHA_2538,AHA_2561,AHA_2713,AHA_2715,AHA_2743,AHA_2744,AHA_2807,AHA_2809,AHA_2851,AHA_2982,AHA_2983,AHA_2984,AHA_3000,AHA_3125,AHA_3263,AHA_3324,AHA_3415,AHA_3545,AHA_3601,AHA_3603,AHA_3606,AHA_3728,AHA_3821,AHA_3874,AHA_3875,AHA_3877,AHA_3987,AHA_4006,AHA_4031,AHA_4067,AHA_4068,AHA_4070,AHA_4071,AHA_4114,AHA_4115,AHA_4145,AHA_4201,AHA_4258,AHA_4267,AHA_4285 |
| GO:0044710 | single-organism metabolic process | biological_process | 53 | 35 | 18 | 58.72 | 0.87258 | 0.951215926 | AHA_0238,AHA_0278,AHA_0286,AHA_0288,AHA_0290,AHA_0377,AHA_0378,AHA_0379,AHA_0380,AHA_0481,AHA_0596,AHA_0608,AHA_0803,AHA_0988,AHA_1008,AHA_1015,AHA_1040,AHA_1153,AHA_1170,AHA_1213,AHA_1248,AHA_1270,AHA_1413,AHA_1652,AHA_1654,AHA_1770,AHA_1956,AHA_2060,AHA_2093,AHA_2130,AHA_2338,AHA_2405,AHA_2536,AHA_2637,AHA_2807,AHA_2923,AHA_2926,AHA_3545,AHA_3601,AHA_3740,AHA_3875,AHA_3935,AHA_3949,AHA_3987,AHA_4006,AHA_4008,AHA_4068,AHA_4115,AHA_4145,AHA_4201,AHA_4261,AHA_4267,AHA_4285 |
| GO:0043168 | anion binding | molecular_function | 28 | 22 | 6 | 33.03 | 0.8727 | 0.951215926 | AHA_0238,AHA_0278,AHA_0596,AHA_0608,AHA_0735,AHA_0859,AHA_0860,AHA_2305,AHA_2333,AHA_2405,AHA_2490,AHA_2537,AHA_2715,AHA_2743,AHA_2809,AHA_2982,AHA_2983,AHA_3545,AHA_3877,AHA_3987,AHA_4006,AHA_4068,AHA_4071,AHA_4115,AHA_4145,AHA_4201,AHA_4258,AHA_4285 |
| GO:0051287 | NAD binding | molecular_function | 1 | 1 | 0 | 2 | 0.87377 | 0.951398326 | AHA_4145 |
| GO:0044255 | cellular lipid metabolic process | biological_process | 5 | 4 | 1 | 7.41 | 0.87531 | 0.952091579 | AHA_0988,AHA_1040,AHA_2060,AHA_3601,AHA_3740 |
| GO:0016757 | transferase activity, transferring glycosyl groups | molecular_function | 2 | 1 | 1 | 3.53 | 0.87715 | 0.953035041 | AHA_0988,AHA_2130 |
| GO:0009165 | nucleotide biosynthetic process | biological_process | 5 | 4 | 1 | 7.46 | 0.87885 | 0.953035041 | AHA_0238,AHA_1413,AHA_3935,AHA_4261,AHA_4267 |
| GO:0071702 | organic substance transport | biological_process | 8 | 6 | 2 | 11.07 | 0.87889 | 0.953035041 | AHA_0295,AHA_0913,AHA_1419,AHA_1738,AHA_3324,AHA_3877,AHA_4006,AHA_4285 |
| GO:0009058 | biosynthetic process | biological_process | 48 | 30 | 18 | 53.86 | 0.87999 | 0.953247133 | AHA_0238,AHA_0290,AHA_0315,AHA_0380,AHA_0596,AHA_0677,AHA_0803,AHA_0988,AHA_1015,AHA_1040,AHA_1170,AHA_1201,AHA_1213,AHA_1270,AHA_1338,AHA_1339,AHA_1413,AHA_1522,AHA_1654,AHA_1805,AHA_1956,AHA_1960,AHA_2130,AHA_2333,AHA_2536,AHA_2538,AHA_2561,AHA_2807,AHA_2923,AHA_2926,AHA_2982,AHA_3000,AHA_3561,AHA_3601,AHA_3606,AHA_3740,AHA_3821,AHA_3874,AHA_3875,AHA_3935,AHA_3949,AHA_4031,AHA_4067,AHA_4068,AHA_4070,AHA_4201,AHA_4261,AHA_4267 |
| GO:0022607 | cellular component assembly | biological_process | 2 | 2 | 0 | 3.61 | 0.88504 | 0.956461803 | AHA_0295,AHA_2715 |
| GO:0043167 | ion binding | molecular_function | 50 | 38 | 12 | 56.23 | 0.88568 | 0.956461803 | AHA_0238,AHA_0278,AHA_0377,AHA_0378,AHA_0596,AHA_0608,AHA_0735,AHA_0859,AHA_0860,AHA_1008,AHA_1110,AHA_1172,AHA_1331,AHA_1834,AHA_1948,AHA_1956,AHA_2163,AHA_2305,AHA_2333,AHA_2405,AHA_2490,AHA_2537,AHA_2713,AHA_2715,AHA_2743,AHA_2744,AHA_2807,AHA_2809,AHA_2982,AHA_2983,AHA_3125,AHA_3263,AHA_3545,AHA_3601,AHA_3603,AHA_3728,AHA_3874,AHA_3875,AHA_3877,AHA_3987,AHA_4006,AHA_4068,AHA_4070,AHA_4071,AHA_4114,AHA_4115,AHA_4145,AHA_4201,AHA_4258,AHA_4285 |
| GO:1901293 | nucleoside phosphate biosynthetic process | biological_process | 5 | 4 | 1 | 7.56 | 0.88568 | 0.956461803 | AHA_0238,AHA_1413,AHA_3935,AHA_4261,AHA_4267 |
| GO:0006139 | nucleobase-containing compound metabolic process | biological_process | 38 | 21 | 17 | 43.89 | 0.8907 | 0.959980613 | AHA_0238,AHA_0262,AHA_0278,AHA_0290,AHA_0608,AHA_0677,AHA_1008,AHA_1153,AHA_1201,AHA_1213,AHA_1338,AHA_1339,AHA_1413,AHA_1522,AHA_1805,AHA_1960,AHA_2305,AHA_2333,AHA_2538,AHA_2561,AHA_2851,AHA_2982,AHA_3000,AHA_3415,AHA_3545,AHA_3561,AHA_3606,AHA_3821,AHA_3874,AHA_3875,AHA_3935,AHA_3987,AHA_4067,AHA_4068,AHA_4115,AHA_4261,AHA_4267,AHA_4285 |
| GO:0034660 | ncRNA metabolic process | biological_process | 3 | 2 | 1 | 5.05 | 0.89076 | 0.959980613 | AHA_2305,AHA_2851,AHA_4068 |
| GO:0006399 | tRNA metabolic process | biological_process | 2 | 1 | 1 | 3.71 | 0.89387 | 0.962348294 | AHA_2305,AHA_4068 |
| GO:1901576 | organic substance biosynthetic process | biological_process | 46 | 28 | 18 | 52.27 | 0.89571 | 0.963345245 | AHA_0238,AHA_0290,AHA_0315,AHA_0596,AHA_0677,AHA_0803,AHA_0988,AHA_1015,AHA_1040,AHA_1170,AHA_1201,AHA_1213,AHA_1270,AHA_1338,AHA_1339,AHA_1413,AHA_1522,AHA_1654,AHA_1805,AHA_1956,AHA_1960,AHA_2130,AHA_2333,AHA_2536,AHA_2538,AHA_2561,AHA_2807,AHA_2923,AHA_2926,AHA_2982,AHA_3000,AHA_3561,AHA_3601,AHA_3606,AHA_3740,AHA_3821,AHA_3874,AHA_3875,AHA_3935,AHA_3949,AHA_4031,AHA_4067,AHA_4068,AHA_4201,AHA_4261,AHA_4267 |
| GO:0034470 | ncRNA processing | biological_process | 2 | 1 | 1 | 3.75 | 0.89805 | 0.96487737 | AHA_2305,AHA_2851 |
| GO:0006412 | translation | biological_process | 3 | 3 | 0 | 5.2 | 0.90169 | 0.965938621 | AHA_0315,AHA_4031,AHA_4068 |
| GO:1901362 | organic cyclic compound biosynthetic process | biological_process | 26 | 13 | 13 | 31.62 | 0.90339 | 0.965938621 | AHA_0238,AHA_0290,AHA_0677,AHA_0803,AHA_1201,AHA_1213,AHA_1338,AHA_1339,AHA_1413,AHA_1522,AHA_1654,AHA_1805,AHA_1960,AHA_2538,AHA_2561,AHA_2923,AHA_2926,AHA_3000,AHA_3561,AHA_3606,AHA_3821,AHA_3874,AHA_3935,AHA_4067,AHA_4261,AHA_4267 |
| GO:0008653 | lipopolysaccharide metabolic process | biological_process | 1 | 1 | 0 | 2.26 | 0.90362 | 0.965938621 | AHA_0988 |
| GO:0009103 | lipopolysaccharide biosynthetic process | biological_process | 1 | 1 | 0 | 2.26 | 0.90362 | 0.965938621 | AHA_0988 |
| GO:0072330 | monocarboxylic acid biosynthetic process | biological_process | 1 | 1 | 0 | 2.26 | 0.90362 | 0.965938621 | AHA_1040 |
| GO:0016651 | oxidoreductase activity, acting on NAD(P)H | molecular_function | 1 | 1 | 0 | 2.29 | 0.90632 | 0.967843242 | AHA_1770 |
| GO:0016491 | oxidoreductase activity | molecular_function | 13 | 9 | 4 | 17.47 | 0.90844 | 0.969125263 | AHA_0735,AHA_1110,AHA_1248,AHA_1331,AHA_1652,AHA_1770,AHA_1948,AHA_2296,AHA_2333,AHA_2405,AHA_3601,AHA_3949,AHA_4145 |
| GO:0018130 | heterocycle biosynthetic process | biological_process | 25 | 13 | 12 | 30.95 | 0.91681 | 0.976397919 | AHA_0238,AHA_0290,AHA_0677,AHA_0803,AHA_1201,AHA_1213,AHA_1338,AHA_1339,AHA_1413,AHA_1522,AHA_1805,AHA_1960,AHA_2538,AHA_2561,AHA_2923,AHA_2926,AHA_3000,AHA_3561,AHA_3606,AHA_3821,AHA_3874,AHA_3935,AHA_4067,AHA_4261,AHA_4267 |
| GO:0008033 | tRNA processing | biological_process | 1 | 0 | 1 | 2.41 | 0.91711 | 0.976397919 | AHA_2305 |
| GO:0009055 | electron carrier activity | molecular_function | 2 | 1 | 1 | 4.06 | 0.92081 | 0.977077048 | AHA_2093,AHA_2296 |
| GO:0006006 | glucose metabolic process | biological_process | 1 | 1 | 0 | 2.45 | 0.92118 | 0.977077048 | AHA_4145 |
| GO:0045333 | cellular respiration | biological_process | 1 | 1 | 0 | 2.45 | 0.92118 | 0.977077048 | AHA_1770 |
| GO:0006629 | lipid metabolic process | biological_process | 5 | 4 | 1 | 8.18 | 0.92257 | 0.977077048 | AHA_0988,AHA_1040,AHA_2060,AHA_3601,AHA_3740 |
| GO:0050660 | flavin adenine dinucleotide binding | molecular_function | 1 | 1 | 0 | 2.48 | 0.92324 | 0.977077048 | AHA_2809 |
| GO:0071705 | nitrogen compound transport | biological_process | 2 | 2 | 0 | 4.09 | 0.92331 | 0.977077048 | AHA_0913,AHA_4285 |
| GO:0030170 | pyridoxal phosphate binding | molecular_function | 1 | 1 | 0 | 2.53 | 0.92697 | 0.979966279 | AHA_4201 |
| GO:0044462 | external encapsulating structure part | cellular_component | 5 | 4 | 1 | 8.35 | 0.9318 | 0.98257042 | AHA_1595,AHA_2452,AHA_2835,AHA_3324,AHA_3769 |
| GO:0009110 | vitamin biosynthetic process | biological_process | 1 | 1 | 0 | 2.6 | 0.93223 | 0.98257042 | AHA_0803 |
| GO:0042364 | water-soluble vitamin biosynthetic process | biological_process | 1 | 1 | 0 | 2.6 | 0.93223 | 0.98257042 | AHA_0803 |
| GO:0030312 | external encapsulating structure | cellular_component | 6 | 4 | 2 | 9.68 | 0.9343 | 0.983451012 | AHA_1595,AHA_2130,AHA_2452,AHA_2835,AHA_3324,AHA_3769 |
| GO:0006766 | vitamin metabolic process | biological_process | 1 | 1 | 0 | 2.7 | 0.93873 | 0.983451012 | AHA_0803 |
| GO:0006767 | water-soluble vitamin metabolic process | biological_process | 1 | 1 | 0 | 2.7 | 0.93873 | 0.983451012 | AHA_0803 |
| GO:0033692 | cellular polysaccharide biosynthetic process | biological_process | 1 | 1 | 0 | 2.7 | 0.93873 | 0.983451012 | AHA_0988 |
| GO:0034637 | cellular carbohydrate biosynthetic process | biological_process | 1 | 1 | 0 | 2.7 | 0.93873 | 0.983451012 | AHA_0988 |
| GO:0031975 | envelope | cellular_component | 6 | 4 | 2 | 9.83 | 0.9401 | 0.983451012 | AHA_1595,AHA_1921,AHA_2452,AHA_2835,AHA_3324,AHA_3769 |
| GO:0004518 | nuclease activity | molecular_function | 1 | 0 | 1 | 2.72 | 0.94019 | 0.983451012 | AHA_1153 |
| GO:0000156 | phosphorelay response regulator activity | molecular_function | 2 | 0 | 2 | 4.39 | 0.94053 | 0.983451012 | AHA_1213,AHA_1338 |
| GO:0006796 | phosphate-containing compound metabolic process | biological_process | 16 | 13 | 3 | 22.19 | 0.95028 | 0.992661169 | AHA_0238,AHA_0278,AHA_0608,AHA_0988,AHA_1008,AHA_1413,AHA_1652,AHA_1770,AHA_3545,AHA_3740,AHA_3935,AHA_3987,AHA_4115,AHA_4261,AHA_4267,AHA_4285 |
| GO:0000271 | polysaccharide biosynthetic process | biological_process | 1 | 1 | 0 | 2.94 | 0.95239 | 0.992897191 | AHA_0988 |
| GO:0009405 | pathogenesis | biological_process | 1 | 1 | 0 | 2.94 | 0.95239 | 0.992897191 | AHA_1248 |
| GO:0090407 | organophosphate biosynthetic process | biological_process | 7 | 6 | 1 | 11.6 | 0.95473 | 0.993381994 | AHA_0238,AHA_0988,AHA_1413,AHA_3740,AHA_3935,AHA_4261,AHA_4267 |
| GO:0044264 | cellular polysaccharide metabolic process | biological_process | 1 | 1 | 0 | 2.98 | 0.95474 | 0.993381994 | AHA_0988 |
| GO:0022900 | electron transport chain | biological_process | 2 | 2 | 0 | 4.76 | 0.95728 | 0.994711207 | AHA_1770,AHA_2637 |
| GO:1901363 | heterocyclic compound binding | molecular_function | 51 | 32 | 19 | 60.29 | 0.95907 | 0.994711207 | AHA_0238,AHA_0278,AHA_0290,AHA_0315,AHA_0596,AHA_0608,AHA_0677,AHA_0735,AHA_0859,AHA_0860,AHA_1008,AHA_1153,AHA_1201,AHA_1213,AHA_1338,AHA_1339,AHA_1522,AHA_1805,AHA_1960,AHA_2296,AHA_2305,AHA_2333,AHA_2423,AHA_2490,AHA_2537,AHA_2538,AHA_2561,AHA_2715,AHA_2743,AHA_2809,AHA_2851,AHA_2982,AHA_2983,AHA_2984,AHA_3000,AHA_3415,AHA_3545,AHA_3606,AHA_3821,AHA_3877,AHA_3987,AHA_4006,AHA_4031,AHA_4067,AHA_4068,AHA_4071,AHA_4115,AHA_4145,AHA_4201,AHA_4258,AHA_4285 |
| GO:0097159 | organic cyclic compound binding | molecular_function | 51 | 32 | 19 | 60.39 | 0.96052 | 0.994711207 | AHA_0238,AHA_0278,AHA_0290,AHA_0315,AHA_0596,AHA_0608,AHA_0677,AHA_0735,AHA_0859,AHA_0860,AHA_1008,AHA_1153,AHA_1201,AHA_1213,AHA_1338,AHA_1339,AHA_1522,AHA_1805,AHA_1960,AHA_2296,AHA_2305,AHA_2333,AHA_2423,AHA_2490,AHA_2537,AHA_2538,AHA_2561,AHA_2715,AHA_2743,AHA_2809,AHA_2851,AHA_2982,AHA_2983,AHA_2984,AHA_3000,AHA_3415,AHA_3545,AHA_3606,AHA_3821,AHA_3877,AHA_3987,AHA_4006,AHA_4031,AHA_4067,AHA_4068,AHA_4071,AHA_4115,AHA_4145,AHA_4201,AHA_4258,AHA_4285 |
| GO:0044699 | single-organism process | biological_process | 75 | 52 | 23 | 83.7 | 0.96083 | 0.994711207 | AHA_0022,AHA_0238,AHA_0278,AHA_0286,AHA_0288,AHA_0290,AHA_0292,AHA_0377,AHA_0378,AHA_0379,AHA_0380,AHA_0481,AHA_0596,AHA_0608,AHA_0676,AHA_0803,AHA_0847,AHA_0913,AHA_0988,AHA_1008,AHA_1015,AHA_1040,AHA_1110,AHA_1153,AHA_1170,AHA_1213,AHA_1248,AHA_1270,AHA_1338,AHA_1413,AHA_1419,AHA_1652,AHA_1654,AHA_1713,AHA_1738,AHA_1770,AHA_1921,AHA_1956,AHA_1964,AHA_2046,AHA_2060,AHA_2093,AHA_2130,AHA_2338,AHA_2405,AHA_2536,AHA_2637,AHA_2744,AHA_2807,AHA_2812,AHA_2813,AHA_2835,AHA_2837,AHA_2923,AHA_2926,AHA_3053,AHA_3545,AHA_3601,AHA_3604,AHA_3728,AHA_3740,AHA_3875,AHA_3877,AHA_3935,AHA_3949,AHA_3987,AHA_4006,AHA_4008,AHA_4068,AHA_4115,AHA_4145,AHA_4201,AHA_4261,AHA_4267,AHA_4285 |
| GO:0030313 | cell envelope | cellular_component | 5 | 4 | 1 | 9.19 | 0.9614 | 0.994711207 | AHA_1595,AHA_2452,AHA_2835,AHA_3324,AHA_3769 |
| GO:0016788 | hydrolase activity, acting on ester bonds | molecular_function | 4 | 3 | 1 | 7.88 | 0.96168 | 0.994711207 | AHA_0382,AHA_1008,AHA_1153,AHA_3603 |
| GO:0006793 | phosphorus metabolic process | biological_process | 16 | 13 | 3 | 22.86 | 0.96412 | 0.996257333 | AHA_0238,AHA_0278,AHA_0608,AHA_0988,AHA_1008,AHA_1413,AHA_1652,AHA_1770,AHA_3545,AHA_3740,AHA_3935,AHA_3987,AHA_4115,AHA_4261,AHA_4267,AHA_4285 |
| GO:0048037 | cofactor binding | molecular_function | 6 | 5 | 1 | 10.79 | 0.96636 | 0.997310224 | AHA_0735,AHA_1770,AHA_1834,AHA_2809,AHA_4145,AHA_4201 |
| GO:0036094 | small molecule binding | molecular_function | 29 | 23 | 6 | 37.81 | 0.96867 | 0.997310224 | AHA_0238,AHA_0278,AHA_0596,AHA_0608,AHA_0735,AHA_0859,AHA_0860,AHA_1008,AHA_2305,AHA_2333,AHA_2405,AHA_2490,AHA_2537,AHA_2715,AHA_2743,AHA_2809,AHA_2982,AHA_2983,AHA_2984,AHA_3545,AHA_3877,AHA_3987,AHA_4006,AHA_4068,AHA_4071,AHA_4115,AHA_4145,AHA_4258,AHA_4285 |
| GO:0051704 | multi-organism process | biological_process | 2 | 2 | 0 | 5.15 | 0.96967 | 0.997310224 | AHA_0452,AHA_1248 |
| GO:0000166 | nucleotide binding | molecular_function | 27 | 22 | 5 | 35.71 | 0.96987 | 0.997310224 | AHA_0238,AHA_0278,AHA_0596,AHA_0608,AHA_0735,AHA_0859,AHA_0860,AHA_1008,AHA_2305,AHA_2333,AHA_2490,AHA_2537,AHA_2715,AHA_2743,AHA_2809,AHA_2982,AHA_2983,AHA_2984,AHA_3545,AHA_3877,AHA_3987,AHA_4006,AHA_4068,AHA_4071,AHA_4115,AHA_4145,AHA_4285 |
| GO:1901265 | nucleoside phosphate binding | molecular_function | 27 | 22 | 5 | 35.71 | 0.96987 | 0.997310224 | AHA_0238,AHA_0278,AHA_0596,AHA_0608,AHA_0735,AHA_0859,AHA_0860,AHA_1008,AHA_2305,AHA_2333,AHA_2490,AHA_2537,AHA_2715,AHA_2743,AHA_2809,AHA_2982,AHA_2983,AHA_2984,AHA_3545,AHA_3877,AHA_3987,AHA_4006,AHA_4068,AHA_4071,AHA_4115,AHA_4145,AHA_4285 |
| GO:0016773 | phosphotransferase activity, alcohol group as acceptor | molecular_function | 3 | 1 | 2 | 6.83 | 0.97199 | 0.998516043 | AHA_1419,AHA_3603,AHA_4008 |
| GO:0005976 | polysaccharide metabolic process | biological_process | 1 | 1 | 0 | 3.56 | 0.97536 | 1 | AHA_0988 |
| GO:0016775 | phosphotransferase activity, nitrogenous group as acceptor | molecular_function | 1 | 0 | 1 | 3.58 | 0.97572 | 1 | AHA_4006 |
| GO:0006732 | coenzyme metabolic process | biological_process | 2 | 1 | 1 | 5.53 | 0.97859 | 1 | AHA_3949,AHA_4070 |
| GO:0050662 | coenzyme binding | molecular_function | 3 | 3 | 0 | 7.26 | 0.9803 | 1 | AHA_0735,AHA_2809,AHA_4145 |
| GO:0055114 | oxidation-reduction process | biological_process | 4 | 3 | 1 | 8.95 | 0.98321 | 1 | AHA_1770,AHA_2093,AHA_2637,AHA_4145 |
| GO:0006091 | generation of precursor metabolites and energy | biological_process | 3 | 3 | 0 | 7.56 | 0.98483 | 1 | AHA_1770,AHA_2637,AHA_4145 |
| GO:0016051 | carbohydrate biosynthetic process | biological_process | 1 | 1 | 0 | 4.04 | 0.98519 | 1 | AHA_0988 |
| GO:0051539 | 4 iron, 4 sulfur cluster binding | molecular_function | 1 | 1 | 0 | 4.06 | 0.98533 | 1 | AHA_3263 |
| GO:0016310 | phosphorylation | biological_process | 1 | 1 | 0 | 4.24 | 0.98792 | 1 | AHA_1770 |
| GO:0009108 | coenzyme biosynthetic process | biological_process | 1 | 1 | 0 | 4.52 | 0.99111 | 1 | AHA_4070 |
| GO:0003824 | catalytic activity | molecular_function | 81 | 56 | 25 | 93.51 | 0.9923 | 1 | AHA_0238,AHA_0262,AHA_0278,AHA_0288,AHA_0289,AHA_0292,AHA_0377,AHA_0378,AHA_0379,AHA_0380,AHA_0382,AHA_0481,AHA_0596,AHA_0608,AHA_0612,AHA_0617,AHA_0724,AHA_0735,AHA_0803,AHA_0988,AHA_1008,AHA_1015,AHA_1040,AHA_1110,AHA_1153,AHA_1170,AHA_1172,AHA_1248,AHA_1270,AHA_1331,AHA_1413,AHA_1419,AHA_1595,AHA_1652,AHA_1654,AHA_1770,AHA_1921,AHA_1948,AHA_1956,AHA_2060,AHA_2130,AHA_2163,AHA_2296,AHA_2333,AHA_2338,AHA_2360,AHA_2405,AHA_2536,AHA_2537,AHA_2713,AHA_2715,AHA_2807,AHA_2837,AHA_2851,AHA_2923,AHA_2926,AHA_2928,AHA_2940,AHA_3263,AHA_3415,AHA_3479,AHA_3545,AHA_3601,AHA_3603,AHA_3740,AHA_3875,AHA_3935,AHA_3949,AHA_3987,AHA_4006,AHA_4008,AHA_4068,AHA_4070,AHA_4071,AHA_4114,AHA_4115,AHA_4145,AHA_4201,AHA_4258,AHA_4261,AHA_4285 |
| GO:0035556 | intracellular signal transduction | biological_process | 2 | 0 | 2 | 6.74 | 0.99299 | 1 | AHA_1213,AHA_1338 |
| GO:0004871 | signal transducer activity | molecular_function | 4 | 2 | 2 | 10.07 | 0.99303 | 1 | AHA_1213,AHA_1338,AHA_1391,AHA_3561 |
| GO:0060089 | molecular transducer activity | molecular_function | 4 | 2 | 2 | 10.07 | 0.99303 | 1 | AHA_1213,AHA_1338,AHA_1391,AHA_3561 |
| GO:0016301 | kinase activity | molecular_function | 3 | 0 | 3 | 8.74 | 0.99442 | 1 | AHA_1419,AHA_4006,AHA_4008 |
| GO:0016740 | transferase activity | molecular_function | 21 | 10 | 11 | 32.32 | 0.99447 | 1 | AHA_0481,AHA_0724,AHA_0803,AHA_0988,AHA_1040,AHA_1170,AHA_1419,AHA_1654,AHA_1921,AHA_1956,AHA_2130,AHA_2807,AHA_3263,AHA_3415,AHA_3479,AHA_3603,AHA_3740,AHA_3935,AHA_4006,AHA_4008,AHA_4070 |
| GO:0007165 | signal transduction | biological_process | 3 | 0 | 3 | 9.1 | 0.99601 | 1 | AHA_1213,AHA_1338,AHA_2046 |
| GO:0051186 | cofactor metabolic process | biological_process | 2 | 1 | 1 | 7.36 | 0.99614 | 1 | AHA_3949,AHA_4070 |
| GO:0023052 | signaling | biological_process | 3 | 0 | 3 | 9.19 | 0.99634 | 1 | AHA_1213,AHA_1338,AHA_2046 |
| GO:0044700 | single organism signaling | biological_process | 3 | 0 | 3 | 9.19 | 0.99634 | 1 | AHA_1213,AHA_1338,AHA_2046 |
| GO:0051536 | iron-sulfur cluster binding | molecular_function | 1 | 1 | 0 | 5.39 | 0.99645 | 1 | AHA_3263 |
| GO:0051540 | metal cluster binding | molecular_function | 1 | 1 | 0 | 5.39 | 0.99645 | 1 | AHA_3263 |
| GO:0051188 | cofactor biosynthetic process | biological_process | 1 | 1 | 0 | 5.97 | 0.9981 | 1 | AHA_4070 |
| GO:0007154 | cell communication | biological_process | 3 | 0 | 3 | 10.73 | 0.9991 | 1 | AHA_1213,AHA_1338,AHA_2046 |
| GO:0016772 | transferase activity, transferring phosphorus-containing groups | molecular_function | 4 | 1 | 3 | 12.89 | 0.99937 | 1 | AHA_1419,AHA_3603,AHA_4006,AHA_4008 |
| GO:0003674 | molecular_function | molecular_function | 136 | 91 | 45 | 136 | 1 | 1 | AHA_0021,AHA_0238,AHA_0262,AHA_0278,AHA_0286,AHA_0288,AHA_0289,AHA_0290,AHA_0292,AHA_0315,AHA_0377,AHA_0378,AHA_0379,AHA_0380,AHA_0382,AHA_0481,AHA_0596,AHA_0608,AHA_0612,AHA_0617,AHA_0676,AHA_0677,AHA_0724,AHA_0735,AHA_0803,AHA_0847,AHA_0859,AHA_0860,AHA_0988,AHA_1008,AHA_1015,AHA_1040,AHA_1110,AHA_1153,AHA_1170,AHA_1172,AHA_1201,AHA_1213,AHA_1248,AHA_1270,AHA_1331,AHA_1338,AHA_1339,AHA_1391,AHA_1413,AHA_1419,AHA_1522,AHA_1595,AHA_1652,AHA_1654,AHA_1687,AHA_1713,AHA_1738,AHA_1770,AHA_1805,AHA_1834,AHA_1866,AHA_1921,AHA_1948,AHA_1956,AHA_1960,AHA_2060,AHA_2093,AHA_2130,AHA_2163,AHA_2296,AHA_2305,AHA_2333,AHA_2338,AHA_2350,AHA_2360,AHA_2405,AHA_2423,AHA_2490,AHA_2536,AHA_2537,AHA_2538,AHA_2561,AHA_2713,AHA_2715,AHA_2743,AHA_2744,AHA_2806,AHA_2807,AHA_2809,AHA_2812,AHA_2813,AHA_2835,AHA_2837,AHA_2851,AHA_2923,AHA_2926,AHA_2928,AHA_2940,AHA_2982,AHA_2983,AHA_2984,AHA_3000,AHA_3004,AHA_3053,AHA_3125,AHA_3263,AHA_3324,AHA_3415,AHA_3479,AHA_3545,AHA_3561,AHA_3601,AHA_3603,AHA_3604,AHA_3606,AHA_3728,AHA_3740,AHA_3769,AHA_3821,AHA_3874,AHA_3875,AHA_3877,AHA_3935,AHA_3949,AHA_3987,AHA_4006,AHA_4008,AHA_4031,AHA_4067,AHA_4068,AHA_4070,AHA_4071,AHA_4114,AHA_4115,AHA_4145,AHA_4201,AHA_4258,AHA_4261,AHA_4267,AHA_4285 |
| GO:0005575 | cellular_component | cellular_component | 106 | 77 | 29 | 106 | 1 | 1 | AHA_0007,AHA_0008,AHA_0021,AHA_0022,AHA_0238,AHA_0262,AHA_0278,AHA_0286,AHA_0289,AHA_0290,AHA_0292,AHA_0295,AHA_0315,AHA_0377,AHA_0379,AHA_0380,AHA_0452,AHA_0596,AHA_0608,AHA_0617,AHA_0625,AHA_0676,AHA_0677,AHA_0724,AHA_0803,AHA_0847,AHA_0857,AHA_0859,AHA_0860,AHA_0913,AHA_0952,AHA_0988,AHA_1008,AHA_1040,AHA_1055,AHA_1170,AHA_1213,AHA_1246,AHA_1338,AHA_1391,AHA_1419,AHA_1522,AHA_1595,AHA_1652,AHA_1687,AHA_1713,AHA_1738,AHA_1770,AHA_1805,AHA_1866,AHA_1921,AHA_1964,AHA_2046,AHA_2130,AHA_2305,AHA_2333,AHA_2350,AHA_2405,AHA_2452,AHA_2487,AHA_2490,AHA_2628,AHA_2637,AHA_2715,AHA_2743,AHA_2806,AHA_2809,AHA_2812,AHA_2813,AHA_2835,AHA_2837,AHA_2940,AHA_2976,AHA_2982,AHA_2984,AHA_3004,AHA_3053,AHA_3125,AHA_3263,AHA_3324,AHA_3379,AHA_3477,AHA_3479,AHA_3561,AHA_3604,AHA_3606,AHA_3728,AHA_3740,AHA_3769,AHA_3820,AHA_3821,AHA_3877,AHA_3935,AHA_3949,AHA_3987,AHA_4006,AHA_4008,AHA_4031,AHA_4067,AHA_4068,AHA_4071,AHA_4114,AHA_4115,AHA_4261,AHA_4267,AHA_4285 |
| GO:0008150 | biological_process | biological_process | 126 | 87 | 39 | 126 | 1 | 1 | AHA_0007,AHA_0008,AHA_0022,AHA_0238,AHA_0262,AHA_0278,AHA_0286,AHA_0288,AHA_0290,AHA_0292,AHA_0295,AHA_0315,AHA_0377,AHA_0378,AHA_0379,AHA_0380,AHA_0382,AHA_0452,AHA_0481,AHA_0596,AHA_0608,AHA_0612,AHA_0617,AHA_0676,AHA_0677,AHA_0724,AHA_0735,AHA_0803,AHA_0847,AHA_0859,AHA_0860,AHA_0913,AHA_0988,AHA_1008,AHA_1015,AHA_1040,AHA_1110,AHA_1153,AHA_1170,AHA_1172,AHA_1201,AHA_1213,AHA_1246,AHA_1248,AHA_1270,AHA_1338,AHA_1339,AHA_1391,AHA_1413,AHA_1419,AHA_1522,AHA_1652,AHA_1654,AHA_1713,AHA_1738,AHA_1770,AHA_1805,AHA_1834,AHA_1921,AHA_1956,AHA_1960,AHA_1964,AHA_2046,AHA_2060,AHA_2093,AHA_2130,AHA_2163,AHA_2305,AHA_2333,AHA_2338,AHA_2405,AHA_2490,AHA_2536,AHA_2538,AHA_2561,AHA_2637,AHA_2713,AHA_2715,AHA_2743,AHA_2744,AHA_2807,AHA_2812,AHA_2813,AHA_2835,AHA_2837,AHA_2851,AHA_2923,AHA_2926,AHA_2982,AHA_2983,AHA_2984,AHA_3000,AHA_3004,AHA_3053,AHA_3324,AHA_3415,AHA_3545,AHA_3561,AHA_3601,AHA_3604,AHA_3606,AHA_3728,AHA_3740,AHA_3820,AHA_3821,AHA_3874,AHA_3875,AHA_3877,AHA_3935,AHA_3949,AHA_3987,AHA_4006,AHA_4008,AHA_4031,AHA_4067,AHA_4068,AHA_4070,AHA_4071,AHA_4114,AHA_4115,AHA_4145,AHA_4201,AHA_4258,AHA_4261,AHA_4267,AHA_4285 |
