# Supplementary material for: Transcriptome differences between enrofloxacin-resistant and enrofloxacin-susceptible strains of Aeromonas hydrophila
Source: PLoS One. 2017 Jul 14;12(7):e0179549. doi: 10.1371/journal.pone.0179549 (PMC5510800; doi:10.1371/journal.pone.0179549)
Supplement: S2 Table — (DOCX) [file pone.0179549.s002.docx]

**S2 Table. KEGG pathway analysis for the differential express genes of the *Aeromonas hydrophilia*.**

| **KO_ID** | **Term** | **Type** | **DEGs_this_term** | **UP** | **Down** | **Pvalue** | **FDR** | **DEGs_list** |
| --- | --- | --- | --- | --- | --- | --- | --- | --- |
| ko00340 | Histidine metabolism | Metabolism | 4 | 4 | 0 | 0.002649759 | 0.177533853 | AHA_0379,AHA_0378,AHA_0377,AHA_0380 |
| ko05152 | Tuberculosis | Human Diseases | 2 | 2 | 0 | 0.006813451 | 0.228250609 | AHA_2983,AHA_0860 |
| ko00473 | D-Alanine metabolism | Metabolism | 2 | 1 | 1 | 0.01319806 | 0.294756673 | AHA_2536,AHA_1015 |
| ko00400 | Phenylalanine | Metabolism | 4 | 3 | 1 | 0.01944488 | 0.324879032 | AHA_2923,AHA_2926,AHA_1654,AHA_2928 |
| ko04141 | Protein processing in endoplasmic reticulum | Genetic Information Processing | 1 | 1 | 0 | 0.04878049 | 0.324879032 | AHA_2490 |
| ko04151 | PI3K-Akt signaling pathway | Environmental Information Processing | 1 | 1 | 0 | 0.04878049 | 0.324879032 | AHA_2490 |
| ko04612 | Antigen processing and presentation | Organismal Systems | 1 | 1 | 0 | 0.04878049 | 0.324879032 | AHA_2490 |
| ko04914 | Progesterone-mediated oocyte maturation | Organismal Systems | 1 | 1 | 0 | 0.04878049 | 0.324879032 | AHA_2490 |
| ko04915 | Estrogen signaling pathway | Organismal Systems | 1 | 1 | 0 | 0.04878049 | 0.324879032 | AHA_2490 |
| ko05215 | Prostate cancer | Human Diseases | 1 | 1 | 0 | 0.04878049 | 0.324879032 | AHA_2490 |
| ko00195 | Photosynthesis | Metabolism | 2 | 2 | 0 | 0.05425991 | 0.324879032 | AHA_4261,AHA_4267 |
| ko03060 | Protein export | Genetic Information Processing | 3 | 3 | 0 | 0.06139866 | 0.324879032 | AHA_0295,AHA_1738,AHA_3877 |
| ko01230 | Biosynthesis of amino acids | Metabolism | 10 | 7 | 3 | 0.06303623 | 0.324879032 | AHA_4201,AHA_2923,AHA_1956,AHA_1170,AHA_2928,AHA_0596,AHA_4068,AHA_2360,AHA_2926,AHA_1654 |
| ko04621 | NOD-like receptor signaling pathway | Organismal Systems | 1 | 1 | 0 | 0.09521576 | 0.398715995 | AHA_2490 |
| ko04940 | Type I diabetes mellitus | Human Diseases | 1 | 1 | 0 | 0.09521576 | 0.398715995 | AHA_0860 |
| ko05200 | Pathways in cancer | Human Diseases | 1 | 1 | 0 | 0.09521576 | 0.398715995 | AHA_2490 |
| ko00785 | Lipoic acid metabolism | Metabolism | 1 | 1 | 0 | 0.1394169 | 0.518940683 | AHA_3263 |
| ko00830 | Retinol metabolism | Metabolism | 1 | 0 | 1 | 0.1394169 | 0.518940683 | AHA_1331 |
| ko02010 | ABC transporters | Environmental Information Processing | 9 | 9 | 0 | 0.1582458 | 0.530103043 | AHA_0608,AHA_2812,AHA_0913,AHA_3728,AHA_1964,AHA_1687,AHA_4285,AHA_1595,AHA_2813 |
| ko00561 | Glycerolipid metabolism | Metabolism | 2 | 1 | 1 | 0.163769 | 0.530103043 | AHA_3740,AHA_4008 |
| ko00270 | Cysteine and methionine metabolism | Metabolism | 3 | 1 | 2 | 0.1769284 | 0.530103043 | AHA_3479,AHA_2807,AHA_1956 |
| ko00250 | Alanine | Metabolism | 3 | 3 | 0 | 0.2021597 | 0.530103043 | AHA_1413,AHA_0596,AHA_4068 |
| ko00190 | Oxidative phosphorylation | Metabolism | 4 | 4 | 0 | 0.2133528 | 0.530103043 | AHA_4261,AHA_1770,AHA_4267,AHA_2296 |
| ko00260 | Glycine | Metabolism | 3 | 2 | 1 | 0.2150891 | 0.530103043 | AHA_4201,AHA_2360,AHA_2928 |
| ko03018 | RNA degradation | Genetic Information Processing | 2 | 2 | 0 | 0.2178489 | 0.530103043 | AHA_2983,AHA_0860 |
| ko00625 | Chloroalkane and chloroalkene degradation | Metabolism | 1 | 0 | 1 | 0.2215356 | 0.530103043 | AHA_1331 |
| ko00626 | Naphthalene degradation | Metabolism | 1 | 0 | 1 | 0.2215356 | 0.530103043 | AHA_1331 |
| ko01220 | Degradation of aromatic compounds | Metabolism | 1 | 0 | 1 | 0.2215356 | 0.530103043 | AHA_1331 |
| ko00550 | Peptidoglycan biosynthesis | Metabolism | 2 | 1 | 1 | 0.2362548 | 0.543642816 | AHA_3935,AHA_2130 |
| ko00030 | Pentose phosphate pathway | Metabolism | 2 | 2 | 0 | 0.2547512 | 0.543642816 | AHA_0289,AHA_0288 |
| ko00460 | Cyanoamino acid metabolism | Metabolism | 1 | 1 | 0 | 0.2596503 | 0.543642816 | AHA_4068 |
| ko05134 | Legionellosis | Human Diseases | 1 | 1 | 0 | 0.2596503 | 0.543642816 | AHA_0860 |
| ko00564 | Glycerophospholipid metabolism | Metabolism | 2 | 1 | 1 | 0.2732862 | 0.5548538 | AHA_3740,AHA_1652 |
| ko03070 | Bacterial secretion system | Environmental Information Processing | 3 | 3 | 0 | 0.2819398 | 0.555587253 | AHA_0295,AHA_1738,AHA_3877 |
| ko00051 | Fructose and mannose metabolism | Metabolism | 2 | 0 | 2 | 0.291813 | 0.558613457 | AHA_2338,AHA_1419 |
| ko04626 | Plant-pathogen interaction | Organismal Systems | 1 | 1 | 0 | 0.3304495 | 0.615003236 | AHA_2490 |
| ko00980 | Metabolism of xenobiotics by cytochrome P450 | Metabolism | 1 | 0 | 1 | 0.3633048 | 0.627123092 | AHA_1331 |
| ko00982 | Drug metabolism - cytochrome P450 | Metabolism | 1 | 0 | 1 | 0.3633048 | 0.627123092 | AHA_1331 |
| ko00312 | beta-Lactam resistance | Human Diseases | 2 | 2 | 0 | 0.3650418 | 0.627123092 | AHA_0021,AHA_0022 |
| ko00750 | Vitamin B6 metabolism | Metabolism | 1 | 1 | 0 | 0.394571 | 0.660906425 | AHA_0803 |
| ko00920 | Sulfur metabolism | Metabolism | 2 | 2 | 0 | 0.4523511 | 0.739207895 | AHA_0608,AHA_3479 |
| ko00071 | Fatty acid degradation | Metabolism | 1 | 0 | 1 | 0.529597 | 0.750429902 | AHA_1331 |
| ko00290 | Valine | Metabolism | 1 | 1 | 0 | 0.529597 | 0.750429902 | AHA_4201 |
| ko00350 | Tyrosine metabolism | Metabolism | 1 | 0 | 1 | 0.529597 | 0.750429902 | AHA_1331 |
| ko00670 | One carbon pool by folate | Metabolism | 1 | 0 | 1 | 0.529597 | 0.750429902 | AHA_3949 |
| ko00061 | Fatty acid biosynthesis | Metabolism | 1 | 1 | 0 | 0.5528007 | 0.750429902 | AHA_1040 |
| ko00450 | Selenocompound metabolism | Metabolism | 1 | 0 | 1 | 0.5528007 | 0.750429902 | AHA_1956 |
| ko00300 | Lysine biosynthesis | Metabolism | 1 | 1 | 0 | 0.5748764 | 0.750429902 | AHA_1170 |
| ko04122 | Sulfur relay system | Genetic Information Processing | 1 | 1 | 0 | 0.5748764 | 0.750429902 | AHA_3479 |
| ko00330 | Arginine and proline metabolism | Metabolism | 2 | 2 | 0 | 0.5769896 | 0.750429902 | AHA_1270,AHA_0596 |
| ko02040 | Flagellar assembly | Cellular Processes | 2 | 2 | 0 | 0.5910784 | 0.750429902 | AHA_2837,AHA_2835 |
| ko00760 | Nicotinate and nicotinamide metabolism | Metabolism | 1 | 1 | 0 | 0.595878 | 0.750429902 | AHA_1008 |
| ko01200 | Carbon metabolism | Metabolism | 5 | 3 | 2 | 0.6018749 | 0.750429902 | AHA_4201,AHA_3949,AHA_0289,AHA_0288,AHA_2360 |
| ko00010 | Glycolysis / Gluconeogenesis | Metabolism | 2 | 0 | 2 | 0.6048241 | 0.750429902 | AHA_2360,AHA_1331 |
| ko00480 | Glutathione metabolism | Metabolism | 1 | 1 | 0 | 0.6529415 | 0.795401464 | AHA_1270 |
| ko00630 | Glyoxylate and dicarboxylate metabolism | Metabolism | 1 | 1 | 0 | 0.7020522 | 0.829180408 | AHA_0289 |
| ko01212 | Fatty acid metabolism | Metabolism | 1 | 1 | 0 | 0.730921 | 0.829180408 | AHA_1040 |
| ko00680 | Methane metabolism | Metabolism | 1 | 0 | 1 | 0.744304 | 0.829180408 | AHA_2360 |
| ko03010 | Ribosome | Genetic Information Processing | 2 | 2 | 0 | 0.7530021 | 0.829180408 | AHA_0315,AHA_4031 |
| ko00520 | Amino sugar and nucleotide sugar metabolism | Metabolism | 2 | 1 | 1 | 0.7621828 | 0.829180408 | AHA_3935,AHA_2338 |
| ko00230 | Purine metabolism | Metabolism | 3 | 3 | 0 | 0.7633082 | 0.829180408 | AHA_1008,AHA_2333,AHA_1413 |
| ko02060 | Phosphotransferase system (PTS) | Environmental Information Processing | 1 | 0 | 1 | 0.7691336 | 0.829180408 | AHA_1419 |
| ko00240 | Pyrimidine metabolism | Metabolism | 2 | 2 | 0 | 0.7796771 | 0.829180408 | AHA_1008,AHA_2333 |
| ko00720 | Carbon fixation pathways in prokaryotes | Metabolism | 1 | 0 | 1 | 0.811883 | 0.849940016 | AHA_3949 |
| ko02020 | Two-component system | Environmental Information Processing | 5 | 5 | 0 | 0.8715394 | 0.898355997 | AHA_2454,AHA_1391,AHA_2453,AHA_2452,AHA_2296 |
| ko00620 | Pyruvate metabolism | Metabolism | 1 | 1 | 0 | 0.9085947 | 0.922361286 | AHA_4145 |
| ko02030 | Bacterial chemotaxis | Cellular Processes | 1 | 1 | 0 | 0.9623575 | 0.9623575 | AHA_1391 |
