# Supplementary figures and images for: Transcriptome differences between enrofloxacin-resistant and enrofloxacin-susceptible strains of Aeromonas hydrophila
Source: PLoS One. 2017 Jul 14;12(7):e0179549. doi: 10.1371/journal.pone.0179549 (PMC5510800; doi:10.1371/journal.pone.0179549)

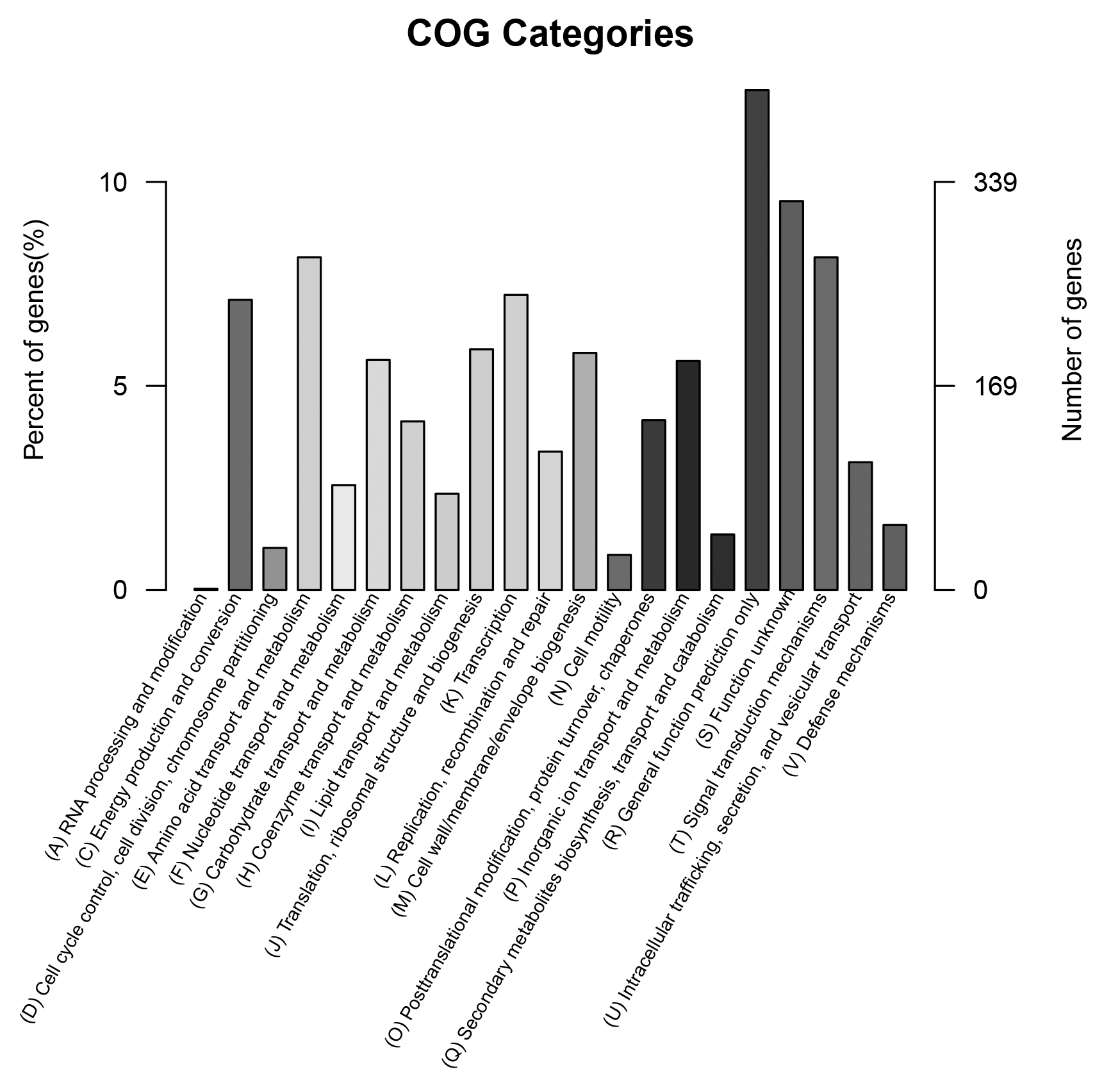

Supplement: S1 Fig — (TIF) [file pone.0179549.s003.tif]

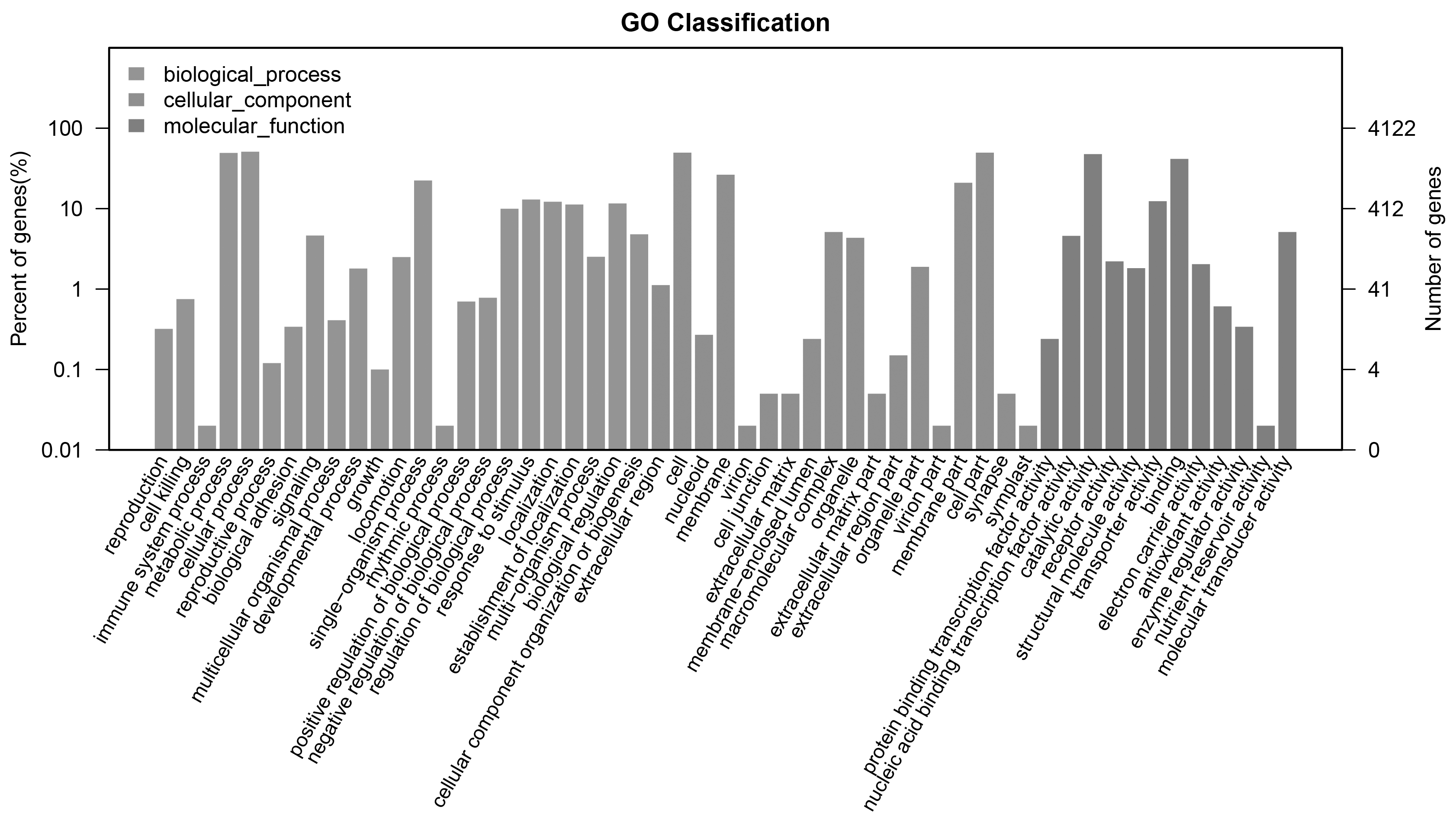

Supplement: S2 Fig — Categories (X axis) were grouped into three main ontologies: biological process, cellular component, and molecular function. The Y axis indicates the percentage of genes (%). (TIF) [file pone.0179549.s004.tif]

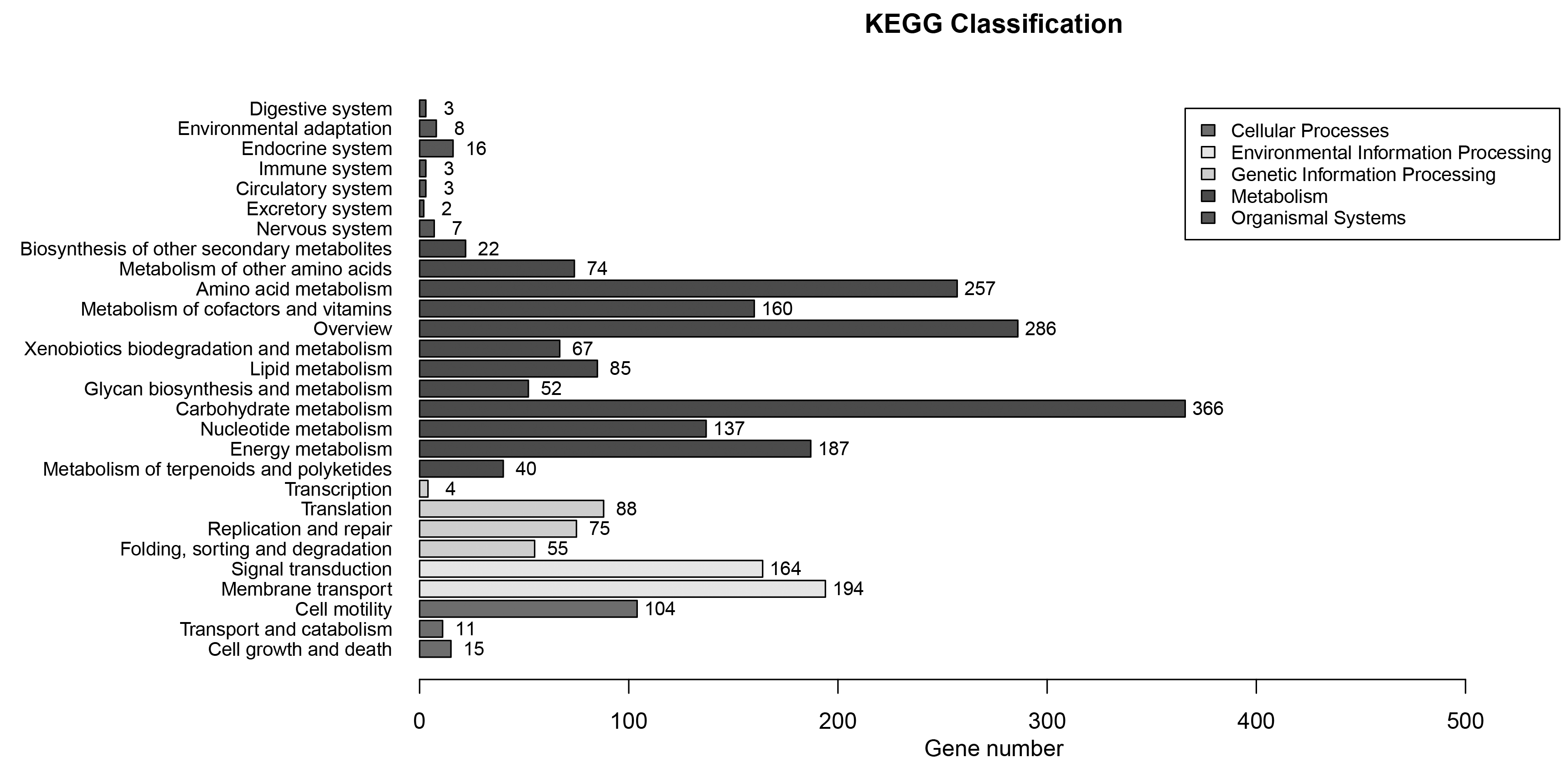

Supplement: S3 Fig — X axis, KEGG pathway categories; Y axis, statistical significance of enrichment. (TIF) [file pone.0179549.s005.tif]
